# External validation of dementia risk prediction models in the EPIC-Norfolk cohort: a UK-based cohort study

**DOI:** 10.1038/s44400-026-00095-7

**Published:** 2026-06-19

**Authors:** Jacob Brain, Eugene YH Tang, Claire V. Burley, Jennifer Dunne, Grazziela Figueredo, Leanne Greene, Mario Siervo, Phillip J. Tully, Deborah Turnbull, Zhongyang Guan, Ruth H. Jack, Blossom CM Stephan

**Affiliations:** 1 Institute of Mental Health, School of Medicine, University of Nottingham, Innovation Park, Jubilee Campus, Nottingham, UK; 2 Freemasons Foundation Centre for Men’s Health, Discipline of Medicine, School of Psychology, The University of Adelaide, Adelaide, SA Australia; 3 Population Health Sciences Institute, Newcastle University, Newcastle upon Tyne, UK; 4 Dementia Centre of Excellence, Curtin enAble Institute, Faculty of Health Sciences, Curtin University, Perth, WA Australia; 5 Centre for Health Informatics, School of Medicine, University of Nottingham, Nottingham, NG7 2TU UK; 6 Department of Health and Community Sciences, Faculty of Health and Life Sciences, University of Exeter, Exeter, UK; 7 School of Population Health, Curtin University, Perth, WA Australia; 8 Faculty of Medicine and Health, School of Psychology, Deakin University, Waurn Ponds, Geelong, VIC Australia; 9 Centre for Academic Primary Care, Lifespan and Population Health, School of Medicine, University of Nottingham, Nottingham, UK

**Keywords:** Diseases, Medical research, Neurology, Risk factors

## Abstract

External validation of dementia risk models is essential to assess generalisability and clinical utility. We evaluated 12 prediction models, including 10 dementia-specific and two cardiovascular-based models, in the EPIC-Norfolk cohort (*n* = 25,423) with up to 30 years of follow-up. Performance was assessed using discrimination, calibration, competing risks, and power analyses, stratified by follow-up and sex. Five models were fully validated and seven partially. The CAIDE, CAIDE-APOE, and DRS showed moderate performance declines, while the FRS and CHA₂DS₂-VASc demonstrated good calibration and strong transportability, supporting the value of vascular risk factors in predicting dementia. The UKBDRS maintained high discrimination (>0.80) despite partial validation. Performance was generally lower in women, especially for CAIDE and DRS. Calibration was acceptable in most fully validated models, though several were underpowered. Simplified models focusing on core vascular and lifestyle predictors may offer scalable, clinically relevant tools for dementia risk stratification.

## Introduction

The escalating global prevalence of dementia, characterised by its profound impact on individuals, families, and health and social care systems, underscores the urgent need for effective risk reduction and preventative strategies^1^. Traditionally, dementia research has focused on pharmacological interventions post-diagnosis, but the shift towards prevention and early detection reflects a pivotal change in aiming to reduce risk or delay the onset of this condition^1^. In this context, the development and validation of risk prediction models have become critical to identifying high-risk individuals who could benefit from early intervention^2^.

Dementia risk prediction models generally integrate various socio-demographic, genetic, and health-related factors into a single algorithm to estimate an individual’s likelihood of developing the condition. However, the application of these models in clinical and public health settings is contingent upon their external validation, acceptability, and cost-effectiveness, meaning the process by which a model’s performance is tested on data independent of that used in its development. External validation assesses the generalisability and robustness of a model across different populations and settings, which is crucial given the variability in dementia risk across ethnicities, geographies, and socioeconomic groups^3–5^. Despite the proliferation of dementia risk models, there remains a significant gap in their validation^2,6^. Many models are developed in homogeneous or specific populations, often in high-income countries, and may not perform well when applied to broader, more varied populations^7^. This gap highlights the need for rigorous testing of these models in large-scale, population-based cohorts that capture a wide spectrum of risk factors and demographic variables.

The current study aims to externally validate 10 dementia risk prediction models and two cardiovascular-based models, using data from the European Prospective Investigation into Cancer in Norfolk (EPIC-Norfolk) cohort^8^. The cardiovascular models were included due to their potential for repurposing in dementia risk prediction, given the strong vascular contributions to cognitive decline. By examining model performance across different follow-up periods, sexes, and employing time-to-event and competing risks methodologies, we aim to provide a nuanced assessment of these models’ generalisability.

## Results

Of the 25,636 participants who attended the baseline health check, 25,423 without dementia at baseline were included in the analyses (Figure. S1). The cohort had a mean age of 59.3 years (SD 9.3), with 54.7% of the sample being women. At baseline, 17,299 participants (68.1%) were classified as being in midlife (<65 years), while 8,124 (31.9%) were in late life (≥65 years). The mean follow-up time was 21.3 years (median: 25.1, IQR: 16.2–27.0 years, full range: 30.0 years). Over the whole follow-up period, 3,377 participants (13.3%) developed dementia, with a mean age at dementia onset of 85.2 years (SD 6.4). The mean time from baseline to dementia diagnosis was 19.4 years (median: 20.3 years, IQR: 15.7–23.8 years). Table 1 provides the baseline characteristics of the analytic cohort.

**Table 1:** Baseline characteristics of the EPIC-Norfolk cohort^*^

	Total, *N* (%)	Male, *N* (%)	Female, *N* (%)
*N*	25,423	11,519	13,904
All-cause dementia	3377	1318	2059
Mean age at baseline (SD)	59.29 (9.32)	59.69 (9.30)	58.97 (9.32)
Education (%)			
No qualifications	9417 (37.04)	3520 (30.56)	5897 (42.41)
O-level	2600 (10.23)	998 (8.66)	1602 (11.52)
A-level	10,150 (39.92)	5236 (45.46)	9414 (35.34)
Degree	3238 (12.74)	1756 (15.24)	1482 (10.66)
Missing	18 (0.07)	9 (0.08)	9 (0.06)
Marital status			
Single	1,013 (3.98)	468 (4.06)	545 (3.92)
Married	20,546 (80.82)	10,013 (86.93)	10,533 (75.76)
Widowed	1955 (7.69)	366 (3.18)	1589 (11.43)
Separated/divorced	1761 (6.93)	611 (5.30)	1150 (8.27)
Missing	148 (0.58)	61 (0.53)	87 (0.63)
BMI			
< 30 kg/m^2^	21,423 (84.27)	9929 (86.20)	11,494 (82.67)
≥ 30 kg/m^2^	3941 (15.50)	1564 (13.58)	2377 (17.10)
Missing	59 (0.23)	26 (0.23)	33 (0.24)
Physical activity			
Inactive	15,104 (59.41)	6402 (55.58)	8702 (62.59)
Active	10,319 (40.59)	5117 (44.42)	5202 (37.41)
Smoking status			
Never	11,567 (45.50)	3800 (32.99)	7767 (55.86)
Former	10,675 (41.99)	6242 (54.19)	4433 (31.88)
Current	2961 (11.65)	1396 (12.12)	1565 (11.26)
Missing	220 (0.87)	81 (0.70)	139 (1.00)
Alcohol consumption			
< 14 units per week	21,018 (82.67)	8378 (72.73)	12,640 (90.91)
≥ 14 units per week	4137 (16.27)	3055 (26.52)	1082 (7.78)
Missing	268 (1.05)	86 (0.75)	182 (1.31)
Hypertension or antihypertensive use			
No	12,649 (49.75)	5292 (45.94)	7357 (52.91)
Yes	12,717 (50.02)	6206 (53.88)	6511 (46.83)
Missing	57 (0.22)	21 (0.18)	36 (0.26)
High cholesterol (self-reported statin use or ≥6.2 mmol/L total cholesterol)			
No	16,330 (64.23)	7893 (68.52)	8437 (60.68)
Yes	9093 (35.77)	3626 (31.48)	5467 (39.32)
Diabetes			
No	24,501 (96.37)	10,978 (95.30)	13,523 (97.26)
Yes	890 (3.50)	525 (4.56)	365 (2.63)
Missing	32 (0.13)	16 (0.14)	16 (0.12)
Stroke			
No	25,036 (98.48)	11,294 (98.05)	13,742 (98.83)
Yes	362 (1.42)	212 (1.84)	150 (1.08)
Missing	25 (0.10)	13 (0.11)	12 (0.09)
Traumatic brain injury			
No	25,420 ( ~ 100.00)	11,515 (99.99)	13,905 (100.00)
Yes	< 10 (-)	< 10 (-)	0
Self-reported depression (requiring treatment) or antidepressant use			
No	21,308 (83.81)	10,282 (89.26)	11,026 (79.30)
Yes	4068 (16.00)	1220 (10.59)	2848 (20.48)
Missing	47 (0.18)	17 (0.15)	30 (0.22)
Atrial fibrillation			
No	24,000 (94.40)	10,912 (94.73)	13,086 (94.12)
Yes	1410 (5.50)	600 (5.20)	810 (5.80)
Missing	16 (0.10)	< 10 (-)	< 10 (-)
Coronary heart disease (including angina and myocardial infarction)			
No	23,702 (93.23)	10,374 (90.06)	13,328 (95.86)
Yes	1675 (6.59)	1120 (9.72)	555 (3.99)
Missing	46 (0.18)	25 (0.22)	21 (0.15)
Renal dysfunction			
No	25,423 (100.00)	11,519 (100.00)	13,904 (100.00)
Yes	0	0	0
Sleep problems requiring treatment			
No	24,493 (96.34)	11,502 (99.85)	12,991 (93.43)
Yes	893 (3.51)	0	893 (6.42)
Missing	37 (0.15)	17 (0.15)	20 (0.14)
APOE e4 genotype			
Non-carrier	14,931 (58.73)	6972 (60.53)	7959 (57.24)
Carrier	5935 (23.35)	2745 (23.83)	3190 (22.94)
Missing	4557 (17.92)	1802 (15.64)	2755 (19.81)

^*^Cell counts have been rounded to the nearest 5 where necessary to prevent disclosure. Cell sizes < 10 are suppressed.

Loss to follow-up before administrative censoring was low, occurring in 3.7% of participants by 25 years of follow-up (Fig. S2). Dropout rates were similar across sex (3.5% in men and 3.8% in women; *p* = 0.39) and deprivation quintiles (3.5–3.7%; *p* = 0.99), with slightly lower dropout among participants aged ≥ 65 years (1.4%) compared with those <65 years (4.7%).

Apolipoprotein E (APOE) genotype data were available for 20,866 participants (82.1% of the analytic cohort). Dementia incidence increased with ε4 copy number, occurring in 10.4% of ε4 non-carriers, 19.1% of heterozygous carriers, and 31.4% of ε4 homozygotes. Exploratory analyses examining risk score distributions and event rates across APOE ε4 strata for APOE-inclusive models are presented in Table S1.

### Full model validation and calibration

**Fig. 1:**
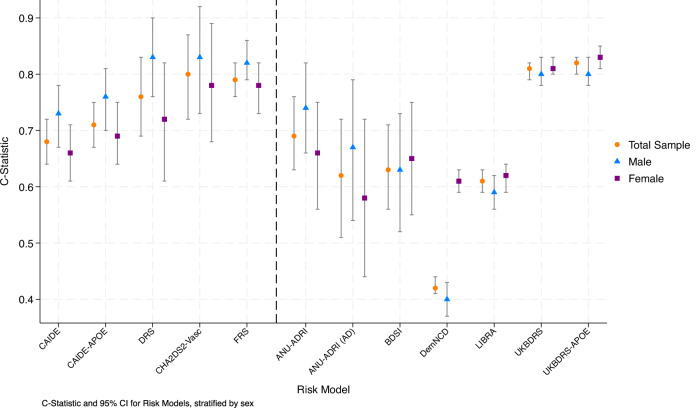
Discriminative performance of risk models in the EPIC-Norfolk cohort, stratified by sex. C-statistic and 95% CI for each model, separately for the total sample (orange), males (blue), and females (purple). Models to the left of the dashed vertical line represent those that underwent full external validation, while those to the right represent partial validations.

In this external validation study, five models underwent full validation and demonstrated varying degrees of consistency with the original development C-statistics (Table 2, Fig. 1). The Cardiovascular Risk Factors, Ageing, and Dementia Risk Score (CAIDE) and CAIDE-APOE models showed moderate declines from their development C-statistics of 0.77 (95% CI: 0.71–0.83) to predictive performance in EPIC-Norfolk of 0.68 (95% CI: 0.64–0.72) and 0.78 (95% CI: 0.72–0.84) to 0.71 (95% CI: 0.67–0.75), respectively, suggesting some attenuation in predictive performance, although confidence intervals overlapped between development and validation estimates. The Dementia Risk Score (DRS), which had the highest development C-statistic (0.84, 95% CI: 0.81–0.87), exhibited a moderate decline to 0.76 (95% CI: 0.69–0.83). Notably, the CHA_2_DS_2_-VASc model, with a development C-statistic of 0.61 (95% CI: 0.51–0.70), showed a substantial increase to 0.80 (95% CI: 0.72–0.87) in EPIC-Norfolk. In contrast, the Framingham Risk Score (FRS) showed excellent transportability, with a development range of 0.75–0.79 and a near identical C-statistic of 0.79 (95% CI: 0.76–0.82) in EPIC-Norfolk.

**Table 2:** External validation of risk models

Model	Dementia outcome	Age eligibility	Follow-up (years)	Statistical analysis	Validation (missing variables)	Development C-statistic (95%CI)	n/N (death^*^)	EPIC-Norfolk C-statistic (95%CI)
ANU-ADRI	All-cause	≥ 60	6	Cox proportional hazards model	Partial (2: Social engagement, cognitive activity)	0.65 (0.62–0.71) to 0.73 (0.70–0.75)	58/10,904	0.69 (0.63–0.76)
ANU-ADRI	AD	≥ 60	6	Cox proportional hazards model	Partial (2: Social engagement, cognitive activity)	0.64 (0.60–0.68) to 0.74 (0.71–0.76)	23/10,904	0.62 (0.51–0.72)
BDSI	All-cause	≥ 65	6	Cox proportional hazards model	Partial (1: Functional impairment)	0.68 (0.65–0.72) to 0.78 (0.72-0.83)	64/8076	0.63 (0.56–0.71)
CAIDE	All-cause	39–64	20	Logistic regression	Full	0.77 (0.71–0.83)	169/13,132	0.68 (0.64–0.72)
CAIDE-APOE	All-cause	39–64	20	Logistic regression	Full	0.78 (0.72–0.84)	144/11,402	0.71 (0.67–0.75)
DemNCD	All-cause	≥ 65	Median follow-up = 16.5 years, mean = 16.08 yearsUp to 29.8 years follow-up	Fine-Gray sub-distribution model	Partial (3: Fruit and vegetable intake, loneliness, cognitive activity)	0.68 (0.65–0.70)	1385/5595 (3703)	0.42 (0.41–0.44)
DRS	All-cause	60–79	5	Cox proportional hazards model	Full	0.84 (0.81–0.87)^†^	38/11,675	0.76 (0.69–0.83)
LIBRA	All-cause	≥ 50	16	Cox proportional hazards model	Partial (1: High cognitive activity)	0.60 (0.53–0.67)	774/17,980	0.61 (0.59–0.63)
UKBDRS	All-cause	≥ 50	14	Fine-Gray sub-distribution model	Partial (2: Parental history of dementia, lives alone)	0.80 (0.78–0.82)	586/19,973 (3787)	0.81 (0.79–0.82)
UKBDRS-APOE	All-cause	≥ 50	14	Fine-Gray sub-distribution model	Partial (2: Parental history of dementia, lives alone)	0.83 (0.81–0.84)	452/16,444 (2921)	0.82 (0.80–0.83)
CHA_2_DS_2_-Vasc	N/A	≥ 18	5	Logistic regression	Full	0.61 (0.51–0.70)^‡^	28/24,324	0.80 (0.72–0.87)
FRS	N/A	30–74	10	Cox proportional hazards model	Full	0.75 (0.73–0.77) to 0.79 (0.76–0.81)^§^	150/21,913	0.79 (0.76–0.82)

^*^Death as a competing risk relevant to Fine-Gray models.

†Uno’s C-statistic.

‡Development C-statistic developed for an outcome of stroke in atrial fibrillation patients.

§Development C-statistic developed for an outcome of developing cardiovascular disease including stroke.

*AD* Alzheimer’s disease, *ANU-ADRI* Australian National University Alzheimer’s Disease Risk Index, *BDSI* Brief Dementia Screening Indicator, *CAIDE* Cardiovascular Risk Factors, Aging, and Incidence of Dementia, CAIDE-APOE, CAIDE model including apolipoprotein E, *CHA₂DS₂-VASc* Congestive heart failure, Hypertension, Age ≥ 75, Diabetes, Stroke, Vascular disease, Age 65–74, Sex category, *CI* confidence interval, *DemNCD* Dementia Non-Communicable Disease Risk Score, *DRS* Dementia Risk Score, *FRS* Framingham Risk Score, *LIBRA* Lifestyle for Brain Health Score, *N/A* not applicable, *UKBDRS* UK Biobank Dementia Risk Score, *UKBDRS-APOE* UK Biobank Dementia Risk Score including apolipoprotein E.

Sex-stratified analyses revealed variations in the discriminative performance, with most models demonstrating lower predictive ability in women compared to men (Table S2). The CAIDE model, which showed a moderate decline in discrimination from the development C-statistic to full external validation, exhibited further stratification-based reductions, with 0.73 (95% CI: 0.67–0.78) in men and 0.66 (95% CI: 0.61–0.71) in women, suggesting potential sex-related miscalibration. A similar pattern was observed for CAIDE-APOE, with a C-statistic of 0.76 (95% CI: 0.70–0.81) in men and 0.69 (95% CI: 0.64–0.75) in women. The DRS maintained strong discrimination in men (0.83, 95% CI: 0.76–0.90) but showed a greater reduction in women (0.72, 95% CI: 0.61–0.82). The CHA₂DS₂-VASc remained robust in both sexes, with C-statistics of 0.83 (95% CI: 0.73–0.92) in men and 0.78 (95% CI: 0.68–0.89) in women. Similarly, the FRS demonstrated similar stability in men (0.82, 95% CI: 0.79–0.86) and women (0.78, 95% CI: 0.73–0.82).

Calibration across models indicated generally good performance (Table S3). The CAIDE and CAIDE-APOE exhibited strong calibration, with non-significant χ² statistics (*p* > 0.05) and calibration-in-the-large (CITL) values close to zero, suggesting minimal systematic miscalibration. Calibration plots further demonstrated strong agreement between predicted and observed risks. However, sex-stratified analyses revealed some divergence, with the CAIDE overestimating risk in women and underestimating in men at higher risk levels, reflected in a significant calibration difference (χ² = 17.83, *p* = 0.013 in women vs. χ² = 8.48, *p* = 0.205 in men) (Figs. S3 and S4). Recalibration of the CAIDE model in women produced a slope of 1.00 and an intercept effectively equal to zero, indicating that the model’s overall risk predictions required no adjustment. The recalibrated calibration plot confirmed good agreement between predicted and observed risks across all deciles, supporting the robustness of the model’s predictions despite the earlier statistical misfit (Table S4; Fig. S5). The DRS showed similar calibration characteristics, with a χ² of 11.23 (*p* = 0.260) and a calibration slope close to 1 (0.970), indicating good alignment between predicted and observed risks, though slight miscalibration was observed at higher predicted risk levels, particularly with underestimation in men and greater variability in women (Fig. S6). The CHA₂DS₂-VASc demonstrated excellent calibration across the total cohort and within sex-stratified analyses, with no significant deviations observed (Fig. S7). In contrast, the FRS exhibited acceptable calibration overall (χ² = 12.41, *p* = 0.191), however, CITL indicated modest overestimation of risk (− 0.0010), and the calibration slope was below unity (slope = 0.92). Calibration plots indicated a tendency for the FRS to underestimate dementia risk at higher predicted probabilities in both sexes, though alignment was slightly better in women (Fig. S8). In men, recalibration of the FRS resulted in an intercept of −8.96 and a slope of 0.89, indicating modest overestimation of dementia risk that was corrected by adjusting both the baseline risk and the spread of predicted probabilities (Table S4; Fig. S9).

The transportability of fully validated models varied across follow-up durations using Cox regression (Table S5), with the CAIDE and CAIDE-APOE demonstrating stable performance over time (C-statistics: 0.67 at 5 years to 0.67 at full follow-up for CAIDE; 0.70 at 5 years to 0.69 at full follow-up for CAIDE-APOE), as well as in both men and women. The DRS exhibited a gradual decline, particularly in men (C-statistic 0.83 at 5 years to 0.68 at full follow-up), indicating potential sex-specific differences in long-term predictive accuracy. A similar pattern was observed for CHA₂DS₂-VASc, which started with high discrimination (0.81 at 5 years) but declined to 0.71 at full follow-up, with women experiencing a steeper drop in discriminative performance. In contrast, the FRS maintained robust transportability, with minimal variation over time (0.78 at 5 years to 0.76 at full follow-up) and regardless of sex.

Time-dependent calibration analysis revealed distinct patterns across the five fully validated models (Fig. S10). The CAIDE model demonstrated improving calibration with extended follow-up, achieving optimal alignment between observed and predicted probabilities beyond 20 years, though it consistently underestimated risk at earlier time points, particularly at intermediate risk levels. Similarly, CAIDE-APOE showed progressive calibration improvement over time, but maintained notable underprediction at higher risk probabilities, especially in women. The DRS model exhibited generally good calibration throughout, with only slight overestimation at early follow-up points (5–10 years) and minimal miscalibration thereafter, performing better in males than females. The CHA₂DS₂-VASc model initially showed moderate underprediction at higher risk levels, particularly in females, but significantly improved beyond 20 years with residuals approaching zero. In contrast, the Framingham Risk Score maintained consistent calibration across all follow-up periods with only modest overprediction at higher risk deciles, particularly in males after 15 years.

Fine-Gray analyses in the total sample across the different follow-up periods showed no significant differences in discriminative performances compared to Cox regression analyses (Table S6).

### Partial model validation

Seven models underwent partial external validation due to missing variables (Table 2). The Australian National University Alzheimer’s Disease Risk Index (ANU-ADRI) lacked two key psychosocial factors, social engagement and cognitive activity, while for the Brief Dementia Screening Indicator (BDSI) we were missing functional impairment. The Dementia Non-Communicable Diseases Risk Score (DemNCD) had the most missing variables (fruit and vegetable intake, loneliness, and cognitive activity). The Lifestyle for Brain Health Index (LIBRA) was validated without high cognitive activity, while both UK Biobank Dementia Risk Score UKBDRS and UKBDRS-APOE were missing parental history of dementia and whether participants lived alone.

The ANU-ADRI showed relatively stable external validation results, with its C-statistic for all-cause dementia (0.69, 95% CI: 0.63–0.76) aligning with its development range (0.65–0.73), though its performance for AD was somewhat lower (0.62, 95% CI: 0.51–0.72 vs. a development range of 0.64–0.74). Similarly, the BDSI experienced a moderate decline from its development range (0.68–0.78) to 0.63 (95% CI: 0.56–0.71), suggesting some attenuation in predictive accuracy. The DemNCD exhibited the largest deviation from its development estimates, with its C-statistic decreasing from 0.68 (95% CI: 0.65–0.70) in development to 0.42 (95% CI: 0.41–0.44). However, this discrepancy may, in part, reflect differences in follow-up duration between the development and validation datasets. The DemNCD model was developed using a median follow-up of 6.2 years. In contrast, the EPIC-Norfolk cohort had a median follow-up of 16.5 years. As a result, the model was applied in a context where risk accumulation over time may have differed substantially from the original development setting. Given that the model was constructed using a Fine-Gray approach, which accounts for competing risks, the longer follow-up in EPIC-Norfolk could have affected how well the model accounted for the balance between dementia incidence and the competing risk of death.

In contrast, similar performance was seen with the LIBRA score between the development (0.60, 95% CI: 0.53–0.67) and EPIC-Norfolk (0.61, 95% CI: 0.59–0.63), suggesting excellent transportability. The UKBDRS and UKBDRS-APOE models retained strong transportability, with external validation results (0.81, 95% CI: 0.79–0.82 and 0.82, 95% CI: 0.80–0.83, respectively) closely mirroring their development estimates (0.80, 95% CI: 0.78–0.82 and 0.83, 95% CI: 0.81–0.84)

Sex-stratified analyses showed varying degrees of consistency with the overall validation results, with most models exhibiting slightly higher discrimination in men compared to women (Table S2). The ANU-ADRI, which showed reasonable transportability in the total cohort, demonstrated stronger discrimination for all-cause dementia in men (C-statistic: 0.74, 95% CI: 0.66–0.82) compared to women (0.66, 95% CI: 0.56–0.75), with a similar pattern observed for Alzheimer’s disease (AD; 0.67 vs. 0.58). The BDSI performed comparably across sexes, with a C-statistic of 0.63 (95% CI: 0.52–0.73) in men and 0.65 (95% CI: 0.55–0.75) in women, suggesting relatively balanced performance. The DemNCD, which exhibited substantial degradation in overall transportability, performed notably worse in men (0.40, 95% CI: 0.37–0.43) compared to women (0.61, 95% CI: 0.59–0.63), reinforcing concerns about its generalizability across populations. Conversely, the LIBRA, which showed modest but stable performance in the total cohort, exhibited slightly higher discrimination in women (0.62, 95% CI: 0.59–0.64) than in men (0.59, 95% CI: 0.56–0.62). The UKBDRS and UKBDRS-APOE models, which demonstrated strong transportability overall, maintained high discrimination across sexes, with similar C-statistics in men (0.80–0.83) and women (0.81–0.85), suggesting robust generalizability. These findings highlight the potential need for sex-specific recalibration in models such as ANU-ADRI and DemNCD, where performance differences were most pronounced, while reaffirming the broad applicability of models such as UKBDRS across diverse populations.

The transportability of partially validated models varied across follow-up durations, with some models demonstrating relatively stable performance over time while others exhibited declines, particularly in sex-stratified analyses (Table S5). The ANU-ADRI (all-cause) showed a modest but consistent reduction in discriminative performance from 0.72 (95% CI: 0.63–0.81) at 5 years to 0.65 (95% CI: 0.64–0.67) at full follow-up, with a more pronounced decline in women (0.80 at 5 years to 0.65 at full follow-up) compared to men (0.66 at 5 years to 0.64 at full follow-up). A similar trend was observed for the BDSI, where discrimination fell from 0.69 (95% CI: 0.58–0.79) at 5 years to 0.60 (95% CI: 0.58–0.62) at full follow-up, with male performance dropping more sharply (0.72–0.58) than female performance (0.66–0.60). The LIBRA maintained relatively stable transportability, with a sharp decline from 0.71 at 5 years to 0.59 at full follow-up, showing better consistency in women (0.68–0.59) than men (0.74–0.58). The UKBDRS and UKBDRS-APOE demonstrated the strongest transportability among partially validated models, maintaining relatively stable C-statistics over time (0.80 at 5 years to 0.77 at full follow-up for UKBDRS; 0.80 to 0.77 for UKBDRS-APOE), with minimal differences between sexes.

The DemNCD model presented a distinct case, as it had the highest degree of missing predictors among partially validated models. Moreover, its original development and first external validation were conducted over the full follow-up period, while our secondary analyses examined performance at specific time intervals. Over the entire follow-up of this external validation, the DemNCD diverged significantly from its development estimate, however, when assessed at shorter follow-up durations using Cox regression, its discrimination improved, achieving 0.57 (95% CI: 0.47–0.68) at 5 years and 0.62 (95% CI: 0.57–0.67) at 10 years, before declining to 0.58 (95% CI: 0.56–0.59) at 20 years. Given the extent of missing predictors and differences in follow-up structure between the development and validation settings, it is difficult to disentangle the contribution of incomplete predictor availability from true lack of transportability. Accordingly, sex-stratified differences observed for DemNCD should be interpreted cautiously and are best viewed as descriptive rather than indicative of potential recalibration strategies. Notably, sex-stratified analyses revealed better discrimination in women (0.68 at 5 years to 0.61 at full follow-up) than men (0.61 at 5 years to 0.58 at full follow-up).

Fine-Gray analyses of the total sample across the different follow-up periods showed no significant deviations from the Cox regression results, except for the DemNCD, which showed lower discriminative performances when accounting for the competing risk of death (Table S6).

### Precision-based power analyses

A precision-based sample size assessment was undertaken to evaluate whether each external validation was adequately powered to estimate model performance metrics with confidence (Table S7). Among the full models, the CAIDE and CAIDE-APOE were adequately powered to estimate discrimination and calibration slope but lacked sufficient events to reliably estimate the O/E ratio. The FRS was adequately powered to estimate the C-statistic and slope, but underpowered for the O/E ratio. The DRS was only adequately powered to estimate the calibration slope, while the CHA₂DS₂-VASc was underpowered for all metrics due to extremely low event rates.

Among the partially validated models, performance assessment was restricted to discrimination. Of these, UKBDRS, UKBDRS-APOE, LIBRA, and DemNCD were all fully powered to estimate the C-statistic with precision. Notably, LIBRA, UKBDRS, and UKBDRS-APOE also met the event thresholds required to support calibration, had full predictor availability permitted it. In contrast, the ANU-ADRI models and BDSI were not adequately powered for precise estimation of discrimination or the O/E ratio.

## Discussion

This external validation study assessed the performance of 12 risk prediction models using <30 years of follow-up in a UK cohort. Findings suggest variability in model transportability, highlighting that some tools may be more readily applicable to real-world settings than others.

Among the fully validated models, the CAIDE and CAIDE-APOE exhibited moderate declines in discriminative performance relative to their development estimates, suggesting limited transportability. The CAIDE’s reliance on midlife vascular and lifestyle factors may explain its reduced transportability, as risk factor distributions can shift over time^9^, and additional unmeasured exposures in late life may influence dementia incidence beyond its original derivation. The DRS, which had the highest development C-statistic, exhibited a pronounced decline, particularly among women, suggesting that certain risk factor weightings may not have translated well across populations. Despite this, the DRS demonstrated strong transportability in multiple European-based validation studies, consistently achieving C-statistics above 0.70^10,11^. However, its decline in performance compared to other models indicates that recalibration is necessary.

The two CVD-based models exhibited strong transportability, suggesting that traditional cardiovascular risk factors may play a stable role in long-term dementia risk prediction^12,13^. Given the well-established links between vascular health and neurodegeneration^14^, the FRS’s ability to maintain strong discrimination across different follow-up periods and sex-stratified analyses supports its broader applicability beyond cardiovascular risk and stroke. Notably, its external validation in the Whitehall II cohort demonstrated moderate discriminative performance for all-cause dementia (C-statistic 0.72)^15^, supporting the notion that risk tools outside of dementia risk prediction may be repurposed. Similarly, the CHA₂DS₂-VASc, which was originally developed to predict stroke risk in patients with atrial fibrillation, demonstrated strong performance in dementia risk prediction. Notably, one study reported sex-specific differences in discrimination, with a C-statistic of 0.73 in men and 0.65 in women^16^, in contrast to recent recommendations for using CHA₂DS₂-Vasc without sex^17^. Collectively, the results imply that while vascular risk factors remain predictors of dementia in both sexes, they may contribute differently to dementia risk trajectories, potentially warranting sex-specific recalibration for optimal predictive accuracy. In interpreting model performance, it is important to recognise that in long-term dementia risk prediction, relatively modest differences in discrimination may still be informative in this context, particularly when supported by consistent calibration and stability across subgroups. Differences in C-statistics should therefore be considered alongside overall model coherence rather than in isolation^18^.

The partial validation revealed important insights into how missing variables affect model transportability and whether these models retain predictive performance despite incomplete data capture. The ANU-ADRI, despite lacking two psychosocial factors (social engagement and cognitive activity), exhibited reasonable transportability, with its C-statistic for all-cause dementia (0.69) aligning closely with its original development range (0.65–0.73). However, its performance for AD (0.62) was somewhat lower than in its development cohort. Given that psychosocial and cognitive stimulation factors have been linked to dementia risk^19^, their omission could lead to a slight overestimation of risk in individuals with high cognitive reserve. The ANU-ADRI demonstrated stronger discrimination in men than women. This gender imbalance may stem from sex-specific risk factor distributions or differential exposure to unmeasured psychosocial variables. Notably, the ANU-ADRI has exhibited transportability across diverse populations. In seven different low and middle-income country cohorts, it y exceeded its original discriminative performance (ranging from 0.66 to 0.78)^20^ and showed strong results in European settings such as the Netherlands (0.75)^11^. However, recent external validation revealed lower performance in the UK Biobank and Whitehall II cohorts (0.57 and 0.52 respectively)^10^.

The BDSI experienced a decline in discrimination, dropping from its development range (0.68–0.78) to 0.63. Since functional impairment is an early marker of cognitive decline^21^, its exclusion may have led to misclassification of individuals at higher dementia risk. Nevertheless, BDSI demonstrated comparable performance across sexes, with variations in discrimination, indicating that its core risk factors transported well. The DemNCD had the largest drop in discriminative performance which may be explained by the number of missing variables, which are all relevant predictors of dementia risk^22–24^. Furthermore, the DemNCD was developed with a median follow-up of 6.2 years, whereas the EPIC-Norfolk validation had a median follow-up of 16.5 years, meaning that the original model was applied in a vastly different time frame where risk accumulation differed. The Fine-Gray competing risk approach used in its development may also have contributed to its poor transportability, as the longer follow-up altered the balance between dementia incidence and competing risk of death. Despite this, shorter follow-up analyses showed improved discrimination, rising to 0.57 at 5 years and 0.62 at 10 years, before declining again at 20 years. This suggests that the DemNCD may have predictive value when applied over shorter time horizons but is less reliable for long-term forecasting. The LIBRA demonstrated exceptional stability despite lacking high cognitive activity. Its external validation (C-statistic: 0.61) slightly surpassed its development performance (0.60), indicating high transportability rather than superior discrimination. This transportability was further confirmed in the original CAIDE cohort, where LIBRA’s midlife discriminative performance exceeded its development values, even with missing variables like diet^25^.

The UKBDRS and UKBDRS-APOE models showed the strongest transportability among the partially validated models, with external validation C-statistics closely mirroring their development. These models were missing parental history of dementia and whether participants lived alone, two factors that are evidenced to influence dementia risk through genetic, social, and lifestyle mechanisms^26,27^. Despite this, both models retained excellent predictive performance, suggesting these variables may only marginally contribute or that their effects were adequately captured by other predictors. Furthermore, both models showed minimal sex differences, with similar discrimination in men and women. Given this high discrimination and stability over time, these models appear particularly robust in this cohort; however, further external validation in diverse populations remains necessary to confirm their generalisability.

In interpreting APOE-inclusive models, among ε4 homozygotes, who carry a substantially elevated baseline genetic risk, a considerable proportion of predicted risk is expected to reflect APOE itself. As this study aimed to validate models as originally specified, we did not attempt to disentangle the incremental contribution of non-genetic predictors within genotype strata. Nonetheless, the observed gradient in dementia incidence across ε4 copy number supports the coherent behaviour of APOE-inclusive models in genetically high-risk groups.

From a practical perspective, the observed levels of discrimination and calibration suggest that most validated models are better suited to risk stratification rather than population-wide screening. While several models, particularly UKBDRS, UKBDRS-APOE, FRS, and CHA₂DS₂-VASc, demonstrated strong discrimination and stable calibration, their performance does not yet support standalone use for unselected population screening, where very high specificity and clearly defined intervention thresholds would be required. Currently, these risk models may be most appropriately applied within research settings, targeted prevention programmes, or high-risk clinical populations, where relative risk ranking can inform further assessment or monitoring. Importantly, absolute risk estimates are sensitive to differences in baseline dementia incidence across cohorts and healthcare systems^28^. As demonstrated by the EPIC-Norfolk–specific recalibration, adjustment of the baseline hazard is likely to be necessary before applying these models in new settings. Recalibration should therefore be considered a prerequisite for clinical implementation to ensure accurate absolute risk estimation and avoid systematic over- or underprediction^29,30^.

These findings highlight the importance of transportability in dementia risk prediction and the need for ongoing external validation. Several partially validated models retained discrimination levels comparable to their original development, despite missing predictors, suggesting some omitted variables may have a limited impact. This supports the value of robust core predictors, particularly vascular and lifestyle factors, in maintaining model performance. Simpler models with fewer variables could remain clinically useful, easing implementation without sacrificing accuracy. Additionally, the strong performance of cardiovascular-based models reinforces their potential for repurposing in dementia risk prediction. Integrating such models could strengthen prevention strategies by targeting modifiable vascular risk factors^31^.

The sex differences in discrimination imply that sex-specific recalibration is necessary to improve accuracy in risk estimation, as evidenced in risk stratification in CVD^32^. These disparities may stem from differences in baseline vascular risk burden, hormonal influences, or survival biases that impact the predictive validity of certain risk factors over time^33,34^. Future work should explore sex-specific model adjustments, either through re-weighting risk factor contributions or including sex-specific predictors.

Our precision-based power analysis revealed that many of the models were underpowered, particularly for estimating calibration metrics. This is a critical consideration, as calibration is often more sensitive to limited event counts than discrimination measures^35^. Dementia is often underdiagnosed, particularly in early stages^36^, which may have led to further underestimation of events and compounded power limitations. Notably, several partial validations (UKBDRS, UKBDRS-APOE, LIBRA) had sufficient sample sizes and event counts for full calibration but were limited by unavailable predictors. This underscores the importance of considering power not just in model development but also in external validation planning^37,38^. While precision-based methods provide a valuable framework for assessing performance, interpretation must reflect real-world challenges such as under-ascertainment. Strict adherence to idealised thresholds may undervalue informative findings. Future validation efforts should integrate precision-based planning while accounting for diagnostic limitations and the uncertainty inherent in rare outcomes like dementia.

The EPIC-Norfolk cohort is a large, well-characterised, population-representative sample with up to 30 years of follow-up, allowing for a long-term validation of dementia risk models. The use of linked electronic health records and validated ICD-coded diagnoses enhances diagnostic accuracy, while the integration of self-reported data captures a broader range of risk factors. The study systematically assessed model performance at multiple follow-up periods including both Cox and Fine-Gray competing risk models, providing insights into how discrimination evolves over time and under different statistical frameworks. Additionally, sex-stratified analyses revealed important differences in predictive accuracy. Finally, this is one of the first external validation studies in the area to evaluate statistical power. Dementia ascertainment relied on routine healthcare records, which may be subject to diagnostic misclassification or delayed diagnosis, potentially attenuating discrimination and influencing calibration, particularly at longer follow-up durations. The reliance on health record linkage may under-ascertainment dementia cases, particularly for individuals diagnosed outside of linked registries or with undiagnosed dementia. In addition, several validations were underpowered. Lastly, as EPIC-Norfolk consists predominantly of White European participants, further validation in international cohorts is needed.

This study revealed variability in the transportability of dementia risk prediction models, with implications for clinical implementation. While some models like UKBDRS and cardiovascular-based tools demonstrated robust performance across populations, others showed limitations that necessitate recalibration before widespread adoption. The consistent finding of sex-specific differences in model discrimination underscores the need for tailored risk assessment approaches. Furthermore, the stability of certain models despite missing variables suggests that streamlined prediction tools focusing on core vascular and lifestyle factors may offer utility while minimising data-collection burden. Our precision-based evaluation also highlights the importance of adequate sample-size and event rates in future validation efforts. These findings provide guidance for selecting and refining risk prediction tools that can identify at-risk individuals and inform targeted interventions.

## Methods

### Data source and reporting guidelines

In brief, the EPIC-Norfolk study includes 30,437 participants aged 39 to 79 years, registered at participating general practices, who were recruited between 1993 and 1997 from the region surrounding Norwich (Norfolk, UK). Of these, 25,636 participants completed the baseline health check and were included in this analysis. Linked electronic health records from National Health Service (NHS) Digital, including Hospital Episode Statistics (HES), and mortality data from the Office for National Statistics (ONS) up to March 2023 were also available. The EPIC-Norfolk study was approved by the Norwich Local Research Ethics Committee with all participants providing signed informed consent.

The current study followed reporting guidelines of the Transparent Reporting of a multivariable prediction model for Individual Prognosis or Diagnosis (TRIPOD + AI)^29^ and Strengthening the Reporting of Observational Studies in Epidemiology (STROBE) statement for cohort studies^39^ (Tables S8 and S9).

### Selection of dementia risk prediction models

Informed by a series of systematic reviews previously conducted by the authors^2,6,40^ and based on recent publications, we selected 12 risk prediction models for validation (Table S10), prioritising models based on either their prior use in external validation studies, availability of predictor variables in the EPIC-Norfolk dataset, or use of routinely collected or clinically relevant variables to support real-world implementation. Selected risk models included the ANU-ADRI^41^, BDSI^42^, CAIDE^43^, the extended version of CAIDE, incorporating APOE genotype (CAIDE-APOE)^43^, DemNCD^44^, DRS^45^, LIBRA^46,47^, UKBDRS, and the extended version of UKBDRS incorporating APOE genotype (UKBDRS-APOE)^10^. In addition to risk models that were developed to predict dementia as an outcome, we evaluated two cardiovascular-based risk models, including the CHA₂DS₂-VASc score^48^ and FRS^49^.

### Dementia ascertainment

Dementia outcomes were ascertained through linked electronic health records and mortality data, using International Classification of Diseases, 10th Revision (ICD-10) codes as defined by Hayat and colleagues^50^ (see Table S11 for all ICD codes used). The outcomes of interest were all-cause dementia and AD. All-cause dementia included any recorded diagnosis of dementia, irrespective of subtype, including vascular dementia and mixed dementia diagnoses. Alzheimer’s disease was defined using specific ICD-10 codes. Where mixed dementia diagnoses were recorded, these cases were retained within the all-cause dementia outcome and were not classified as AD. Separate analyses of vascular or other dementia subtypes were not undertaken in order to maintain direct alignment with the original development outcomes of the evaluated models. Participants diagnosed with dementia during or before the baseline health check were excluded, in addition to those diagnosed with young-onset dementia (i.e., <65 years). Follow-up for participants commenced from the date of their baseline health check and continued until the earliest occurrence of either dementia diagnosis, death, or conclusion of the follow-up in March 2023.

### Statistical analysis

All statistical analyses were performed using Stata version 18.5. Discriminative performance was assessed via C-statistics (with corresponding 95% CI), which measures the ability of each model to distinguish individuals who develop dementia from those who do not. First, all risk models were externally validated according to their development parameters, including statistical methodology, dementia outcome, age eligibility, follow-up duration, and predictor variables (see Table S12 for variable mapping). Model calibration was assessed by comparing predicted and observed dementia risks across deciles of predicted probabilities. For logistic regression-based models, CITL was evaluated by comparing mean predicted and observed risks, and calibration slopes were estimated by regressing observed outcomes on predicted log-odds. The Hosmer-Lemeshow (HL) test was performed to assess goodness-of-fit by reporting the chi-square (χ^2^) statistic and *p*-value. For Cox-based models, predicted survival probabilities were computed using the baseline survival function, and observed risks were derived from Kaplan-Meier estimates. The Grønnesby and Borgan (GB) test was used to evaluate model fit by reporting the χ^2^ statistic and *p*-value. Calibration plots with 95% CIs were generated to visually inspect agreement with the 45-degree reference line. Recalibration, if required, was performed using logistic recalibration, adjusting the intercept and slope. Recalibration decisions were based on CITL, calibration slopes, model fit tests, and graphical assessment of calibration plots. Only fully validated models (i.e., those with all necessary predictor variables available) were calibrated, while models missing key predictors were assessed for discrimination only.

To further examine differences in predictive accuracy across life stages, each model was evaluated at follow-ups of 5, 10, 15, 20, 25 years and full follow-up time (up to a maximum of 30 years), starting at the age specified at model development. In addition, all analyses were stratified by sex to explore potential differences in discriminative performance. For this, Cox proportional hazards regression was used across all models to account for time-to-event data and censoring (e.g., death or dropout). Recognising the importance of competing risks (particularly death in late-life prediction), we performed further sensitivity analyses using Fine-Gray competing-risks models, as this approach mitigates the risk of overestimating dementia incidence when deaths are treated solely as censored events^11,51^. Calibration of all fully validated models was also assessed at each timepoint using calibration plots comparing predicted versus observed dementia probabilities. These were complemented by residual plots, which displayed the smoothed difference between observed and predicted probabilities across the risk spectrum and follow-up durations. All analyses were conducted under a complete-case scenario, where only participants with complete data for all variables in the respective models were included.

### Precision-based power analysis

We further assessed whether our main external validation samples were sufficient to estimate model performance metrics with acceptable precision, following recent methodological guidance on precision-based sample size calculation for prediction model validation by Riley and colleagues^37,38^. Using the ‘pmvalsampsize’ command in Stata, we calculated the minimum required sample size and number of events to estimate the observed-to-expected (O/E) ratio, calibration slope, and C-statistic with prespecified CI widths of 0.1, 0.2, and 0, respectively. For each model, we provided the observed event rate, the C-statistic from our validation data, and assumed a normal distribution for the linear predictor when required.

### Ethical approval

The EPIC-Norfolk study was approved by the Norwich Local Research Ethics Committee with all participants providing signed informed consent, following the principles of the Declaration of Helsinki and the Research Governance Framework for Health and Social Care.

## Supplementary information

44400_2026_95_MOESM1_ESM

## Data Availability

Data are available upon reasonable request from the EPIC-Norfolk study team and subject to approval by the EPIC data access committee. Analysis code is available from the corresponding author upon reasonable request.
