# Supplementary material for: External validation of dementia risk prediction models in the EPIC-Norfolk cohort: a UK-based cohort study
Source: NPJ Dement. 2026 Jun 19;2(1):44. doi: 10.1038/s44400-026-00095-7 (PMC13282172; doi:10.1038/s44400-026-00095-7)
Supplement: Supplementary file 1 — 44400_2026_95_MOESM1_ESM [file 44400_2026_95_MOESM1_ESM.pdf]

Supplementary Tables

Table S1: Dementia incidence and APOE-inclusive risk score distributions by APOE ε4 copy number
Table S2: Sex-stratified external validation results
Table S3: Calibration results
Table S4: Recalibration results
Table S5: Time-varying Cox regression results
Table S6: Fine and Gray sensitivity analysis
Table S7: Precision-based power analysis
Table S8: TRIPOD+AI checklist
Table S9: STROBE checklist
Table S10: Overview of included models
Table S11: ICD codes
Table S12: Model variables mapped to the EPIC-Cohort data

Supplementary Figures

Figure S1: Participant flowchart
Figure S2: Participant follow-up status over time
Figure S3: CAIDE calibration plots
Figure S4: CAIDE-APOE calibration plots
Figure S5: CAIDE (female) recalibration plot
Figure S6: DRS calibration plots
Figure S7: CHA_2DS_2-VASc calibration plots
Figure S8: FRS calibration plots
Figure S9: FRS (male) recalibration plot
Figure S10: Time-dependant Cox regression calibration and residual plots

Supplementary Tables

Table S1: Dementia incidence and APOE-inclusive risk score distributions by APOE ε4 copy number

APOE ε4 copy number	N	Dementia cases n (%)	Absolute risk difference vs non-carrier (95% CI)	CAIDE-APOE median (IQR)	UKBDRS-APOE median (IQR)
0 (non-carrier)	14,931	1,548 (10.4%)	Reference	8 (6–10)	72.3 (64.7–80.0)
1 (heterozygous)	5,422	1,033 (19.1%)	+8.7% (7.5–9.8)	10 (8–12)	73.2 (65.7–80.9)
2 (homozygous)	513	161 (31.4%)	+21.0% (17.0–25.1)	10 (8–12)	73.4 (65.7–80.8)

Note: Absolute risk differences calculated using ε4 non-carriers as the reference group. Restricted to participants with available APOE genotype data (n = 20,866).

Table S2: Sex-stratified external validation results using development parameters

Model	Dementia outcome	Men			Women		
		n/N (death)	C-statistic	Calibration	n/N (death)	C-statistic	Calibration
ANU-ADRI	All-cause	5,217/26	0.74 (0.66-0.82)	N/A	5,687/32	0.66 (0.56-0.75)	N/A
ANU-ADRI	AD	5,217/8	0.67 (0.54-0.79)	N/A	5,687/15	0.58 (0.44-0.72)	N/A
BDSI	All-cause	3,838/29	0.63 (0.52-0.73)	N/A	4,238/35	0.65 (0.55-0.75)	N/A
CAIDE	All-cause	5,649/62	0.73 (0.67-0.78)	8.48 (0.205)	7,483/107	0.66 (0.61-0.71)	17.83 (0.013)
CAIDE-APOE	All-cause	4,996/53	0.76 (0.70-0.81)	8.30 (0.307)	6,406/91	0.69 (0.64-0.75)	13.03 (0.111)
DemNCD	All-cause	2,823/554 (2,075)	0.40 (0.37-0.43)	N/A	2,772/804 (1,628)	0.61 (0.59-0.63)	N/A
DRS	All-cause	5,554/13	0.83 (0.76-0.90)	6.76 (0.662)	6,121/25	0.72 (0.61-0.82)	6.10 (0.730)
LIBRA	All-cause	8,076/303	0.59 (0.56-0.62)	N/A	9,904/471	0.62 (0.59-0.64)	N/A
UKBDRS	All-cause	9,220/245 (2,220)	0.80 (0.78-0.83)	N/A	10,753/341 (1,567)	0.81 (0.80-0.83)	N/A
UKBDRS-APOE	All-cause	7,828/202 (1,773)	0.80 (0.78-0.83)	N/A	8,616/250 (1,148)	0.83 (0.81-0.85)	N/A
CHA2DS2-Vasc	All-cause	10,868/10	0.83 (0.73-0.92)	2.54 (0.282)	13,456/18	0.78 (0.68-0.89)	0.78 (0.678)
FRS	All-cause	9,852/73	0.82 (0.79-0.86)	10.45 (0.315)	12,061/77	0.78 (0.73-0.82)	8.73 (0.463)

Table S3. Full model calibration results

Model	Total					Male					Female				
	χ^2 (p-value)	Mean predicted risk	Observed risk	CITL	Slope	χ^2 (p-value)	Mean predicted risk	Observed risk	CITL	Slope	χ^2 (p-value)	Mean predicted risk	Observed risk	CITL	Slope
CAIDE	12.26 (0.092)	0.0134	0.0129	0.0000	1.0000	8.48 (0.205)	0.0117	0.0110	0.0000	1.0000	17.83 (0.013)	0.0148	0.0143	0.0000	1.0000
CAIDE-APOE	5.15 (0.642)	0.0133	0.0126	0.0000	1.0000	8.30 (0.307)	0.0114	0.0106	0.0000	1.0000	13.03 (0.111)	0.0147	0.0142	0.0000	1.0000
DRS	11.232 (0.260)	0.0035	0.0033	-0.0002	0.9701	6.76 (0.662)	0.0027	0.0023	-0.0003	0.9542	6.10 (0.730)	0.0043	0.0041	-0.0002	0.9765
CHA2DS2-Vasc	1.49 (0.684)	0.0012	0.0012	0.0000	1.0000	2.54 (0.282)	0.0011	0.0009	0.0000	1.0000	0.78 (0.678)	0.0014	0.0013	0.0000	1.0000
FRS	12.41(0.191)	0.0079	0.0068	-0.0010	0.9165	10.45 (0.315)	0.0090	0.0074	-0.0016	0.8910	8.73 (0.463)	0.0070	0.0064	-0.0006	0.9494

Table S4: Full model recalibration results

Recalibrated model	Recalibrated intercept	Recalibrated slope	Sample size	Observed 10-year risk	Mean predicted risk (original)	Mean predicted risk (recalibrated)
CAIDE (Female)	0.00	1.00	7,483	0.0148	0.0148	0.0148
FRS (Male)	-8.96	0.89	9,852	0.0074	0.0090	0.0074

Table S5: Time-varying Cox regression results

Prediction model	C-statistic (95% CI)																	
	5-year			10-year			15-year			20-year			25-year			Full follow-up		
	Total	Male	Female	Total	Male	Female	Total	Male	Female	Total	Male	Female	Total	Male	Female	Total	Male	Female
ANU-ADRI n/N	10,904/36	5,217/13	5,687/23	10,904/193	5,217/91	5,687/102	10,904/627	5,217/262	5,687/365	10,904/1,337	5,217/552	5,687/785	10,904/2,172	5,217/866	5,687/1,306	10,904/2,477 (29.80)	5,217/962	5,687/1,515
ANU-ADRI	0.72 (0.63-0.81)	0.80 (0.66-0.94)	0.66 (0.55-0.78)	0.70 (0.66-0.74)	0.70 (0.65-0.75)	0.71 (0.66-0.76)	0.69 (0.67-0.71)	0.69 (0.66-0.72)	0.69 (0.67-0.72)	0.68 (0.66-0.70)	0.67 (0.65-0.69)	0.69 (0.67-0.71)	0.66 (0.64-0.68)	0.65 (0.63-0.67)	0.67 (0.65-0.68)	0.65 (0.64-0.67)	0.64 (0.63-0.66)	0.66 (0.65-0.68)
BDSI n/N	8,076/37	3,838/14	4,238/23	8,076/214	3,838/93	4,238/121	8,076/627	3,838/252	4,238/375	8,076/1,242	3,838/483	4,238/759	8,076/1,810	3,838/675	4,238/1,135	8,076/1,972 (29.8)	3,838/718 (29.5)	4,238/1,254 (29.8)
BDSI	0.69 (0.58-0.79)	0.72 (0.56-0.87)	0.66 (0.53-0.79)	0.62 (0.58-0.66)	0.60 (0.55-0.66)	0.64 (0.59-0.69)	0.60 (0.58-0.62)	0.58 (0.55-0.62)	0.61 (0.58-0.64)	0.60 (0.58-0.62)	0.58 (0.55-0.60)	0.62 (0.60-0.64)	0.59 (0.58-0.61)	0.58 (0.55-0.60)	0.60 (0.58-0.62)	0.59 (0.58-0.61)	0.58 (0.55-0.60)	0.60 (0.58-0.62)
CAIDE n/N	16,167/3	7,239/0	8,928/3	16,167/20	7,239/10	8,928/10	16,167/93	7,239/42	8,928/51	16,167/364	7,239/166	8,928/198	16,167/971	7,239/447	8,928/524	16,167/1,318 (30)	7,239/571	8,928/747
CAIDE	0.64 (0.48-0.81)	N/A	0.67 (0.52-0.82)	0.67 (0.58-0.77)	0.70 (0.53-0.86)	0.67 (0.55-0.78)	0.68 (0.64-0.73)	0.66 (0.59-0.73)	0.70 (0.65-0.76)	0.67 (0.64-0.70)	0.69 (0.65-0.73)	0.66 (0.63-0.70)	0.67 (0.66-0.69)	0.68 (0.66-0.70)	0.66 (0.64-0.69)	0.67 (0.65-0.68)	0.67 (0.65-0.69)	0.67 (0.65-0.68)
CAIDE-APOE n/N	13,947/3	6,363/0	7,584/3	13,947/17	6,363/9	7,584/8	13,947/79	6,363/36	7,584/43	13,947/307	6,363/143	7,584/164	13,947/818	6,363/391	7,584/427	13,947/1,116 (30)	6,363/506 (30)	7,584/610 (30)
CAIDE-APOE	0.79 (0.66-0.91)	N/A	0.80 (0.69-0.91)	0.70 (0.59-0.81)	0.70 (0.50-0.91)	0.71 (0.60-0.82)	0.72 (0.67-0.77)	0.70 (0.63-0.78)	0.73 (0.68-0.78)	0.70 (0.67-0.73)	0.72 (0.69-0.76)	0.69 (0.66-0.72)	0.70 (0.68-0.71)	0.70 (0.68-0.72)	0.69 (0.67-0.71)	0.69 (0.68-0.71)	0.69 (0.67-0.72)	0.69 (0.67-0.71)
DemNCD n/N	5,595/24	2,823/9	2,772/15	5,595/131	2,823/66	2,772/65	5,595/422	2,823/182	2,772/240	5,595/845	2,823/357	2,772/488	5,595/1,239	2,823/517	2,772/722	5,595/1,358	2,823/554	2,772/804
DemNCD	0.57 (0.47-0.68)	0.61 (0.44-0.78)	0.68 (0.56-0.80)	0.62 (0.57-0.67)	0.62 (0.55-0.69)	0.66 (0.59-0.72)	0.59 (0.56-0.62)	0.62 (0.58-0.66)	0.63 (0.60-0.67)	0.58 (0.57-0.60)	0.61 (0.58-0.64)	0.63 (0.60-0.65)	0.58 (0.56-0.59)	0.60 (0.58-0.63)	0.61 (0.59-0.63)	0.58 (0.56-0.59)	0.60 (0.57-0.63)	0.61 (0.59-0.63)

DRS n/N	11,675/38	5,554/13	6,121/25	11,675/214	5,554/95	6,121/119	11,675/676	5,554/277	6,121/399	11,675/1,436	5,554/583	6,121/853	11,675/2,314	5,554/905	6,121/1,409	11,675/2,637	5,554/1,006	6,121/1,631 (30)
DRS	0.76 (0.69-0.83)	0.83 (0.76-0.90)	0.72 (0.61-0.82)	0.75 (0.72-0.78)	0.73 (0.69-0.78)	0.76 (0.72-0.80)	0.73 (0.71-0.75)	0.72 (0.69-0.75)	0.74 (0.72-0.76)	0.71 (0.69-0.72)	0.70 (0.68-0.72)	0.71 (0.70-0.73)	0.69 (0.67-0.70)	0.68 (0.66-0.70)	0.69 (0.67-0.70)	0.68 (0.67-0.69)	0.68 (0.66-0.70)	0.68 (0.67-0.70)
LIBRA n/N	17,980/32	8,076/10	9,904/22	17,980/188	8,076/82	9,904/106	17,980/630	8,076/246	9,904/384	17,980/1,438	8,076/558	9,904/880	17,980/2,480	8,076/960	9,904/1,520	17,980/2,952	8,076/1,113	9,904/1,839
LIBRA	0.71 (0.62-0.80)	0.74 (0.55-0.93)	0.68 (0.59-0.78)	0.62 (0.59-0.66)	0.63 (0.57-0.69)	0.62 (0.57-0.67)	0.61 (0.59-0.63)	0.59 (0.56-0.63)	0.62 (0.60-0.65)	0.60 (0.58-0.61)	0.58 (0.56-0.60)	0.61 (0.59-0.63)	0.59 (0.58-0.60)	0.58 (0.56-0.60)	0.60 (0.59-0.62)	0.59 (0.58-0.60)	0.58 (0.56-0.59)	0.59 (0.58-0.61)
UKB DRS n/N	19,973/40	9,220/15	10,753/25	19,973/234	9,220/104	10,753/130	19,973/724	9,220/297	10,753/427	19,973/1,625	9,220/656	10,753/969	19,973/2,778	9,220/1,109	10,753/1,669	19,973/3,282	9,220/1,272	10,753/2,010
UKB DRS	0.80 (0.76-0.84)	0.83 (0.75-0.90)	0.80 (0.75-0.85)	0.81 (0.79-0.84)	0.82 (0.79-0.85)	0.82 (0.79-0.84)	0.81 (0.79-0.82)	0.81 (0.79-0.83)	0.81 (0.79-0.83)	0.78 (0.77-0.79)	0.78 (0.77-0.80)	0.79 (0.77-0.80)	0.77 (0.76-0.77)	0.76 (0.75-0.78)	0.77 (0.76-0.78)	0.76 (0.75-0.77)	0.76 (0.75-0.77)	0.77 (0.76-0.78)
UKB DRS-APOE n/N	16,444/30	7,828/11	8,616/19	16,444/176	7,828/83	8,616/93	16,444/565	7,828/243	8,616/322	16,444/1,321	7,828/552	8,616/769	16,444/2,274	7,828/951	8,616/1,323	16,444/2,676	7,828/1,096	8,616/1,580
UKB DRS-APOE	0.80 (0.75 - 0.85)	0.83 (0.73 - 0.94)	0.80 (0.75 - 0.85)	0.82 (0.80 - 0.85)	0.82 (0.78 - 0.86)	0.83 (0.80 - 0.86)	0.81 (0.80 - 0.83)	0.81 (0.79 - 0.83)	0.82 (0.80 - 0.84)	0.79 (0.78 - 0.80)	0.79 (0.77 - 0.81)	0.79 (0.78 - 0.81)	0.77 (0.76 - 0.78)	0.77 (0.75 - 0.78)	0.78 (0.77 - 0.79)	0.77 (0.76 - 0.78)	0.76 (0.75 - 0.78)	0.77 (0.76 - 0.78)
CHA_2DS_2-VASc n/N	25,365/41	11,497/15	13,868/26	25,365/237	11,497/104	13,868/133	25,365/729	11,497/299	13,868/430	25,365/1,645	11,497/666	13,868/979	25,365/2,838	11,497/1,140	13,868/1,698	25,365/3,732	11,497/1,315	13,868/2,057
CHA_2DS_2-VASc	0.81 (0.74 - 0.87)	0.80 (0.72 - 0.89)	0.82 (0.75 - 0.89)	0.78 (0.75 - 0.81)	0.79 (0.76 - 0.83)	0.82 (0.79 - 0.85)	0.76 (0.75 - 0.78)	0.77 (0.75 - 0.80)	0.80 (0.78 - 0.82)	0.74 (0.73 - 0.75)	0.75 (0.74 - 0.77)	0.77 (0.76 - 0.78)	0.72 (0.71 - 0.73)	0.73 (0.71 - 0.74)	0.75 (0.74 - 0.76)	0.71 (0.70 - 0.72)	0.72 (0.71 - 0.73)	0.74 (0.73 - 0.75)
FRS n/N	21,913/27	9,852/11	12,061/16	21,913/150	9,852/73	12,061/77	21,913/548	9,852/233	12,061/315	21,913/1,296	9,852/539	12,061/757	21,913/2,351	9,852/966	12,061/1,385	21,913/2,830	9,852/1,126	12,061/1,704
FRS	0.78 (0.71 - 0.84)	0.76 (0.62 - 0.90)	0.80 (0.74 - 0.87)	0.79 (0.76 - 0.82)	0.82 (0.79 - 0.86)	0.78 (0.73 - 0.82)	0.79 (0.77 - 0.80)	0.80 (0.78 - 0.83)	0.79 (0.77 - 0.81)	0.77 (0.75 - 0.78)	0.78 (0.76 - 0.79)	0.77 (0.76 - 0.79)	0.75 (0.75 - 0.76)	0.76 (0.75 - 0.78)	0.76 (0.75 - 0.77)	0.75 (0.74 - 0.76)	0.76 (0.74 - 0.77)	0.76 (0.75 - 0.77)

Table S6: Fine and Gray sensitivity analysis

	5 years		10 years		15 years		20 years		25 years		Full follow-up	
	n/N (death)	C-statistic (95%CI)	n/N (death)	C-statistic (95%CI)	n/N (death)	C-statistic (95%CI)	n/N (death)	C-statistic (95%CI)	n/N (death)	C-statistic (95%CI)	n/N (death)	C-statistic (95%CI)
ANU-ADRI	10,904/36 (754)	0.72 (0.63-0.81)	10,904/193 (1,856)	0.70 (0.66-0.74)	10,904/627 (3,200)	0.69 (0.67-0.71)	10,904/1,337 (4,630)	0.68 (0.66-0.69)	10,904/2,172 (5,944)	0.66 (0.65-0.67)	10,904/2,477 (6,464)	0.65 (0.64-0.67)
BDSI	8,076/37 (702)	0.69 (0.58-0.79)	8,076/214 (1,696)	0.62 (0.58-0.66)	8,076/627 (2,862)	0.60 (0.58-0.62)	8,076/1,242 (4,072)	0.60 (0.58-0.62)	8,076/1,810 (5,057)	0.59 (0.58-0.61)	8,076/1,972 (5,368)	0.59 (0.58-0.61)
CAIDE	15,418/1 (249)	0.54 (0.53-0.54)	15,418/14 (662)	0.69 (0.58-0.81)	15,418/73 (1,247)	0.69 (0.64-0.74)	15,418/307 (1,990)	0.67 (0.64-0.69)	15,418/830 (3,022)	0.67 (0.65-0.68)	15,418/1,148 (3,671)	0.66 (0.65-0.68)
CAIDE-APOE	13,305/1 (185)	0.71 (0.70-0.72)	13,305/11 (527)	0.70 (0.56-0.85)	13,305/62 (1,028)	0.71 (0.65-0.77)	13,305/261 (1,679)	0.70 (0.67-0.73)	13,305/703 (2,577)	0.70 (0.68-0.71)	13,305/976 (3,107)	0.69 (0.68-0.71)
DemNCD	5,595/24 (473)	0.57 (0.47-0.68)	5,595/131 (1,139)	0.62 (0.57-0.67)	5,595/422 (1,958)	0.59 (0.56-0.62)	5,595/845 (2,801)	0.58 (0.57-0.60)	5,595/1,239 (3,489)	0.42 (0.41-0.44)	5,595/1,358 (3,703)	0.42 (0.41-0.44)
DRS	11,675/38 (809)	0.76 (0.69-0.83)	11,675/214 (1,986)	0.75 (0.72-0.78)	11,675/676 (3,426)	0.73 (0.71-0.75)	11,675/1,436 (4,962)	0.71 (0.69-0.72)	11,675/2,314 (6,382)	0.69 (0.67-0.70)	11,675/2,637 (6,943)	0.68 (0.67-0.69)
LIBRA	17,980/32 (741)	0.71 (0.62-0.80)	17,980/188 (1,928)	0.62 (0.59-0.66)	17,980/630 (3,433)	0.61 (0.59-0.63)	17,980/1,438 (5,150)	0.60 (0.58-0.61)	17,980/2,480 (6,966)	0.59 (0.58-0.60)	17,980/2,952 (7,851)	0.59 (0.58-0.60)
UKBDRS	19,973/40 (970)	0.80 (0.76-0.84)	19,973/234 (2,411)	0.81 (0.79-0.84)	19,973/724 (4,187)	0.81 (0.80-0.82)	19,973/1,625 (6,149)	0.78 (0.77-0.79)	19,973/2,778 (8,159)	0.77 (0.76-0.77)	19,973/3,282 (9,118)	0.76 (0.75-0.77)
UKBDRS-APOE	16,444/30 (654)	0.80 (0.75, 0.85)	16,444/176 (1,792)	0.83 (0.81, 0.85)	16,444/565 (3,253)	0.82 (0.80, 0.83)	16,444/1,321 (4,872)	0.79 (0.78, 0.81)	16,444/2,274 (6,558)	0.77 (0.76, 0.79)	16,444/2,676 (7,325)	0.77 (0.76, 0.78)
CHA_2DS_2-VASc	25,365/41 (1,007)	0.81 (0.74-0.87)	25,365/237 (2,521)	0.78 (0.75-0.81)	25,365/729 (4,385)	0.76 (0.75-0.78)	25,365/1,645 (6,490)	0.74 (0.73-0.75)	25,365/2,838 (8,695)	0.72 (0.71-0.73)	25,365/3,372 (9,772)	0.71 (0.70-0.72)
FRS	21,913/27 (773)	0.78 (0.71-0.85)	21,913/150 (1,944)	0.79 (0.76-0.82)	21,913/548 (3,435)	0.79 (0.77-0.80)	21,913/1,296 (5,166)	0.77 (0.75-0.78)	21,913/2,351 (7,053)	0.75 (0.75-0.76)	21,913/2,830 (7,987)	0.75 (0.74-0.76)

Table S7: Precision-based power analysis

		Actual					Required				Adequately powered?
Model	Follow-up (years)	n/N	Event rate (%)	C-statistic (95%CI)	Calibration slope	CITL	N (for C-statistic)	N (for slope)	N (O/E)	Complete n/N	
ANU-ADRI	6	10,904/58	0.53	0.69 (0.63-0.76)	N/A	N/A	20,106	3,314	72,157	72,157/383	**Partial**: Not adequately powered for C-statistic and O/E ratio.
ANU-ADRI	6	10,904/23	0.21	0.62 (0.51-0.72)	N/A	N/A	56,846	3,314	182,696	182,696/384	**Partial**: Not adequately powered for C-statistic and O/E ratio.
BDSI	6	8,076/64	0.79	0.63 (0.56-0.71)	N/A	N/A	15,005	3,314	48,283	48,283/382	**Partial**: Not adequately powered for C-statistic and O/E ratio.
CAIDE	20	13,132/169	1.29	0.68 (0.64-0.72)	0.0000	1.0000	8,499	3,314	29,420	29,420/380	**Partial:** Adequately powered for estimating the c-statistic and calibration slope, but not for O/E.
CAIDE-APOE	20	11,402/144	1.26	0.71 (0.67-0.75)	0.0000	1.0000	8,140	3,314	30,129	30,129/380	**Partial:** Sufficiently powered for estimating the c-statistic and slope, but underpowered for O/E.
DemNCD	10	5,595/1,358	24.27	0.42 (0.41-0.44)	N/A	N/A	677	2,257	1,200	2,257/548	**Fully powered**
DRS	5	11,675/38	0.33	0.76 (0.69-0.83)	-0.0002	0.9701	26,647	3,314	116,121	116,121/384	**Partial**: Adequately powered for

											slope only; underpowered for C-statistic and O/E due to low events.
LIBRA	16	17,980/774	4.30	0.61 (0.59-0.63)	N/A	N/A	2,931	2,774	8,557	8,557/368	**Fully powered**
UKBDRS	14	19,973/586	2.93	0.81 (0.79-0.82)	N/A	N/A	2,526	2,774	12,738	12,738/374	**Fully powered**
UKBDRS-APOE	14	16,444/452	2.75	0.82 (0.80-0.83)	N/A	N/A	2,559	2,774	13,597	13,597/374	**Fully powered**
CHA_2DS_2-Vasc	5	24,324/28	0.12	0.80 (0.72-0.87)	0.0000	1.0000	62,681	4,151	320,006	320,006/385	**Partial**: Not adequately powered for C-statistic and O/E ratio.
FRS	10	21,913/150	0.68	0.79 (0.76-0.82)	-0.0010	0.9165	11,607	3,314	56,155	56,155/382	**Partial**: Adequate for C-statistic and slope but not O/E.

Table S8: TRIPOD+AI checklist [1]

Section/Topic	Item	Development / evaluation[1]	Checklist item	Reported on page
**TITLE**				
Title	1	D;E	Identify the study as developing or evaluating the performance of a multivariable prediction model, the target population, and the outcome to be predicted	1
**ABSTRACT**				
Abstract	2	D;E	See TRIPOD+AI for Abstracts checklist	2
**INTRODUCTION**				
Background	3a	D;E	Explain the healthcare context (including whether diagnostic or prognostic) and rationale for developing or evaluating the prediction model, including references to existing models	4/5
	3b	D;E	Describe the target population and the intended purpose of the prediction model in the context of the care pathway, including its intended users (e.g., healthcare professionals, patients, public)	4/5
	3c	D;E	Describe any known health inequalities between sociodemographic groups	N/A
Objectives	4	D;E	Specify the study objectives, including whether the study describes the development or validation of a prediction model (or both)	5
**METHODS**				
Data	5a	D;E	Describe the sources of data separately for the development and evaluation datasets (e.g., randomised trial, cohort, routine care or registry data), the rationale for using these data, and representativeness of the data	4/5
	5b	D;E	Specify the dates of the collected participant data, including start and end of participant accrual; and, if applicable, end of follow-up	4-6
Participants	6a	D;E	Specify key elements of the study setting (e.g., primary care, secondary care, general population) including the number and location of centres	4/5
	6b	D;E	Describe the eligibility criteria for study participants	5/6, Supp
	6c	D;E	Give details of any treatments received, and how they were handled during model development or evaluation, if relevant	N/A
Data preparation	7	D;E	Describe any data pre-processing and quality checking, including whether this was similar across relevant sociodemographic groups	4-6
Outcome	8a	D;E	Clearly define the outcome that is being predicted and the time horizon, including how and when assessed, the rationale for choosing this outcome, and whether the method of outcome assessment is consistent across sociodemographic groups	5-7
	8b	D;E	If outcome assessment requires subjective interpretation, describe the qualifications and demographic characteristics of the outcome assessors	N/A
	8c	D;E	Report any actions to blind assessment of the outcome to be predicted	N/A
Predictors	9a	D	Describe the choice of initial predictors (e.g., literature, previous models, all available predictors) and any pre-selection of predictors before model building	N/A
	9b	D;E	Clearly define all predictors, including how and when they were measured (and any actions to blind assessment of predictors for the outcome and other predictors)	Supp
	9c	D;E	If predictor measurement requires subjective interpretation, describe the qualifications and demographic characteristics of the predictor assessors	Supp
Sample size	10	D;E	Explain how the study size was arrived at (separately for development and evaluation), and justify that the study size was sufficient to answer the research question. Include details of any sample size calculation	7/8, Supp

Missing data	11	D;E	Describe how missing data were handled. Provide reasons for omitting any data	6
Analytical methods	12a	D	Describe how the data were used (e.g., for development and evaluation of model performance) in the analysis, including whether the data were partitioned, considering any sample size requirements	N/A
	12b	D	Depending on the type of model, describe how predictors were handled in the analyses (functional form, rescaling, transformation, or any standardisation).	N/A
	12c	D	Specify the type of model, rationale[2], all model-building steps, including any hyperparameter tuning, and method for internal validation	N/A
	12d	D;E	Describe if and how any heterogeneity in estimates of model parameter values and model performance was handled and quantified across clusters (e.g., hospitals, countries). See TRIPOD-Cluster for additional considerations[3]	N/A
	12e	D;E	Specify all measures and plots used (and their rationale) to evaluate model performance (e.g., discrimination, calibration, clinical utility) and, if relevant, to compare multiple models	8-10
	12f	E	Describe any model updating (e.g., recalibration) arising from the model evaluation, either overall or for particular sociodemographic groups or settings	9
	12g	E	For model evaluation, describe how the model predictions were calculated (e.g., formula, code, object, application programming interface)	Supp
Class imbalance	13	D;E	If class imbalance methods were used, state why and how this was done, and any subsequent methods to recalibrate the model or the model predictions	9
Fairness	14	D;E	Describe any approaches that were used to address model fairness and their rationale	N/A
Model output	15	D	Specify the output of the prediction model (e.g., probabilities, classification). Provide details and rationale for any classification and how the thresholds were identified	N/A
Training versus evaluation	16	D;E	Identify any differences between the development and evaluation data in healthcare setting, eligibility criteria, outcome, and predictors	Table 2, Supp
Ethical approval	17	D;E	Name the institutional research board or ethics committee that approved the study and describe the participant-informed consent or the ethics committee waiver of informed consent	32-33
**OPEN SCIENCE**				
Funding	18a	D;E	Give the source of funding and the role of the funders for the present study	33
Conflicts of interest	18b	D;E	Declare any conflicts of interest and financial disclosures for all authors	33
Protocol	18c	D;E	Indicate where the study protocol can be accessed or state that a protocol was not prepared	N/A
Registration	18d	D;E	Provide registration information for the study, including register name and registration number, or state that the study was not registered	N/A
Data sharing	18e	D;E	Provide details of the availability of the study data	33
Code sharing	18f	D;E	Provide details of the availability of the analytical code[4]	33
**PATIENT & PUBLIC INVOLVEMENT**				
Patient & Public Involvement	19	D;E	Provide details of any patient and public involvement during the design, conduct, reporting, interpretation, or dissemination of the study or state no involvement.	N/A
**RESULTS**				
Participants	20a	D;E	Describe the flow of participants through the study, including the number of participants with and without the outcome and, if applicable, a summary of the follow-up time. A diagram may be helpful.	Supp
	20b	D;E	Report the characteristics overall and, where applicable, for each data source or setting, including the key dates, key predictors (including demographics), treatments received, sample size, number of outcome events, follow-up time, and amount of missing data. A table may be helpful. Report any differences across key demographic groups.	11-12, Table 1
	20c	E	For model evaluation, show a comparison with the development data of the distribution of important predictors (demographics, predictors, and outcome).	N/A

Model development	21	D;E	Specify the number of participants and outcome events in each analysis (e.g., for model development, hyperparameter tuning, model evaluation)	Table 2, Supp
Model specification	22	D	Provide details of the full prediction model (e.g., formula, code, object, application programming interface) to allow predictions in new individuals and to enable third-party evaluation and implementation, including any restrictions to access or re-use (e.g., freely available, proprietary)[5]	N/A
Model performance	23a	D;E	Report model performance estimates with confidence intervals, including for any key subgroups (e.g., sociodemographic). Consider plots to aid presentation.	11-21, Supp
	23b	D;E	If examined, report results of any heterogeneity in model performance across clusters. See TRIPOD Cluster for additional details[3].	N/A
Model updating	24	E	Report the results from any model updating, including the updated model and subsequent performance	Supp
**DISCUSSION**				
Interpretation	25	D;E	Give an overall interpretation of the main results, including issues of fairness in the context of the objectives and previous studies	22-32
Limitations	26	D;E	Discuss any limitations of the study (such as a non-representative sample, sample size, overfitting, missing data) and their effects on any biases, statistical uncertainty, and generalizability	30-31
Usability of the model in the context of current care	27a	D	Describe how poor quality or unavailable input data (e.g., predictor values) should be assessed and handled when implementing the prediction model	N/A
	27b	D	Specify whether users will be required to interact in the handling of the input data or use of the model, and what level of expertise is required of users	N/A
	27c	D;E	Discuss any next steps for future research, with a specific view to applicability and generalizability of the model	22-32

Table S9: STROBE checklist [2]

	Item No	Recommendation
**Title and abstract**	1	(*a*) Indicate the study's design with a commonly used term in the title or the abstract
		(*b*) Provide in the abstract an informative and balanced summary of what was done and what was found
**Introduction**		
Background/rationale	2	Explain the scientific background and rationale for the investigation being reported
Objectives	3	State specific objectives, including any prespecified hypotheses
**Methods**		
Study design	4	Present key elements of study design early in the paper
Setting	5	Describe the setting, locations, and relevant dates, including periods of recruitment, exposure, follow-up, and data collection
Participants	6	(*a*) Give the eligibility criteria, and the sources and methods of selection of participants. Describe methods of follow-up
		(*b*) For matched studies, give matching criteria and number of exposed and unexposed
Variables	7	Clearly define all outcomes, exposures, predictors, potential confounders, and effect modifiers. Give diagnostic criteria, if applicable
Data sources/ measurement	8*	For each variable of interest, give sources of data and details of methods of assessment (measurement). Describe comparability of assessment methods if there is more than one group
Bias	9	Describe any efforts to address potential sources of bias
Study size	10	Explain how the study size was arrived at
Quantitative variables	11	Explain how quantitative variables were handled in the analyses. If applicable, describe which groupings were chosen and why
Statistical methods	12	(*a*) Describe all statistical methods, including those used to control for confounding
		(*b*) Describe any methods used to examine subgroups and interactions
		(*c*) Explain how missing data were addressed
		(*d*) If applicable, explain how loss to follow-up was addressed
		(*e*) Describe any sensitivity analyses
**Results**		
Participants	13*	(a) Report numbers of individuals at each stage of study—eg numbers potentially eligible, examined for eligibility, confirmed eligible, included in the study, completing follow-up, and analysed
		(b) Give reasons for non-participation at each stage
		(c) Consider use of a flow diagram
Descriptive data	14*	(a) Give characteristics of study participants (eg demographic, clinical, social) and information on exposures and potential confounders
		(b) Indicate number of participants with missing data for each variable of interest
		(c) Summarise follow-up time (eg, average and total amount)
Outcome data	15*	Report numbers of outcome events or summary measures over time

Main results	16	(*a*) Give unadjusted estimates and, if applicable, confounder-adjusted estimates and their precision (eg, 95% confidence interval). Make clear which confounders were adjusted for and why they were included
		(*b*) Report category boundaries when continuous variables were categorized
		(*c*) If relevant, consider translating estimates of relative risk into absolute risk for a meaningful time period
Other analyses	17	Report other analyses done—eg analyses of subgroups and interactions, and sensitivity analyses
**Discussion**		
Key results	18	Summarise key results with reference to study objectives
Limitations	19	Discuss limitations of the study, taking into account sources of potential bias or imprecision. Discuss both direction and magnitude of any potential bias
Interpretation	20	Give a cautious overall interpretation of results considering objectives, limitations, multiplicity of analyses, results from similar studies, and other relevant evidence
Generalisability	21	Discuss the generalisability (external validity) of the study results
**Other information**		
Funding	22	Give the source of funding and the role of the funders for the present study and, if applicable, for the original study on which the present article is based

Table S10: Overview of included risk models

Model	Development/first validation cohort(s)	Age (years)	Follow-up (years)	Overview
The Australian National University AD Risk Index (ANU-ADRI)[3]	CVHS, MAP, KP	≥60	6	The ANU-ADRI was developed using a literature-derived risk modelling framework to estimate risk of AD and all-cause dementia based on modifiable and demographic risk factors. Rather than being derived from a single cohort, risk factors were identified through systematic review, and pooled effect estimates from meta-analyses and high-quality prospective studies were used to generate standardized beta coefficients. These were converted into an additive, points-based risk score designed for self-report administration. The tool was subsequently externally validated in three independent longitudinal cohorts (MAP, KP, and CVHS).
Brief dementia screening index (BDSI)[4]	CVHS, FHS, HRS, SALSA	≥60	6	The BDSI was derived from pooled data across four US-based longitudinal cohorts, targeting a pragmatic set of dementia risk factors suitable for routine clinical use. The model uses Cox regression and includes variables selected for their strong predictive value and ease of assessment in older adults.
Cardiovascular Risk Factors, Aging, and Incidence of Dementia (CAIDE)[5]	CAIDE cohort	39-64	20	The CAIDE risk score was developed from the North Karelia and FINMONICA studies in Finland, where participants were followed from midlife to late life to identify predictors of dementia onset. The model incorporates vascular and lifestyle risk factors measured in midlife that were statistically associated with dementia incidence after 20 years. Predictors were weighted using logistic regression, producing a simple, additive score that can be applied to individuals in midlife to support early risk identification and prevention.
DemNCD[6]	ARIC, CHS, FHS, CFAS-I/II, MAS, MAAS, HRS ADAMS, MAP, SLAS-I	≥65	Mean = 23.5	The DemNCD model was developed using pooled data from 10 population-based cohort studies across multiple continents, comprising a combined sample of 41,755 participants aged ≥65 years. The model was designed to estimate risk for several non-communicable diseases, including dementia, stroke, diabetes, and

				myocardial infarction. Predictor selection followed a structured, multi-stage process informed by systematic reviews, international guidelines, and expert ranking, resulting in 22 candidate sociodemographic, medical, and lifestyle variables. All selected predictors were retained in the final model to support comprehensive risk assessment. Sex-specific Fine and Gray sub-distribution hazard models were used to account for the competing risk of death, with coefficients combined across cohorts using meta-analysis and subsequently converted into a weighted point-based risk score. A logistic regression–based version of the tool (DemNCD-LR) was also developed as a sensitivity analysis but was not used in the present study.
Dementia Risk Score (DRS)[7]	THIN	60-79	5	The DRS is a Cox-based regression model developed using data from THIN, a large UK primary care database. The development cohort included 930,395 patients aged 60 to 95 years across 377 general practices, all free of dementia at baseline. Separate models were derived for two age groups (60–79 and 80–95 years) to account for differences in risk profiles. In the present study, the 60–79 age group model was used to align with the EPIC-Norfolk cohort, where participants did not exceed 79 years at baseline.
LIfestyle for BRAin health risk score (LIBRA)[8]	MAAS	≥50	16	The LIBRA score was designed to estimate dementia risk using a composite of modifiable lifestyle and health-related factors. It was informed by a systematic review of observational studies and initially validated in the Maastricht Aging Study (MAAS), a longitudinal cohort of 949 individuals aged 50 to 81 years with up to 16 years of follow-up. The index incorporates 11 risk and protective factors, each weighted according to effect sizes derived from meta-analyses of their associations with cognitive decline and dementia.
UKBioBank Dementia Risk Score (UKBDRS)[9]	UKBiobank and Whitehall II	≥50	14	The UKBDRS was developed using data from the UK Biobank, a large-scale prospective cohort of over 500,000 individuals aged 40–73 at baseline. The model was designed to estimate 14-year risk of incident dementia and was externally validated in the Whitehall II

				study. Development involved applying LASSO Cox regression to a set of 28 candidate variables, with final model estimation performed using Fine-Gray regression to account for non-dementia mortality. There is also an addition model that includes APOE, the UKBDRS-APOE.
CHA2DS2-Vasc[10]	Euro Heart Survey	≥18	5	Originally developed to estimate stroke risk in individuals with atrial fibrillation, the CHA_2DS_2-VASc score has since been evaluated for its relevance in dementia prediction. The model was derived using data from large-scale atrial fibrillation cohorts, including the Danish National Patient Registry and other European datasets and uses logistic regression. It assigns weighted points to common vascular risk factors, such as age, sex, hypertension, diabetes, heart failure, and prior stroke, all of which also influence dementia risk. Although not designed specifically for cognitive outcomes, the score's applicability to dementia has been supported by evidence linking cardiovascular burden to neurodegenerative decline.
Framingham Risk Score (FRS) [11]	FHS	30-74	10	The Framingham Risk Score was created to estimate 10-year cardiovascular disease risk using data from the Framingham Heart Study, a landmark U.S. cohort initiated in 1948. The model includes predictors such as age, sex, smoking, systolic blood pressure, cholesterol levels, and diabetes status and uses Cox proportionate hazard regression. While originally intended for cardiovascular endpoints, the FRS has been repurposed for dementia risk prediction given the well-established association between midlife vascular risk and later-life cognitive decline.

Abbreviations: Apolipoprotein E (APOE); Atherosclerosis Risk in Communities (ARIC); Alzheimer's disease (AD); Cardiovascular Health Cognition Study (CVHS); Cognitive Function and Ageing Studies I and II (CFAS-I and CFAS-II); Framingham Heart Study (FHS); Health and Retirement Study-Aging, Demographics, and Memory Study (HRS ADAMS); Logistic regression (LR); The Health Improvement Network database (THIN); Kungsholmen Project (KP); Maastricht Aging Study (MAAS); Non-communicable disease (NCD); Rush Memory and Aging Study (MAP); Singapore Longitudinal Ageing Study-I (SLAS-I); The Health Improvement Network database (THIN); Sydney Memory and Aging Study (MAS); UK Biobank (UKB)

Table S11: ICD codes

	**ICD-9 codes**	**ICD-10 codes**
All-cause dementia	N/A	A81.0, F02, F02.0-F024, F02.8, F03, F04, F05.1, F10.7, G31.0, G31.8, I67.3, F00, F00.0-F00.2, F00.9, G30, G30.0, G30.1, G30.8, G30.9, F01, F01.0-F01.3, F01.8, F01.9
Alzheimer's disease	N/A	F00, F00.0-F00.2, F00.9, G30, G30.0, G30.1, G30.8, G30.9
Diabetes	250	E10-E14
Traumatic brain injury	850-854, 800-804	S02, S06, S07.0, S07.1, S07.8, S07.9, S09.7-S09.9
Renal failure	584-586	N17-N19
Vascular disease	4139, 4109, 4129, 4119, 4140, 4141, 4148, 4149, 4275, 440-448	I20.0, I20.1,I20.8, I20.9, I21.0, I21.1, I21.2, I21.3, I21.4, I21.9, I22.0, I22.1, I22.8, I22.9, I23.0, I23.1, I23.2, I23.3, I23.5, I23.6, I23.8, I24.0, I24.1, I24.8, I24.9, I25.0, I25.1, I25.2, I25.3, I25.4, I25.5, I25.6, I25.8, I25.9, I46.0, I46.1, I46.9, I70-I79
Atrial fibrillation	427.3	I48
Coronary heart disease (including codes ischaemic heart disease, myocardial infarction, and angina pectoris)	410, 4139, 4109, 4129, 4119, 4140, 4141, 4148, 4149, 4275	I20.0, I20.1,I20.8, I20.9, I21.0, I21.1, I21.2, I21.3, I21.4, I21.9, I22.0, I22.1, I22.8, I22.9, I23.0, I23.1, I23.2, I23.3, I23.5, I23.6, I23.8, I24.0, I24.1, I24.8, I24.9, I25.0, I25.1, I25.2, I25.3, I25.4, I25.5, I25.6, I25.8, I25.9, I46.0, I46.1, I46.9
Stroke	430-439	I60-I69

Table S12: Model variables mapped to the EPIC-Cohort data

Model	Original predictor	Mapped EPIC-Norfolk variables	Assigned score	
			Male	Female
ANU-ADRI [3]	Age	Age at first health check		
	< 65 years	< 65 years	0	0
	65 - 70 years	65 - 70 years	1	5
	70 - 75 years	70 - 75 years	12	14
	75 - 80 years	75 - 80 years	18	21
	80 - 85 years	80 - 85 years	26	29
	85 - 90 years	85 - 90 years	33	35
	≥ 90 years	≥ 90 years	38	41
	Education	Highest education attainment		
	> 11 years	A level/degree	0	
	8 - 11 years	O level	3	
	< 8 years	No formal qualification	6	
	Diabetes	Diabetes		
	No	No	0	
	Yes	Yes (self-reported, linked hospital data, or >6.5% HbA1c)	3	
	TBI	TBI		
	No	No	0	
	Yes	Yes (Linked hospital data)	4	
	Depressive symptoms	Depression		
	CES-D < 16	No	0	
	CES-D ≥ 16	Yes (self-reported depression (requiring treatment) or anti-depressant use)	2	
	Smoking	Smoking		
	Never	Never	0	
	Former	Former	1	
	Current	Current	4	
	Alcohol intake	Alcohol intake		
	None	None/teetotaller	0	
	Light/moderate	< 14 units per week	-3	
	Heavy	≥ 14 units per week	3	
	Social engagement	Excluded		
	Highest		0	
	Medium to high		1	
	Low to medium		4	
	Lowest		6	
	Physical activity	Physical activity		
	Low	Inactive/moderately inactive	0	
	Medium	Moderately active	-2	

	High	Active	-3
	BMI<br>< 25 kg/m^2<br>≥ 25 to <30 kg/m^2<br>≥ 30 kg/m^2	BMI<br>< 25 kg/m^2<br>≥ 25 to <30 kg/m^2<br>≥ 30 kg/m^2	 0<br>2<br>5
	Total cholesterol<br>< 6.2mmol/L or ≥ 60 years old<br>≥ 6.2mmol/L	Total cholesterol<br>< 6.2mmol/L or ≥ 60 years old<br>≥ 6.2mmol/L	 0<br>3
	Fish intake*<br>< 0.25 portions per week<br>0.25-2 portions per week<br>2-4 portions per week<br>≥ 4 portions per week	Fish intake*<br>< 0.25 portions per week or missing data<br>0.25-2 portions per week<br>2-4 portions per week<br>≥ 4 portions per week	 0<br>-3<br>-4<br>-5
	Cognitive activity<br>Low<br>Moderate<br>High	Excluded	 0<br>-6<br>-7
	Pesticide exposure<br>Never exposed<br>Exposed	Pesticide exposure<br>None<br>Self-reported worked in arable farming using herbicides/pesticides in past year	 0<br>2
**BDSI [4]**	Age<br><66<br>66-79	Age<br><66<br>66-79	 0<br>1 per year
	Education<br>≥ 12 years<br>< 12 years	Highest education attainment<br>A Level/degree<br>O levels or no formal qualifications	 0<br>9
	BMI<br>≥ 18.5 kg/m2<br>< 18.5 kg/m2	BMI<br>≥ 18.5 kg/m2<br>< 18.5 kg/m2	 0<br>8
	Diabetes<br>Absent<br>Present	Diabetes<br>No<br>Yes (self-reported diabetes, linked hospital data, or >6.5% HbA1c)	 0<br>8
	History of stroke<br>No<br>Yes	Stroke<br>No<br>Self-reported or linked hospital data	 0<br>6
	Functional impairment<br>No help needed money/medication<br>Needs help with money/medication	Functional impairment<br>Excluded due to lack of data	 0<br>10
	Depressive symptoms	Depression	

	No<br>Yes (CES-D or antidepressant use)	No<br>Yes (Self-reported depression (requiring treatment) or anti-depressant use)	0<br>6
**CAIDE [5]**	Age<br>< 47 years<br>47-53 years<br>> 53 years	Age<br>< 47 years<br>47-53 years<br>> 53 years	 0<br>3<br>4
	Education<br>≥10 years<br>7-9 years<br>0-6 years	Highest education attainment<br>Completed A Levels/degree<br>Completed O levels<br>No formal qualifications	 0<br>2<br>3
	Sex<br>Women<br>Men	Sex<br>Women<br>Men	 0<br>1
	SBP<br>< 140 mm Hg<br>≥ 140 mm Hg	SBP<br>< 140 mm Hg<br>≥ 140 mm Hg	 0<br>2
	BMI<br>< 30kg/m^2<br>≥ 30kg/m^2	BMI<br>< 30kg/m^2<br>≥ 30kg/m^2	 0<br>2
	Total cholesterol<br>< 6.5mmol/L<br>≥ 6.5mmol/L	Total cholesterol<br>< 6.5mmol/L<br>≥ 6.5mmol/L	 0<br>2
	Physical activity<br>Active<br>Inactive	Physical activity<br>Moderately active/active<br>Inactive/moderately inactive or missing data	 0<br>1
**CAIDE (APOE) [5]**	Age<br>< 47 years<br>47-53 years<br>> 53 years	Age<br>< 47 years<br>47-53 years<br>> 53 years	 0<br>3<br>5
	Education<br>≥10 years<br>7-9 years<br>0-6 years	Education<br>Completed A Levels/degree<br>Completed O levels<br>No formal qualifications	 0<br>3<br>4
	Sex<br>Women<br>Men	Sex<br>Women<br>Men	 0<br>1
	SBP<br>< 140 mm Hg<br>≥ 140 mm Hg	SBP<br>< 140 mm Hg<br>≥ 140 mm Hg	 0<br>2
	BMI	BMI	

	< 30kg/m^2	< 30kg/m^2	0	
	≥ 30kg/m^2	≥ 30kg/m^2	2	
	Total cholesterol	Total cholesterol		
	< 6.5mmol/L	< 6.5mmol/L	0	
	≥ 6.5mmol/L	≥ 6.5mmol/L	1	
	Physical activity	Physical activity		
	Active	Moderately active/active	0	
	Inactive	Inactive/moderately inactive	1	
	APOE e4 status	APOE e4 status		
	Non-carrier	Non-carrier	0	
	Carrier	Carrier	2	
**DemNCD [6]**	Age	Age	Male	Female
	65 – 69	65 – 69	-8	-9
	70 – 74	70 – 74	0	0
	75 – 79	75 – 79	11	8
	80 – 84	80 – 84	15	14
	85 – 89	85 – 89	25	24
	90+	90+	49	32
	Education	Highest education attainment	Male	Female
	Less than secondary	No qualification/O-Level	7	3
	Upper secondary	A-level	4	2
	Tertiary	Degree	0	0
	Obesity	Obesity	Male	Female
	Underweight (BMI <18.5)	Underweight (BMI <18.5)	11	2
	Normal weight (BMI 18.5 – 24.9)	Normal weight (BMI 18.5 – 24.9)	0	0
	Overweight (BMI 25 - 29.9)	Overweight (BMI 25 - 29.9)	0	-3
	Obese (BMI ≥30)	Obese (BMI ≥30)	-1	-6
	Alcohol consumption	Alcohol consumption	Male	Female
	Low (none)	Low (none)	0	0
	Moderate (<14 units drinks per week)	Moderate (<14 units drinks per week)	-5	-6
	High (≥14 units drinks per week)	High (≥14 units drinks per week)	-1	-9
	Smoking	Smoking	Male	Female
	Non-smoker	Non-smoker	0	0
	Current smoker	Current smoker	-3	-2
	Former smoker	Former smoker	0	4
	Hypertension	Hypertension	Male	Female
	Yes (systolic blood pressure ≥ 140 or diastolic ≥90 or history of hypertension)	Yes (self-reported, SBP ≥ 140 mm Hg, DBP ≥ 90 mm Hg, or antihypertensive use)	0	-1
	High cholesterol	High cholesterol	Male	Female
	Yes (total cholesterol >6.2 mmol/L)	Yes (total cholesterol >6.2 mmol/L)	4	5
			Male	Female

	Low HDL<br>Yes (HDL <1.0 mmol/L for male and <1.3 mmol/L for female)	Low HDL<br>Yes (HDL <1.0 mmol/L for male and <1.3 mmol/L for female)	3	-3
	High LDL<br>Yes (LDL >4.1 mmol/L)	High LDL<br>Yes (LDL >4.1 mmol/L)	Male<br>2	Female<br>-5
	Depression<br>Yes (self-reported)	Depression<br>Yes (self-reported depression (requiring treatment) or anti-depressant use)	Male<br>-2	Female<br>4
	Fish consumption<br>≥2 servings per week*	Fish consumption<br>≥2 servings per week*	Male<br>2	Female<br>0
	Fruit and vegetable consumption<br>Yes (5 servings of vegetables or 3 servings of vegetables and 2 fruits daily)	Excluded	Male<br>0	Female<br>-4
	TBI<br>Yes (self-reported)	TBI<br>Yes (linked hospital data)	Male<br>0	Female<br>-1
	Loneliness<br>Yes (self-reported)	Excluded	Male<br>4	Female<br>9
	Physical activity<br>Inactive	Physical activity<br>Inactive/moderately inactive	Male<br>6	Female<br>2
	Cognitive activity<br>Low<br>Moderate<br>High	Excluded	Male<br>0<br>1<br>-13	Female<br>0<br>1<br>-8
	Atrial fibrillation<br>Yes (self-reported or ECG data)	Atrial fibrillation<br>Yes (self-reported arrhythmia or linked hospital data)	Male<br>3	Female<br>2
	Sleep problem<br>Yes (self-reported trouble falling asleep, waking up too early/late, never has good sleep)	Sleep problem<br>Yes (self-reported insomnia requiring treatment)	Male<br>0	Female<br>-5
	Hearing loss<br>Yes (self-reported, recommended a hearing aid)	Hearing loss<br>Yes (self-reported hearing problems)	Male<br>4	Female<br>0
	Diabetes<br>Yes (self-reported, fasting/not fasting blood glucose level, clinical diagnosis)	Diabetes<br>Yes (self-reported, linked hospital data, or >6.5% HbA1c)	Male<br>0	Female<br>1
	Stroke<br>Yes (self-reported or clinical diagnosis)	Stroke<br>Yes (self-reported or linked hospital data)	Male<br>13	Female<br>8
	Myocardial infarction<br>Yes (self-reported or clinical diagnosis)	Myocardial infarction<br>Yes (self-reported or linked hospital data)	Male<br>-4	Female<br>-2
**DRS (60-79) [7]**	Age<br>Years	Age<br>Years	Continuous	

	Sex<br>Men<br>Women	Sex<br>Men<br>Women	 0<br>1
	Calander year<br>Year at baseline	Calander year<br>Year at baseline	 Continuous
	Deprivation quintile<br>Per quintile	Deprivation quintile<br>Per quintile	 1
	BMI<br>kg/m^2	BMI<br>kg/m^2	 Continuous
	Current antihypertensive use<br>No<br>Yes	Current antihypertensive use<br>No<br>Yes	 0<br>1
	Smoking status<br>None/former<br>Current	Smoking status<br>None/former<br>Current	 0<br>1
	History of alcohol problem<br>< 56/49 units per week (men/women)<br>≥ 56/49 units per week (men/women) or Read-code entry of alcohol problem	History of alcohol problem<br>< 56/49 units per week (men/women)<br>≥ 56/49 units per week (men/women)	 0<br>1
	Diabetes<br>No<br>Yes	Diabetes<br>No<br>Yes (self-reported, linked hospital data, or >6.5% HbA1c)	 0<br>1
	Current depression/antidepressant use<br>No<br>Yes	Current depression/antidepressant use<br>No<br>Self-reported depression (requiring treatment) or anti-depressant use	 0<br>1
	History of stroke/TIA<br>No<br>Yes	History of stroke/TIA<br>No<br>Self-reported or linked hospital data	 0<br>1
	History of atrial fibrillation<br>No<br>Yes	History of atrial fibrillation<br>No<br>Self-reported or linked hospital data	 0<br>1
	Current aspirin use<br>No<br>Yes	Current aspirin use<br>No<br>Yes (self-reported)	 0<br>1
**LIBRA [8]**	Mediterranean diet<br>High adherence	Mediterranean diet<br>Top tertile**	 -1.7
	Physical inactivity<br>Lowest tertile	Physical inactivity<br>Inactive/moderately inactive	 +1.1
	Smoking<br>Current smoker	Smoking<br>Current smoker	 +1.5

	<u>Alcohol consumption</u><br>< 14 units per week	<u>Alcohol consumption</u><br>< 14 units per week	-1.0
	<u>Obesity</u><br>BMI ≥ 30	<u>Obesity</u><br>BMI ≥ 30	+1.6
	<u>Hypertension</u><br>≥ 140mmHg SBP/ ≥ 90mmHg DBP/antihypertensive medication use	<u>Hypertension</u><br>≥ 140mmHg SBP/ ≥ 90mmHg DBP/antihypertensive medication use	+1.6
	<u>High cholesterol</u><br>Self-reported cholesterol-lowering medication use or high cholesterol	<u>High cholesterol</u><br>Self-reported statin use or ≥6.2 mmol/L total cholesterol	+1.4
	<u>Depression</u><br>Top tertile of Symptom Checklist 90 subscale	<u>Depression</u><br>Self-reported depression (requiring treatment) or anti-depressant use	+2.1
	<u>Diabetes</u><br>Self-reported diabetes	<u>Diabetes</u><br>Self-reported diabetes, linked hospital data, or ≥6.5% HBA1c	+1.3
	<u>CHD</u><br>Self-reported heart/vascular disease	<u>CHD</u><br>Self-reported myocardial infarction/angina, or linked hospital data	+1
	<u>Renal dysfunction</u><br>Self-reported renal disease	<u>Renal failure</u><br>Linked hospital data	+1.1
	<u>High cognitive activity</u><br>Self-reported average hours per week reading or doing mental exercises	Excluded	-3.2
**UKBDRS [9]**	<u>Age</u>	<u>Age</u>	Continuous
	<u>Parental history of dementia</u><br>No<br>Yes	Excluded	0<br>1
	<u>Education</u><br>Years	<u>Highest education attainment</u><br>Highest qualification mapped to approximate years of education in-line with the UK schooling system	Continuous
	<u>Townsend deprivation</u><br>Not most deprived<br>Most deprived	<u>Townsend deprivation</u><br>Not most deprived<br>Most deprived	0<br>1
	<u>Diabetes (Type I and II)</u><br>No<br>Self-reported diabetes or primary care data	<u>Diabetes (Type I and II)</u><br>No<br>Self-reported diabetes, linked hospital data, or ≥6.5% HBA1c	0<br>1
	<u>Depression</u><br>No<br>Self-reported current/historical depression and ICD-9/10 codes	<u>Depression</u><br>No<br>Self-reported depression (requiring treatment) or anti-depressant use	0<br>1
	<u>Stroke</u><br>No<br>Self-reported stroke/TIA, or ICD-9/10 codes	<u>Stroke</u><br>No<br>Self-reported stroke/TIA, or linked hospital data	0<br>1

	<u>Hypertensive</u><br>No<br>Yes (self-reported hypertension, antihypertensive use, or ICD-10 codes)	<u>Hypertensive</u><br>No<br>Yes (self-reported, SBP ≥ 140 mm Hg, DBP ≥ 90 mm Hg, or antihypertensive use)	 0<br>1
	<u>High cholesterol</u><br>No<br>Self-reported high cholesterol or statin use, or ICD-10 codes	<u>High cholesterol</u><br>No<br>Self-reported statin use, or total cholesterol ≥6.2 mmol/L	 0<br>1
	<u>Sex</u><br>Women<br>Men	<u>Sex</u><br>Women<br>Men	 0<br>1
	<u>Lives alone</u><br>No<br>Yes	Excluded	 0<br>1
**UKBDRS (APOE) [9]**	<u>Age</u>	<u>Age</u>	Continuous
	<u>Parental history of dementia</u><br>No<br>Yes	<u>Parental history of dementia</u><br>Excluded	 0<br>1
	<u>Education</u><br>Years	<u>Highest education attainment</u><br>Highest qualification mapped to approximate years of education in-line with the UK schooling system	Continuous
	<u>Townsend deprivation</u><br>Not most deprived<br>Most deprived	<u>Townsend deprivation</u><br>Not most deprived<br>Most deprived	 0<br>1
	<u>Diabetes (Type I and II)</u><br>No<br>Self-reported diabetes or primary care data	<u>Diabetes (Type I and II)</u><br>No<br>Self-reported diabetes, linked hospital data, or ≥6.5% HBA1c	 0<br>1
	<u>Depression</u><br>No<br>Self-reported current/historical depression and ICD-9/10 codes	<u>Depression</u><br>No<br>Self-reported depression (requiring treatment) or anti-depressant use	 0<br>1
	<u>Stroke</u><br>No<br>Self-reported stroke/TIA, or ICD-9/10 codes	<u>Stroke</u><br>No<br>Self-reported stroke/TIA, or linked hospital data	 0<br>1
	<u>Hypertensive</u><br>No<br>Yes (Self-reported hypertension or antihypertensive use, or ICD-10 codes)	<u>Hypertensive</u><br>No<br>Yes (self-reported, SBP ≥ 140 mm Hg, DBP ≥ 90 mm Hg, or antihypertensive use)	 0<br>1
	<u>High cholesterol</u><br>No<br>Self-reported high cholesterol or statin use, or ICD-10 codes	<u>High cholesterol</u><br>No<br>Self-reported statin use, or total cholesterol ≥6.2 mmol/L	 0<br>1
	<u>Sex</u><br>Women<br>Men	<u>Sex</u><br>Women<br>Men	 0<br>1
	<u>Lives alone</u><br>No<br>Yes	Excluded	 0<br>1

	<u>APOE e4 genotype</u><br>No<br>One or more e4 alleles	<u>APOE e4 genotype</u><br>No<br>One or more e4 alleles	 0<br>1	
**CHA_2DS_2-VASc [10]**	<u>Age</u><br>< 65<br>65-74<br>≥ 75	<u>Age</u><br>< 65<br>65-74<br>≥ 75	 0<br>1<br>2	
	<u>Hypertension</u><br>No (< 140mm Hg SBP/ ≥ 90mm Hg DBP)<br>Yes (≥ 140mm Hg SBP/ ≥ 90mm Hg DBP/antihypertensive medication use)	<u>Hypertension</u><br>No (< 140mm Hg SBP, ≥ 90mm Hg DBP)<br>Yes (≥ 140mm Hg SBP, ≥ 90mm Hg DBP, antihypertensive medication, or self-reported hypertension)	 0<br>1	
	<u>Congestive heart failure</u><br>No<br>Signs/symptoms of heart failure or objective evidence of reduced left-ventricular ejection fraction	<u>Heart failure</u><br>No<br>Linked hospital data on incident heart failure	 0<br>1	
	<u>Diabetes</u><br>No<br>Fasting glucose >125 mg/dL (7 mmol/L) or oral hypoglycaemic agent and/or insulin	<u>Diabetes</u><br>No<br>Yes (self-reported, linked hospital data, or >6.5% HbA1c)	 0<br>1	
	<u>Stroke/TIA/thromboembolism</u><br>No<br>Yes	<u>Stroke/TIA/thromboembolism</u><br>No<br>Yes (self-report or linked hospital data)	 0<br>2	
	<u>Vascular disease</u><br>None<br>Previous myocardial infarction, peripheral artery disease, or aortic plaque	<u>Vascular disease</u><br>None<br>Self-reported myocardial infarction or linked hospital data	 0<br>1	
	<u>Sex</u><br>Male<br>Female	<u>Sex</u><br>Male<br>Female	 0<br>1	
**FRS [11]**	<u>Age</u>	<u>Age</u>	Male	Female
	30 – 34<br>35 – 39<br>40 – 44<br>45 – 49<br>50 – 54<br>55 – 59<br>60 – 64<br>65 – 69<br>70 – 74<br>≥ 75	30 – 34<br>35 – 39<br>40 – 44<br>45 – 49<br>50 – 54<br>55 – 59<br>60 – 64<br>65 – 69<br>70 – 74<br>≥ 75	0<br>2<br>5<br>6<br>8<br>10<br>11<br>12<br>14<br>15	0<br>2<br>4<br>5<br>7<br>8<br>9<br>10<br>11<br>12
	<u>HDL-C (mmol/L)</u><br>> 1.6<br>1.3 – 1.6<br>1.2 – 1.29<br>0.9 – 1.19<br>< 0.9	<u>HDL-C (mmol/L)</u><br>> 1.6<br>1.3 – 1.6<br>1.2 – 1.29<br>0.9 – 1.19<br>< 0.9	 -2<br>-1<br>0<br>1<br>2	
	<u>Total cholesterol (mmol/L)</u>	<u>Total cholesterol (mmol/L)</u>	Male	Female
	< 4.1	< 4.1	0	0

	4.1 - 5.19<br>5.2 - 6.19<br>6.2 - 7.2<br>> 7.2	4.1 - 5.19<br>5.2 - 6.19<br>6.2 - 7.2<br>> 7.2	1<br>2<br>3<br>4	1<br>3<br>4<br>5
	SBP (treated)	SBP (treated)	Male	Female
	< 120 mm Hg<br>120 – 129 mm Hg<br>130 – 139 mm Hg<br>140 – 149 mm Hg<br>150 – 159 mm Hg<br>≥ 160 mm Hg	< 120 mm Hg<br>120 – 129 mm Hg<br>130 – 139 mm Hg<br>140 – 149 mm Hg<br>150 – 159 mm Hg<br>≥ 160 mm Hg	0<br>2<br>3<br>4<br>4<br>5	-1<br>2<br>3<br>5<br>6<br>7
	SBP (untreated)	SBP (untreated)	Male	Female
	< 120 mm Hg<br>120 – 129 mm Hg<br>130 – 139 mm Hg<br>140 – 149 mm Hg<br>150 – 159 mm Hg<br>≥ 160 mm Hg	< 120 mm Hg<br>120 – 129 mm Hg<br>130 – 139 mm Hg<br>140 – 149 mm Hg<br>150 – 159 mm Hg<br>≥ 160 mm Hg	-2<br>0<br>1<br>2<br>2<br>3	-3<br>0<br>1<br>2<br>4<br>5
	Smoking status	Smoking status	Male	Female
	None/former<br>Current	None/former<br>Current	0<br>4	0<br>3
	Diabetes	Diabetes	Male	Female
	No<br>Yes	No<br>Yes (self-reported, linked hospital data, or >6.5% HbA1c)	0<br>3	0<br>4

* Follows UK guidelines which are 140 grams of fish per portion/serving
**Coded in line with Tong et al. (2016)

Abbreviations: Apolipoprotein E (APOE); Body mass index (BMI); Centre for Epidemiologic Studies Depression Scale (CES-D); Coronary heart disease (CHD); Diastolic blood pressure (DBP); Haemoglobin A1C (HbA1c); High-density lipoprotein cholesterol (HDL-C); HInternational Classification of Diseases (ICD); Low-density lipoprotein cholesterol (LDL-C); Systolic blood pressure (SBP); Transient ischaemic attack (TIA); Traumatic brain injury (TBI)

Supplementary Figures

Figure S1. Participant flowchart

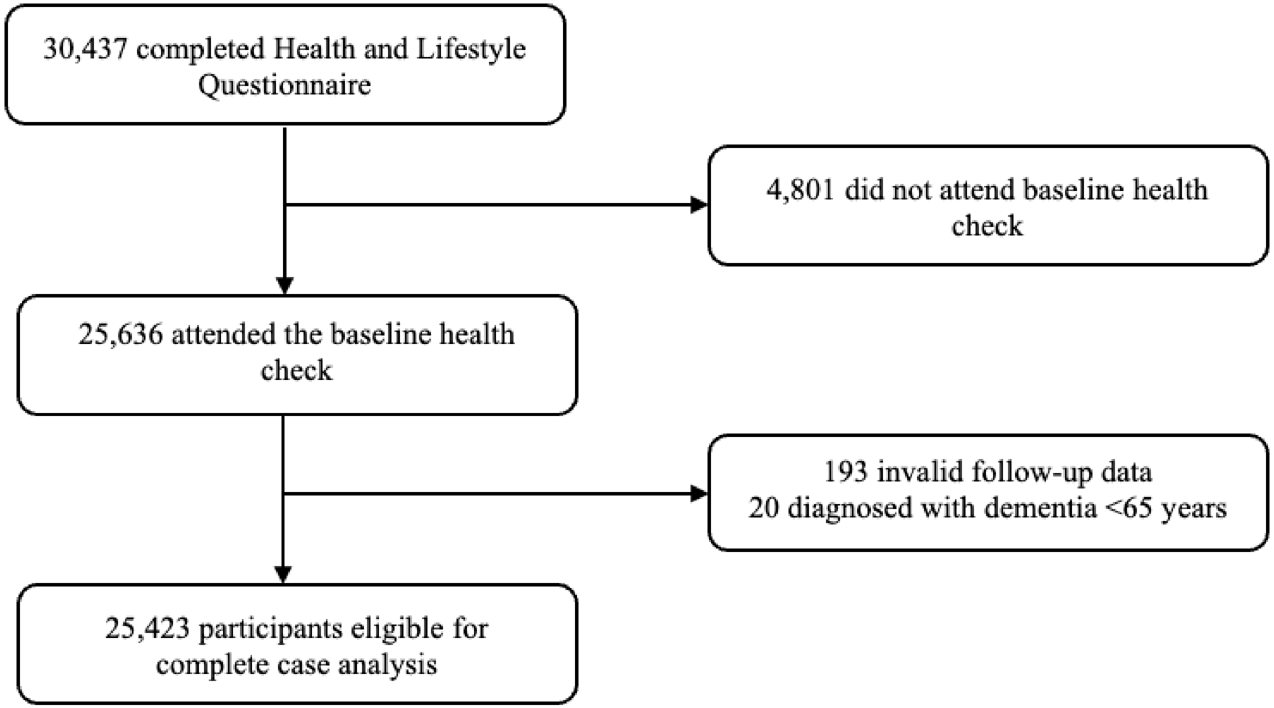

Figures S2: Participant follow-up status over time

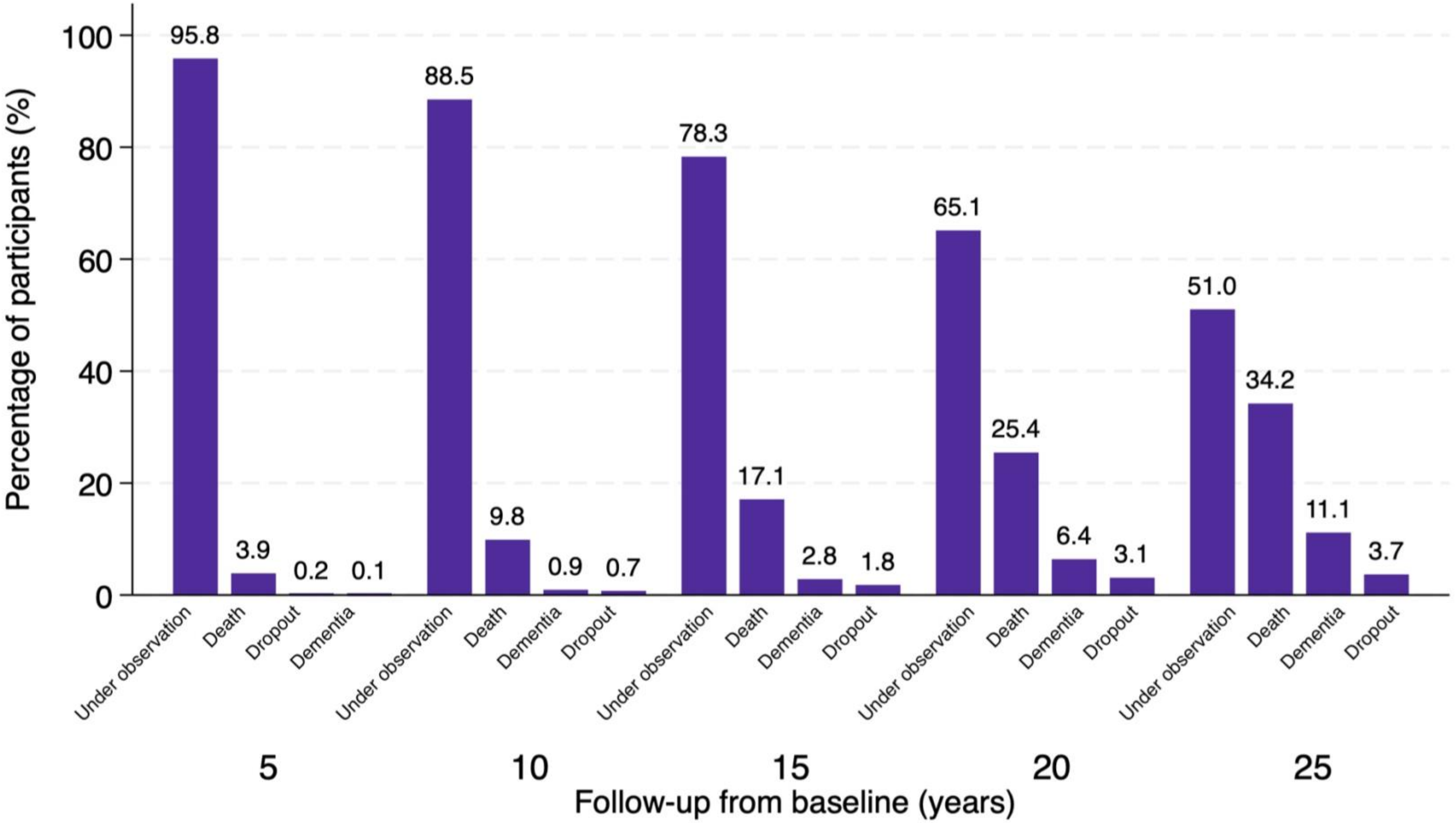

Figures S3: CAIDE calibration plots

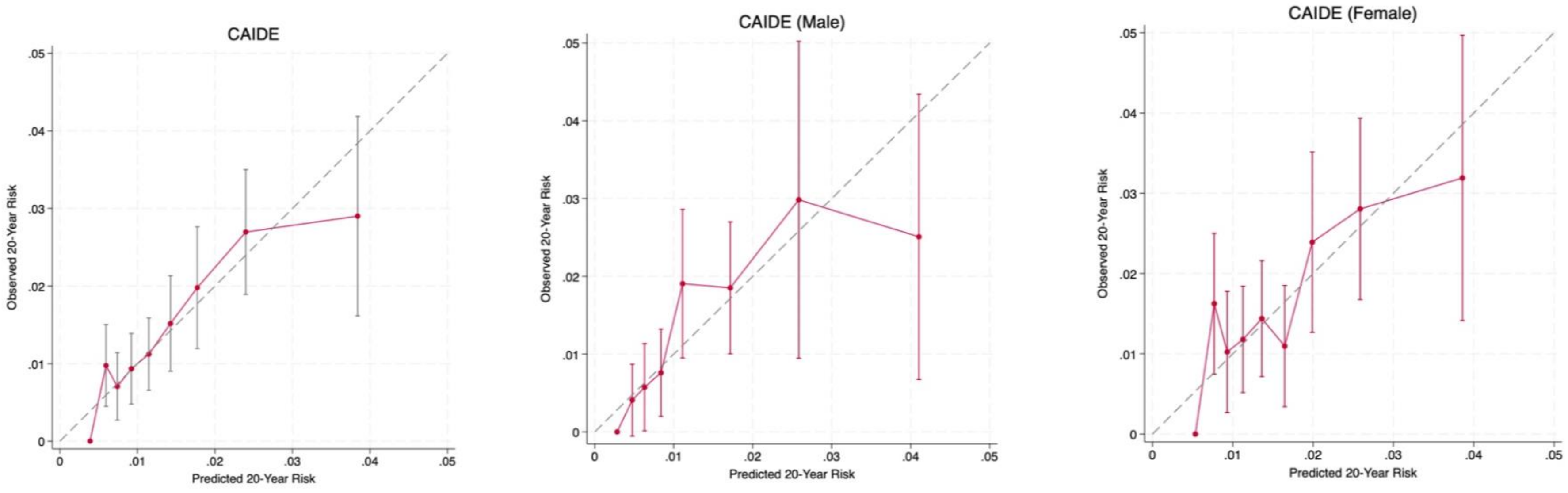

Figure S4: CAIDE-APOE calibration plots

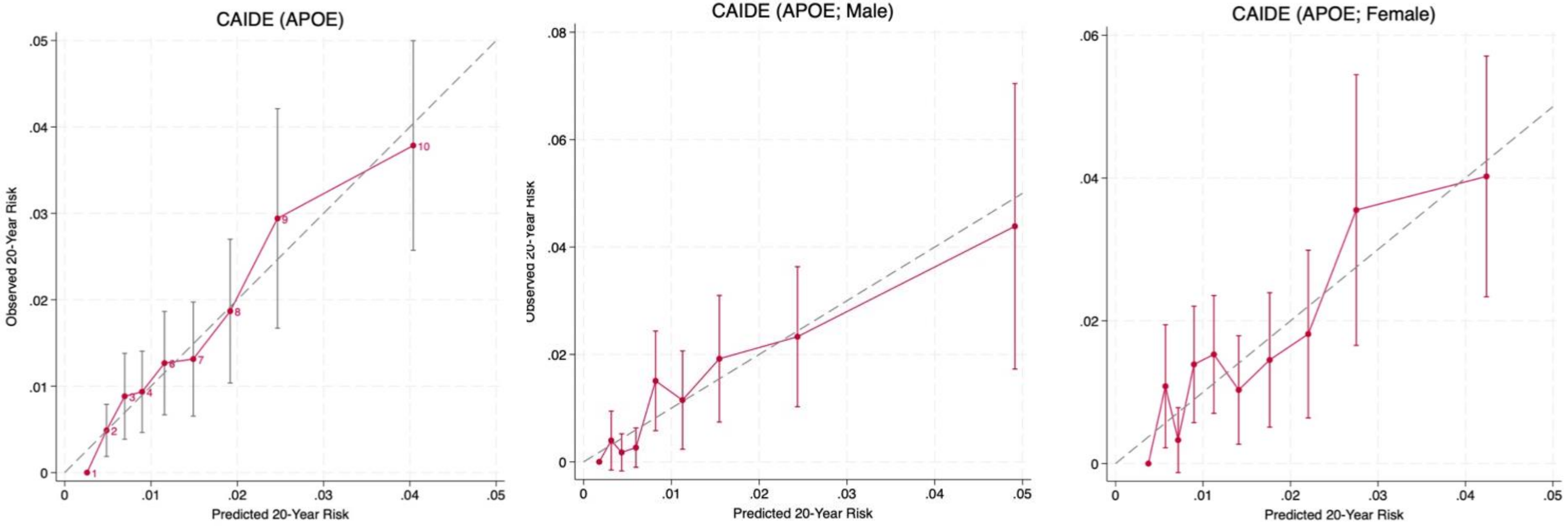

Figure S5. CAIDE (female) recalibration plot

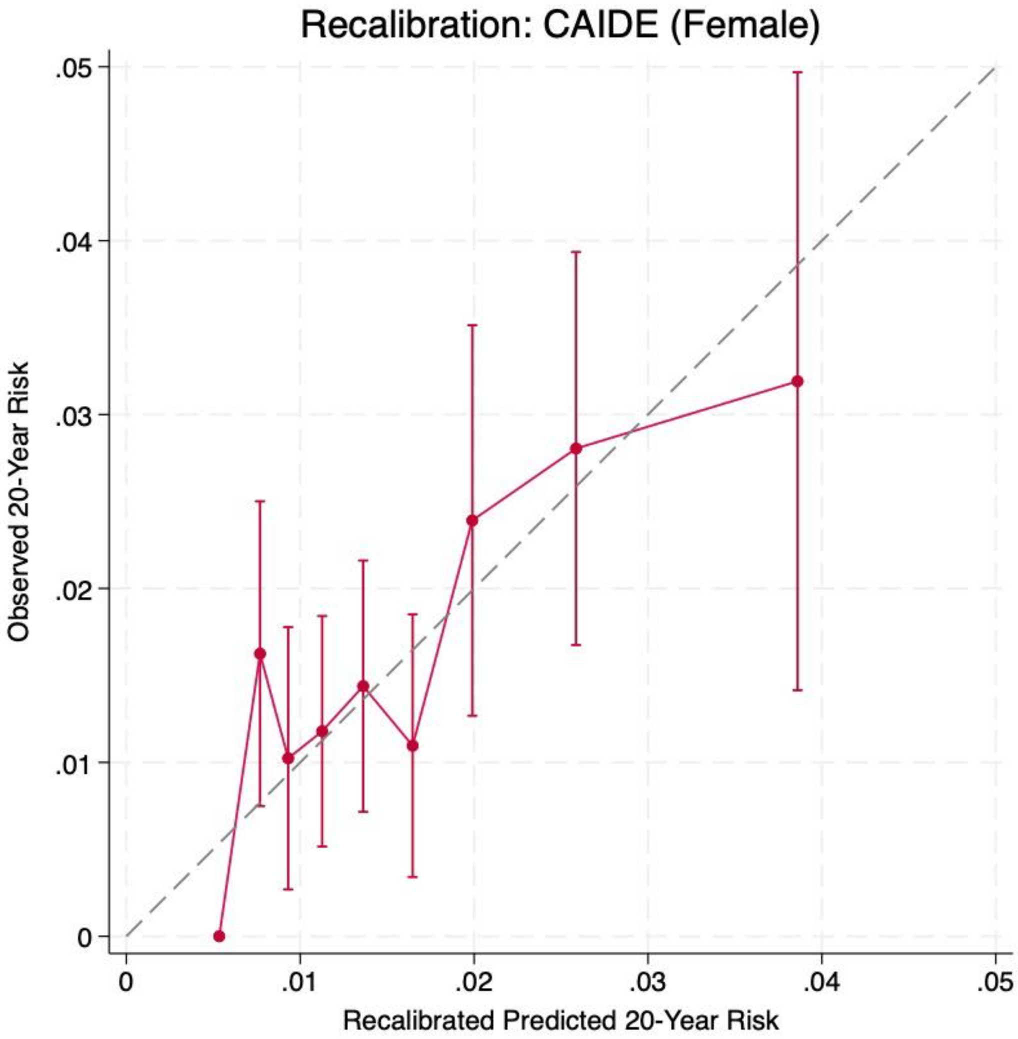

Figure S6: DRS calibration plots

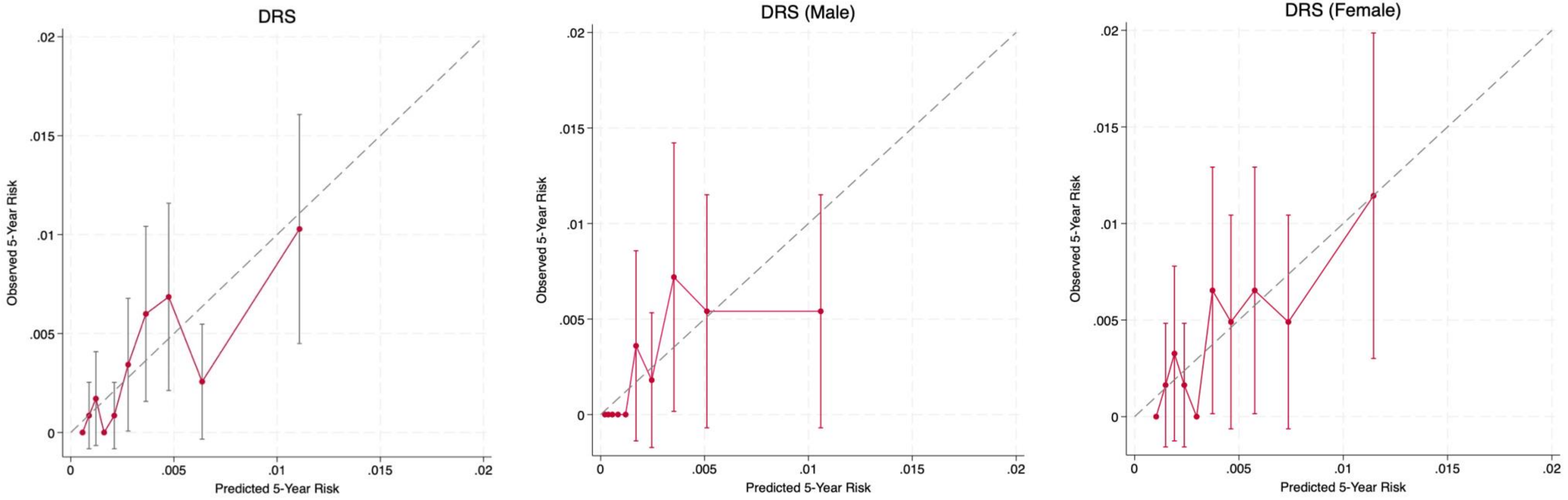

Figure S7: CHA_2DS_2-VASc calibration plots

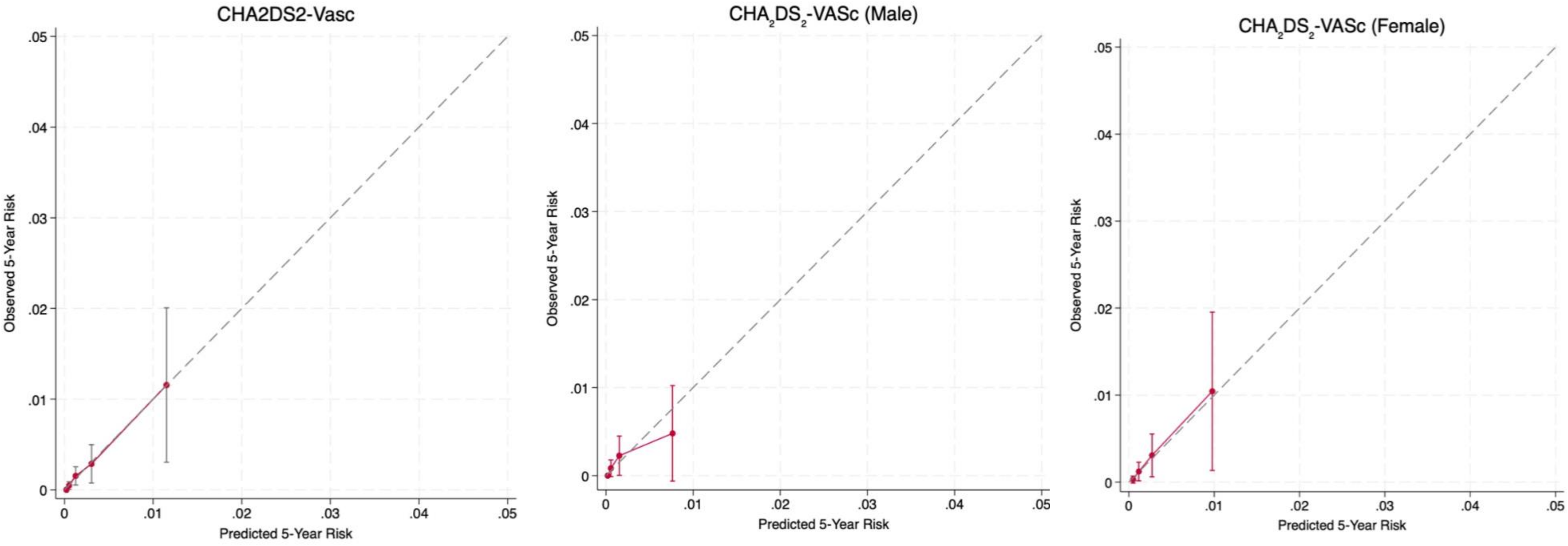

Figure S8: FRS calibration plots

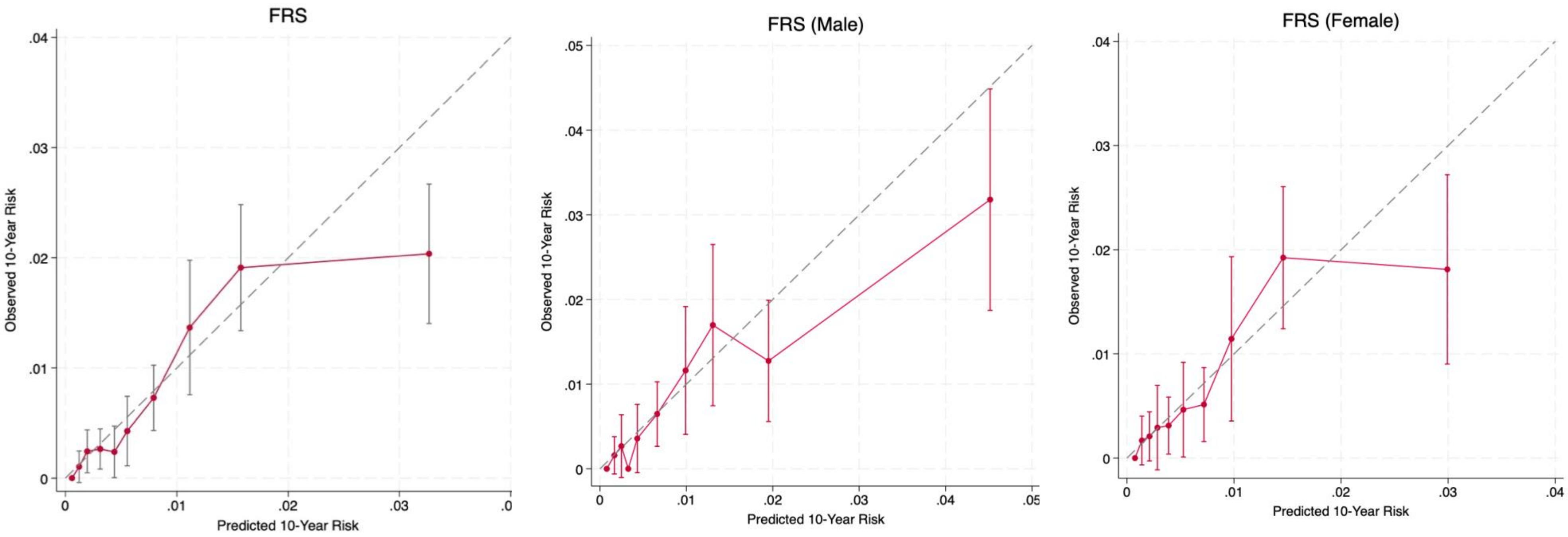

Figure S9: FRS (male) recalibration plot

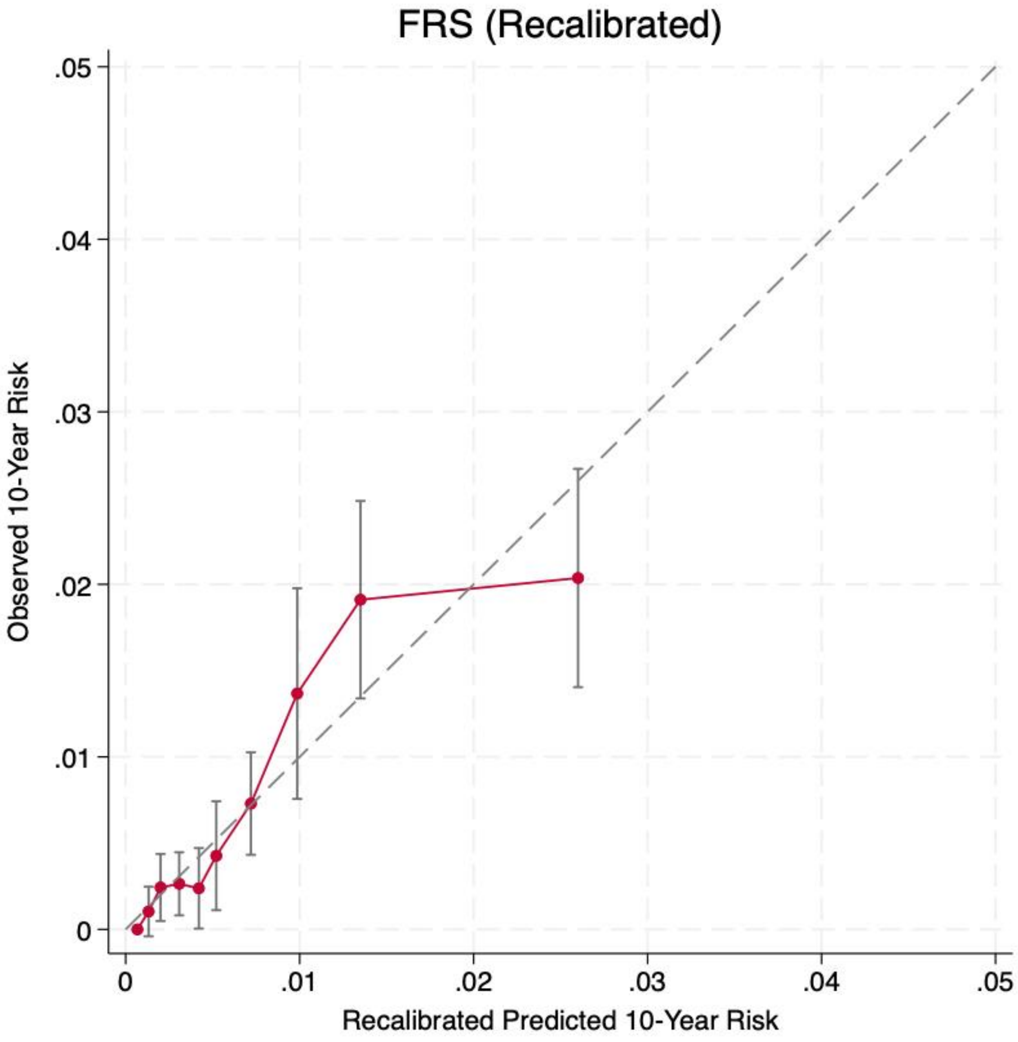

Figure S10: Time-dependent Cox calibration and residual plots

Note: The green solid line represents the ideal calibration line (45-degree reference). The red solid line shows the loess-smoothed observed calibration curve, with the grey shaded area indicating the 95% confidence interval. The yellow dashed line represents the linear calibration fit (calibration slope and intercept). Panels correspond to predictions evaluated at 5, 10, 15, 20, 25, and 30 years after baseline.

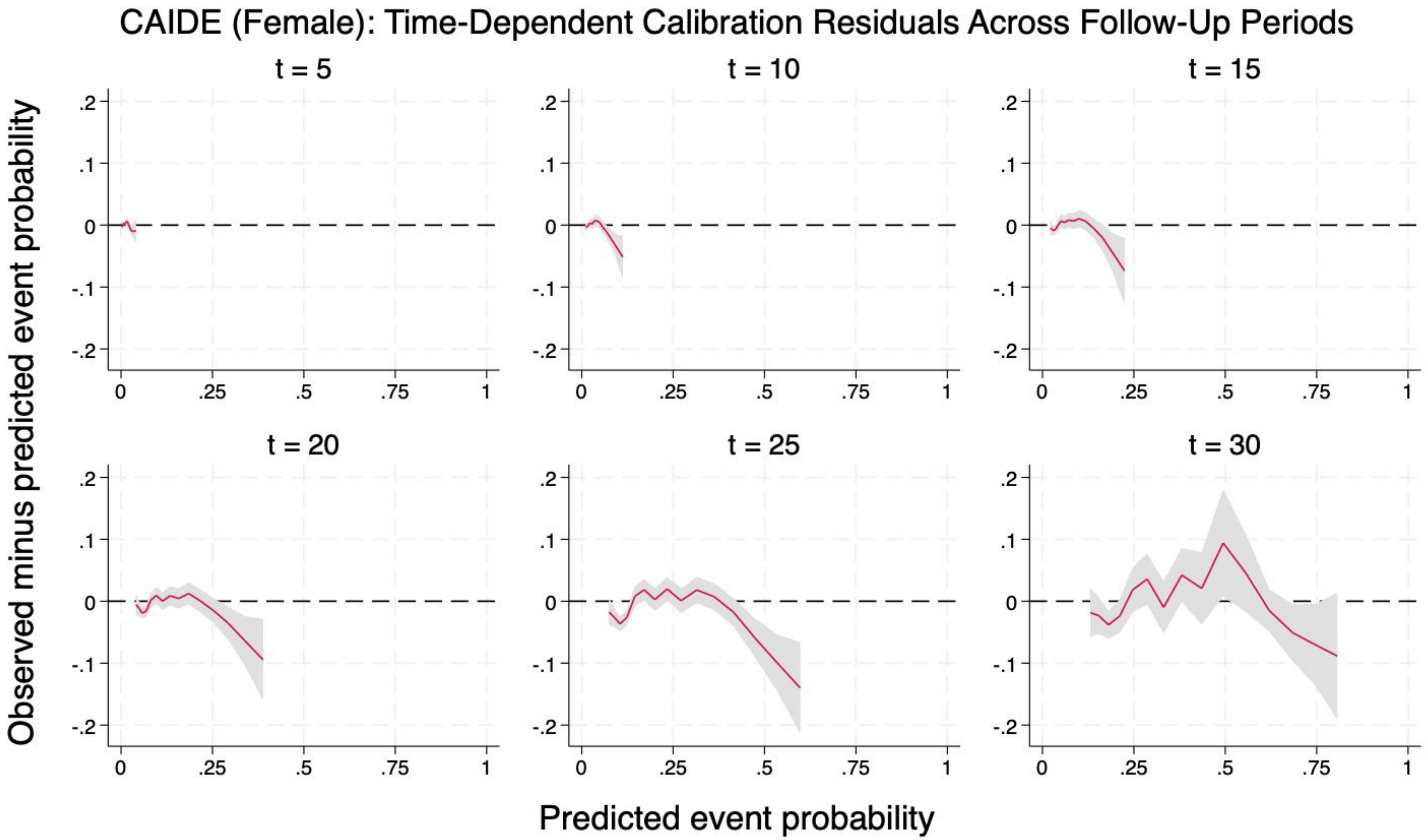
CAIDE (Female): Time-Dependent Calibration Residuals Across Follow-Up Periods
t = 5
t = 10
t = 15
t = 20
t = 25
t = 30
Observed minus predicted event probability
Predicted event probability
.2
.1
0
-.1
-.2
0
.25
.5
.75
1

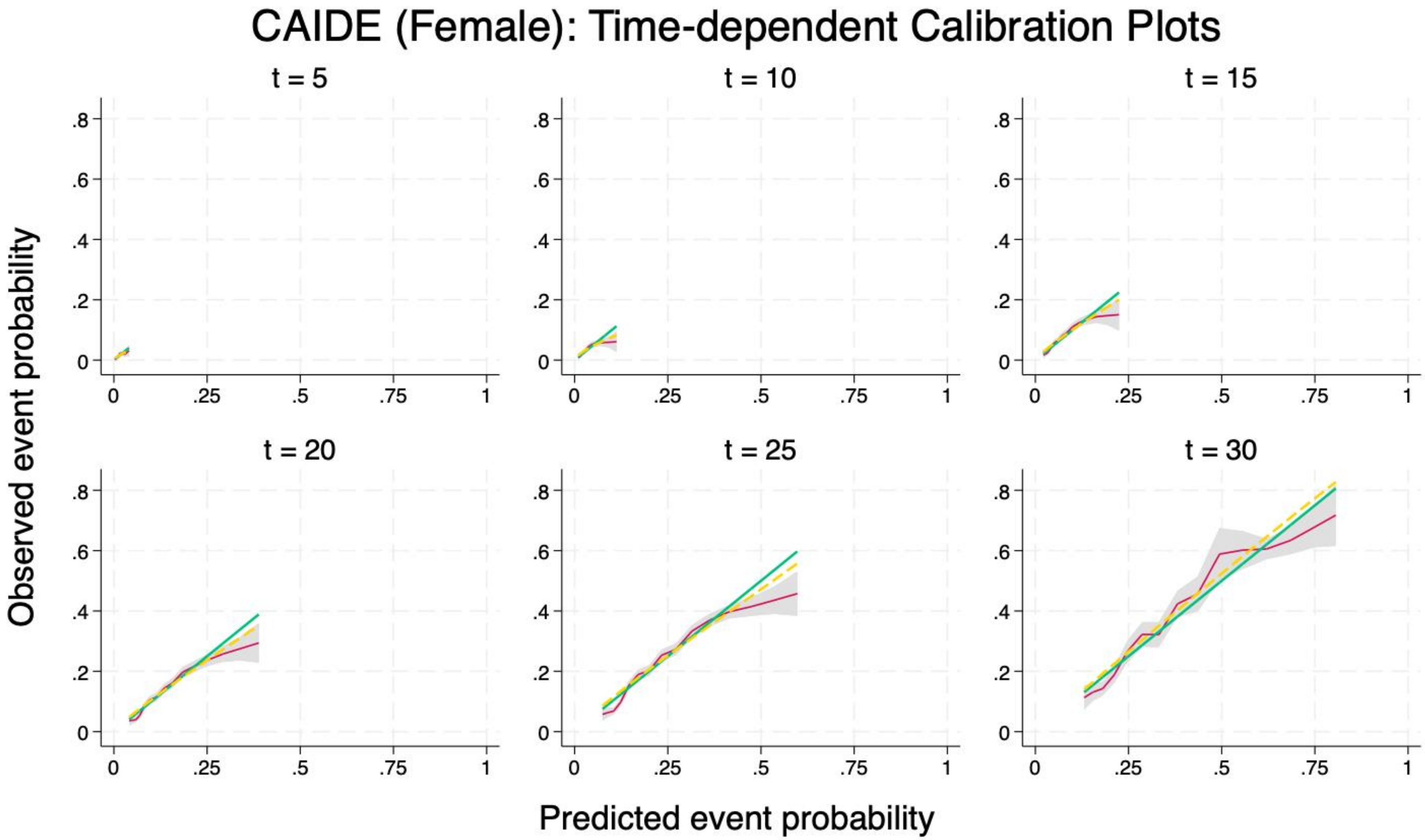

CAIDE (Female): Time-dependent Calibration Plots
t = 5
t = 10
t = 15
t = 20
t = 25
t = 30
Observed event probability
Predicted event probability
0
.2
.4
.6
.8
0
.25
.5
.75
1

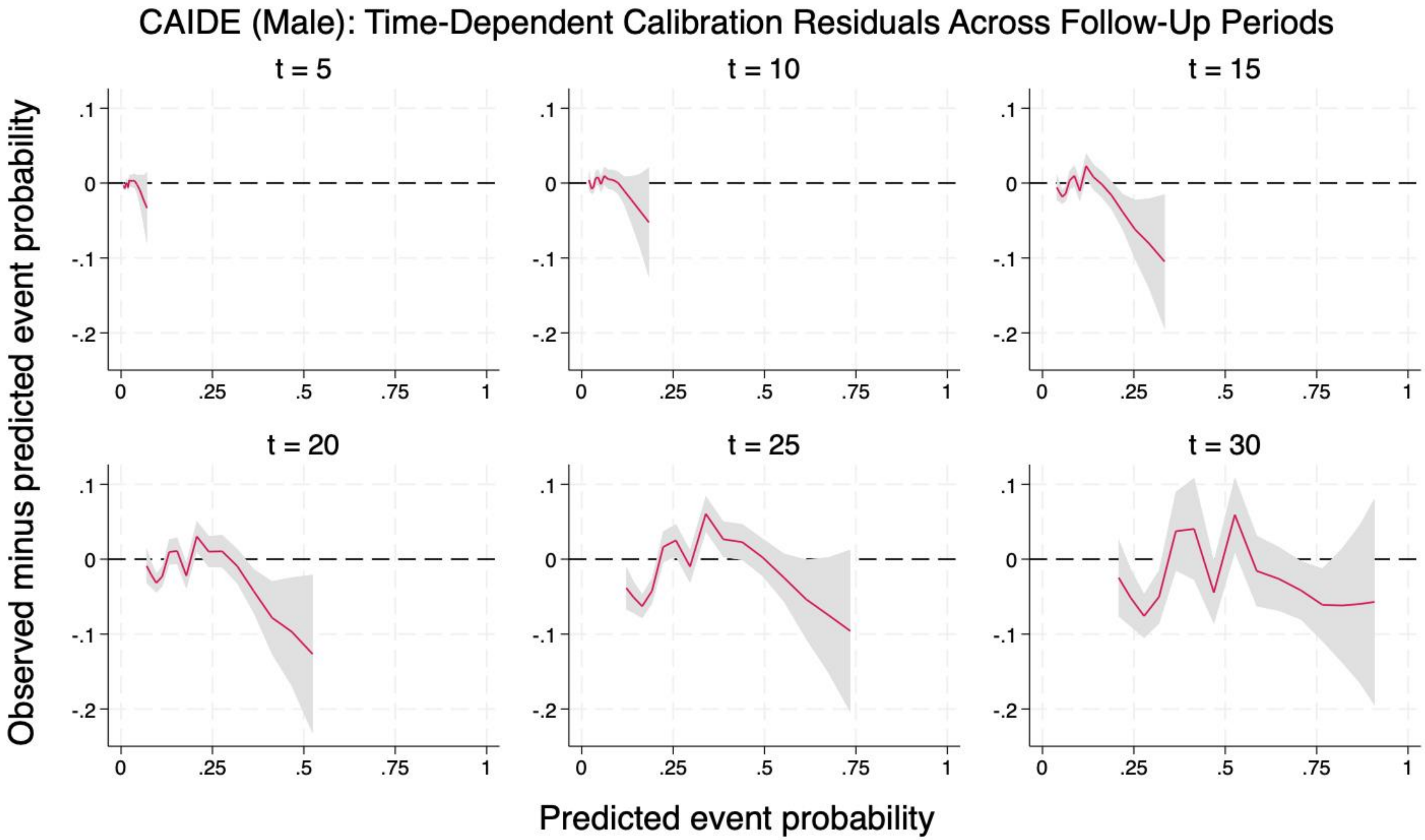

CAIDE (Male): Time-Dependent Calibration Residuals Across Follow-Up Periods
t = 5
t = 10
t = 15
t = 20
t = 25
t = 30
Observed minus predicted event probability
Predicted event probability
.1
0
-.1
-.2
0
.25
.5
.75
1

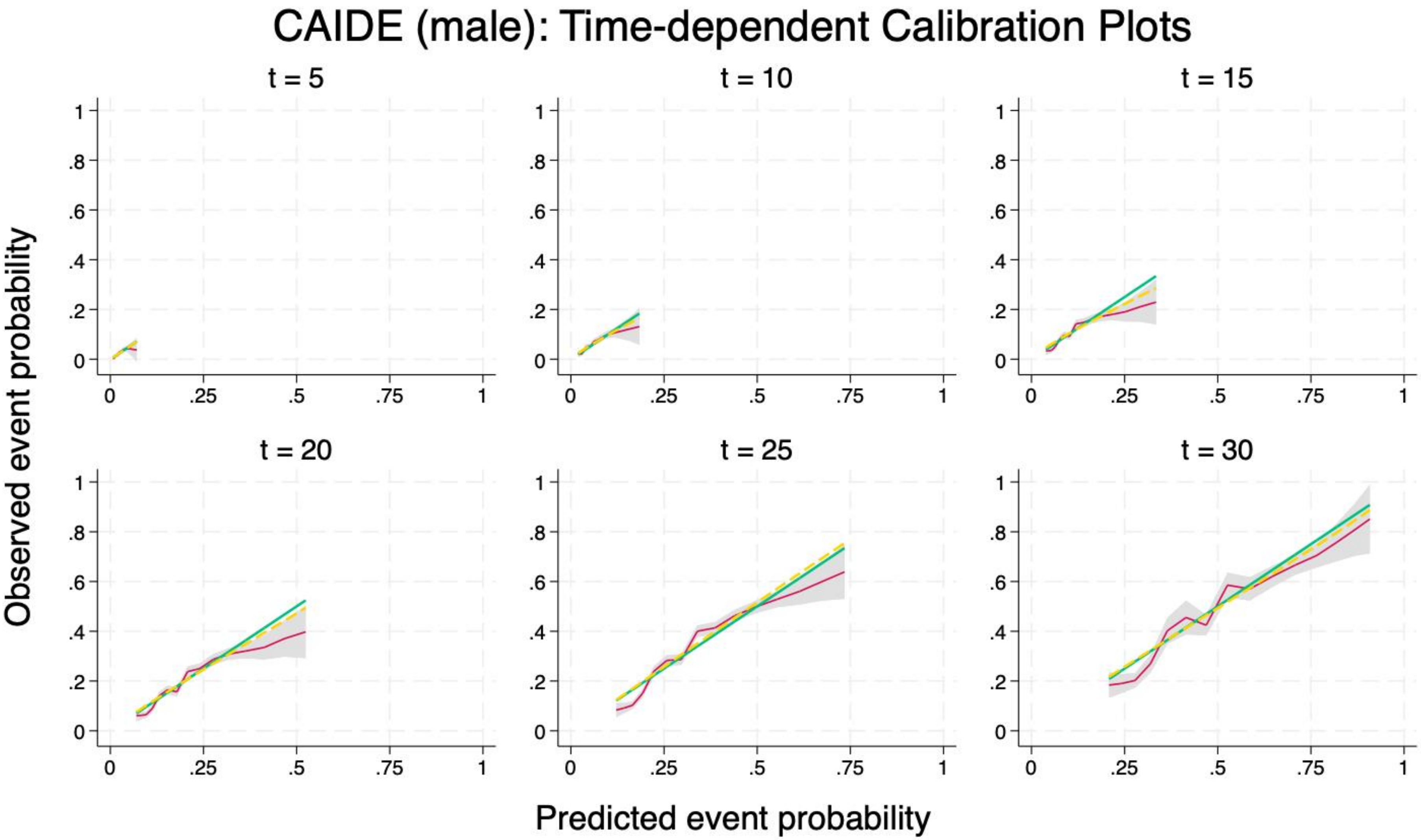
CAIDE (male): Time-dependent Calibration Plots
t = 5
t = 10
t = 15
t = 20
t = 25
t = 30
Observed event probability
Predicted event probability
0
.2
.4
.6
.8
1
.25
.5
.75

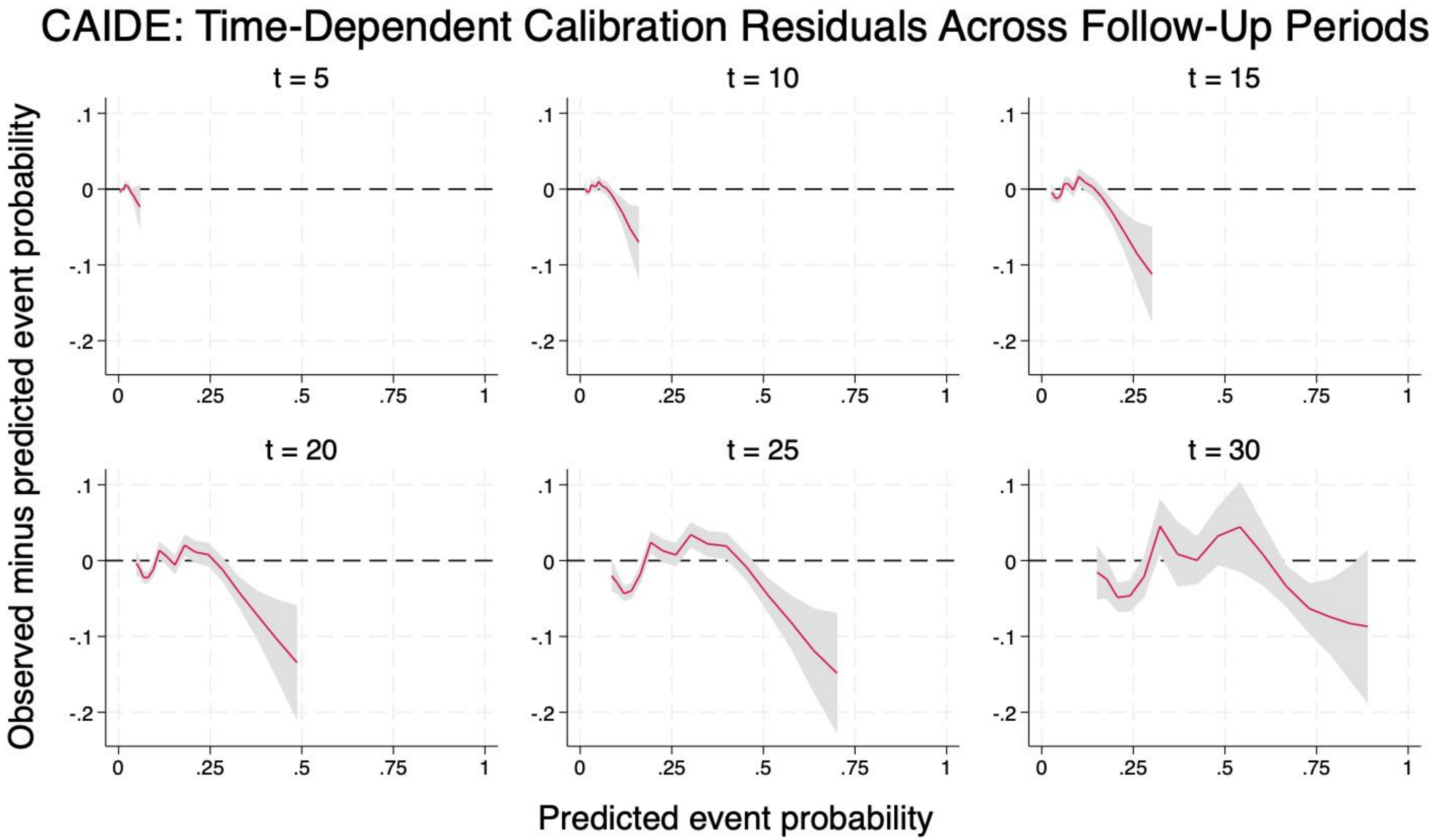

CAIDE: Time-Dependent Calibration Residuals Across Follow-Up Periods
t = 5
t = 10
t = 15
t = 20
t = 25
t = 30
Observed minus predicted event probability
Predicted event probability
.1
0
-.1
-.2
0
.25
.5
.75
1

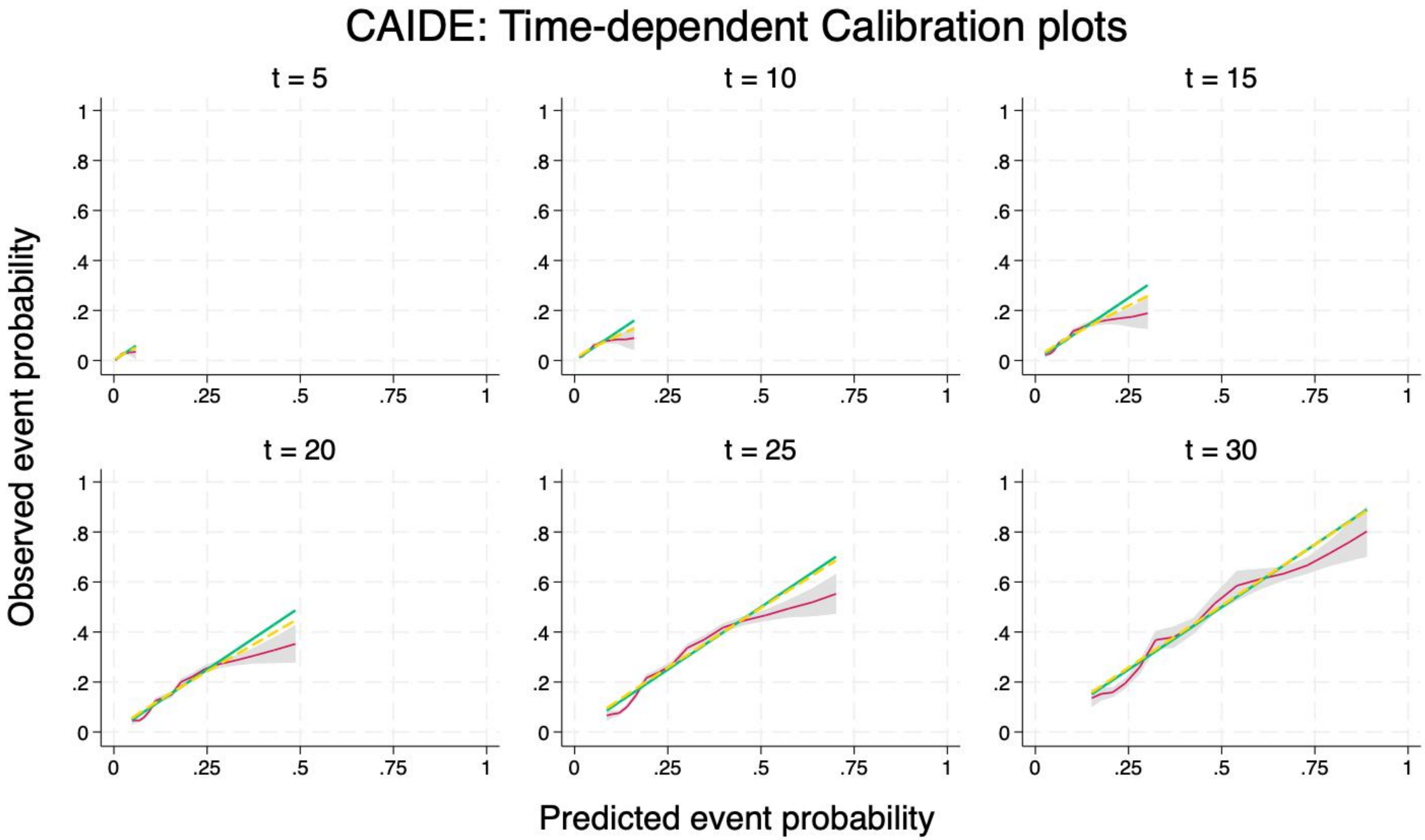
CAIDE: Time-dependent Calibration plots
t = 5
t = 10
t = 15
t = 20
t = 25
t = 30
Observed event probability
Predicted event probability
0
.2
.4
.6
.8
1
0
.25
.5
.75
1

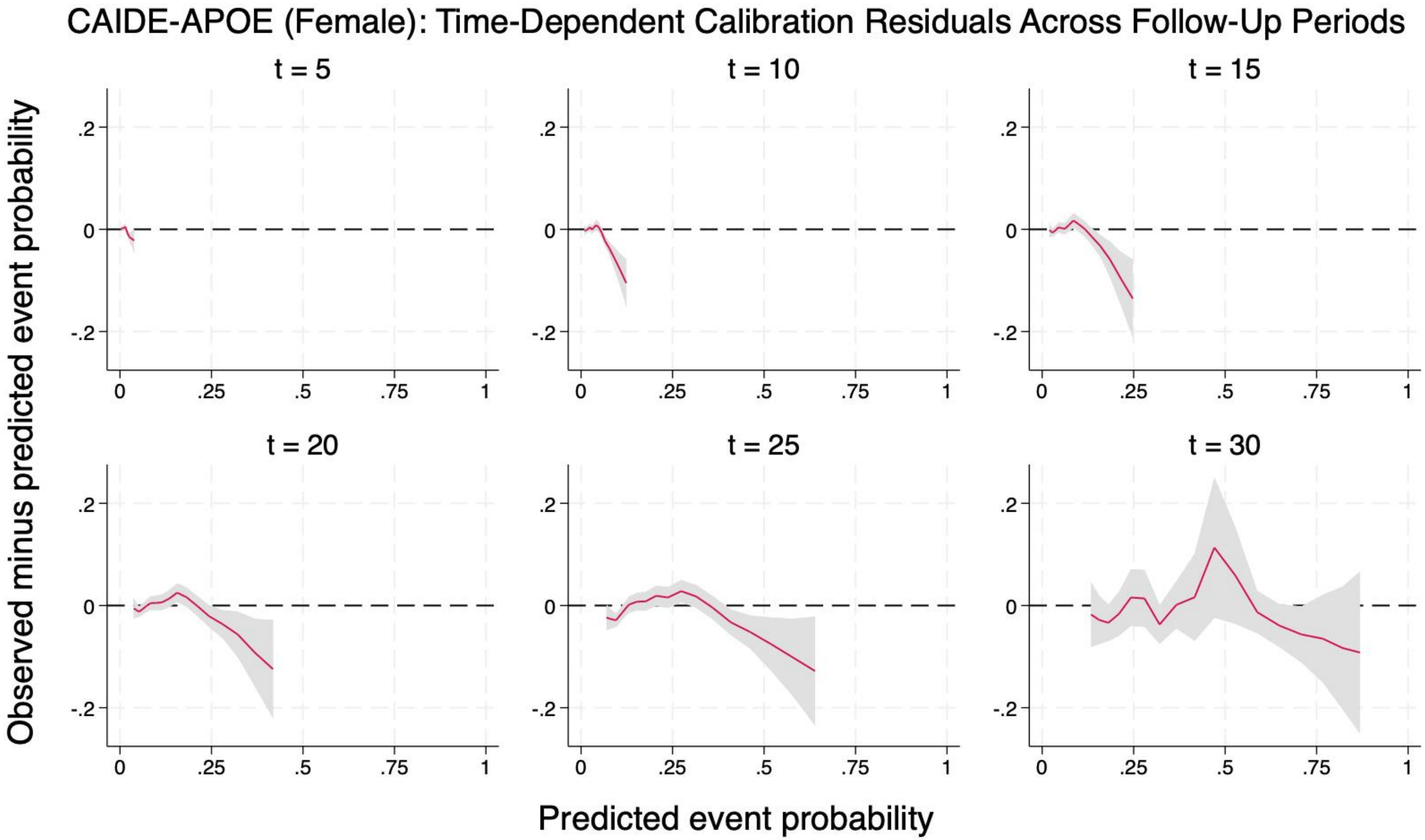
CAIDE-APOE (Female): Time-Dependent Calibration Residuals Across Follow-Up Periods
t = 5
t = 10
t = 15
t = 20
t = 25
t = 30
Observed minus predicted event probability
Predicted event probability
.2
0
-.2
0
.25
.5
.75
1

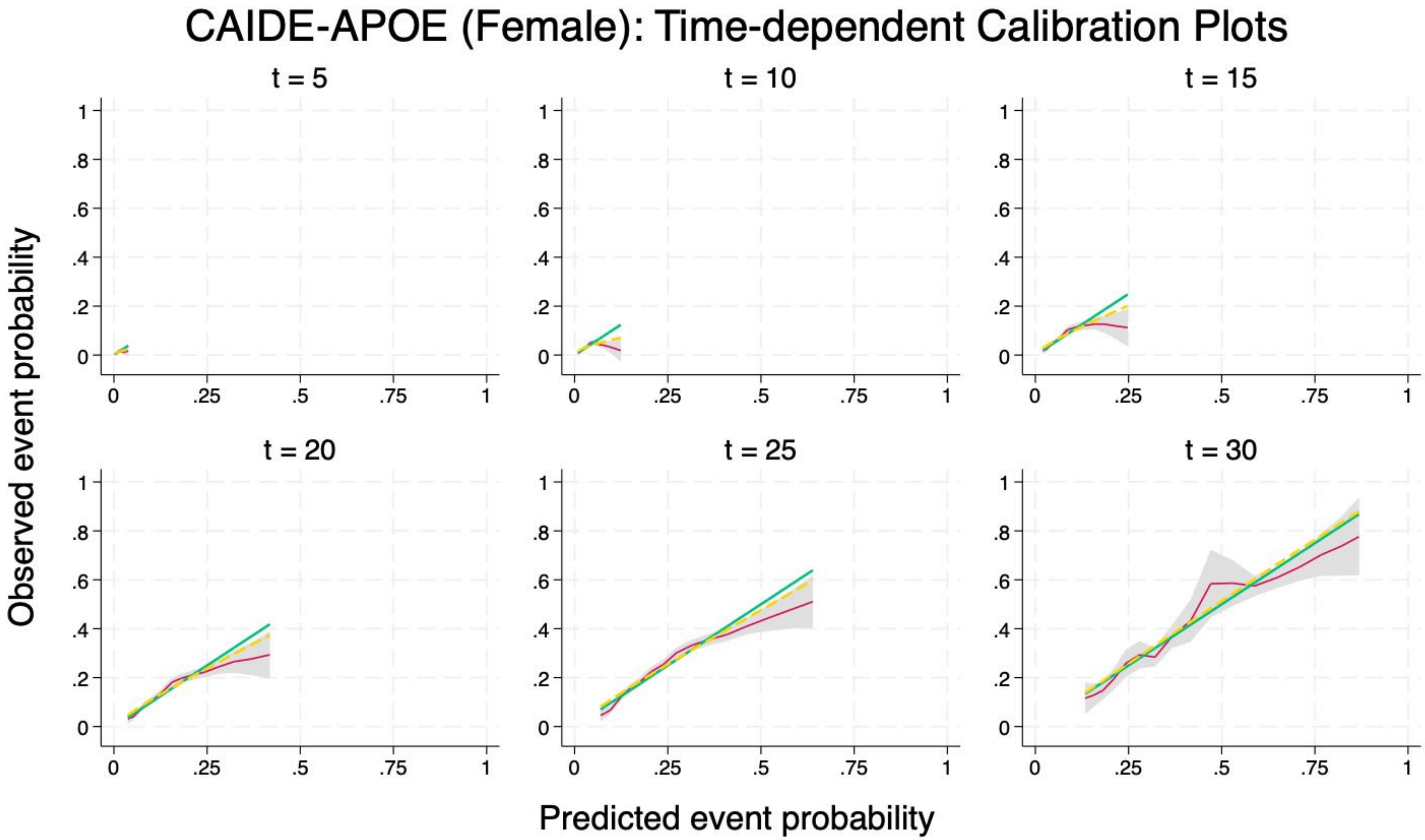
CAIDE-APOE (Female): Time-dependent Calibration Plots
t = 5
t = 10
t = 15
t = 20
t = 25
t = 30
Observed event probability
Predicted event probability
0
.2
.4
.6
.8
1
0
.25
.5
.75
1

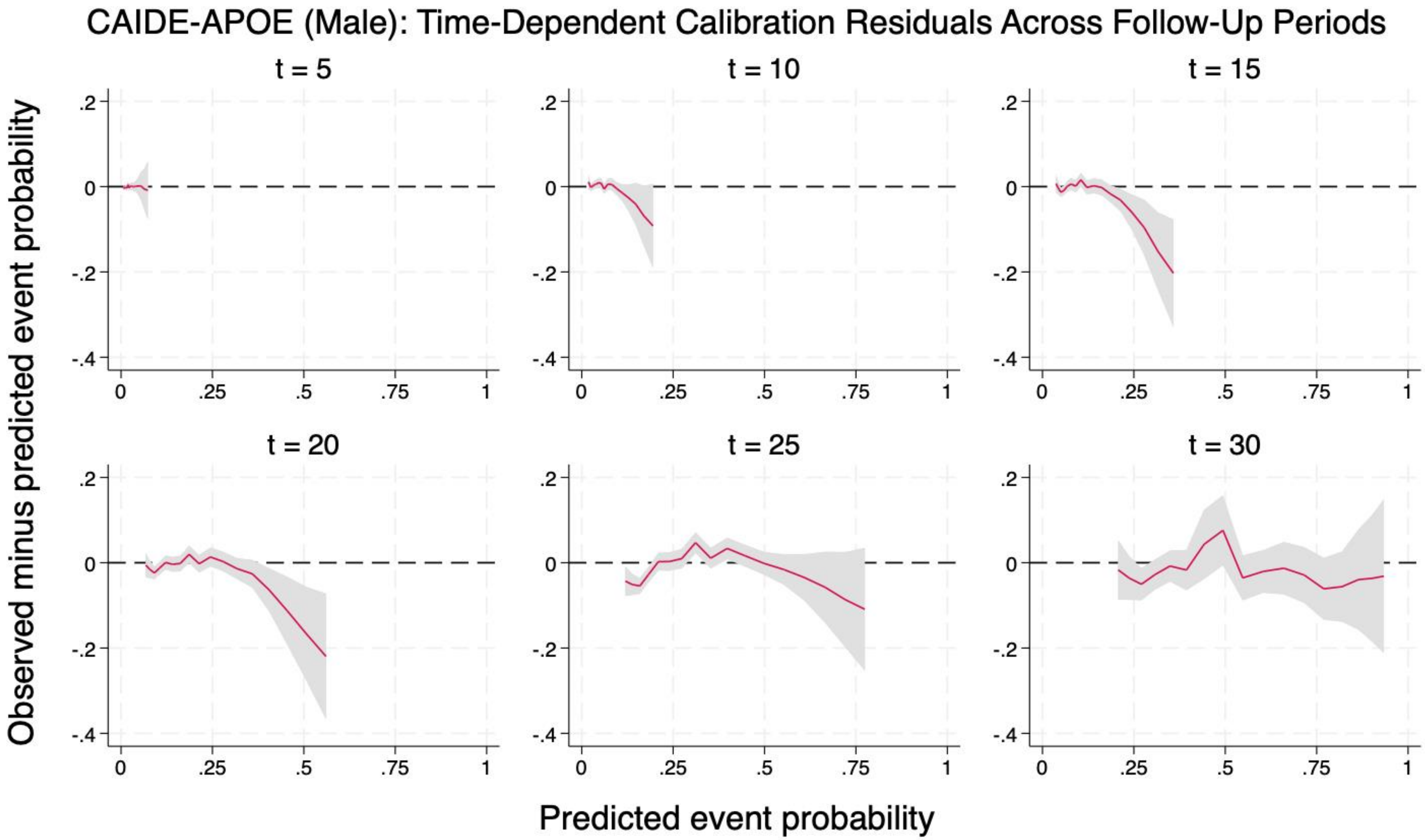

CAIDE-APOE (Male): Time-Dependent Calibration Residuals Across Follow-Up Periods
t = 5
t = 10
t = 15
t = 20
t = 25
t = 30
Observed minus predicted event probability
Predicted event probability
.2
0
-.2
-.4
0
.25
.5
.75
1

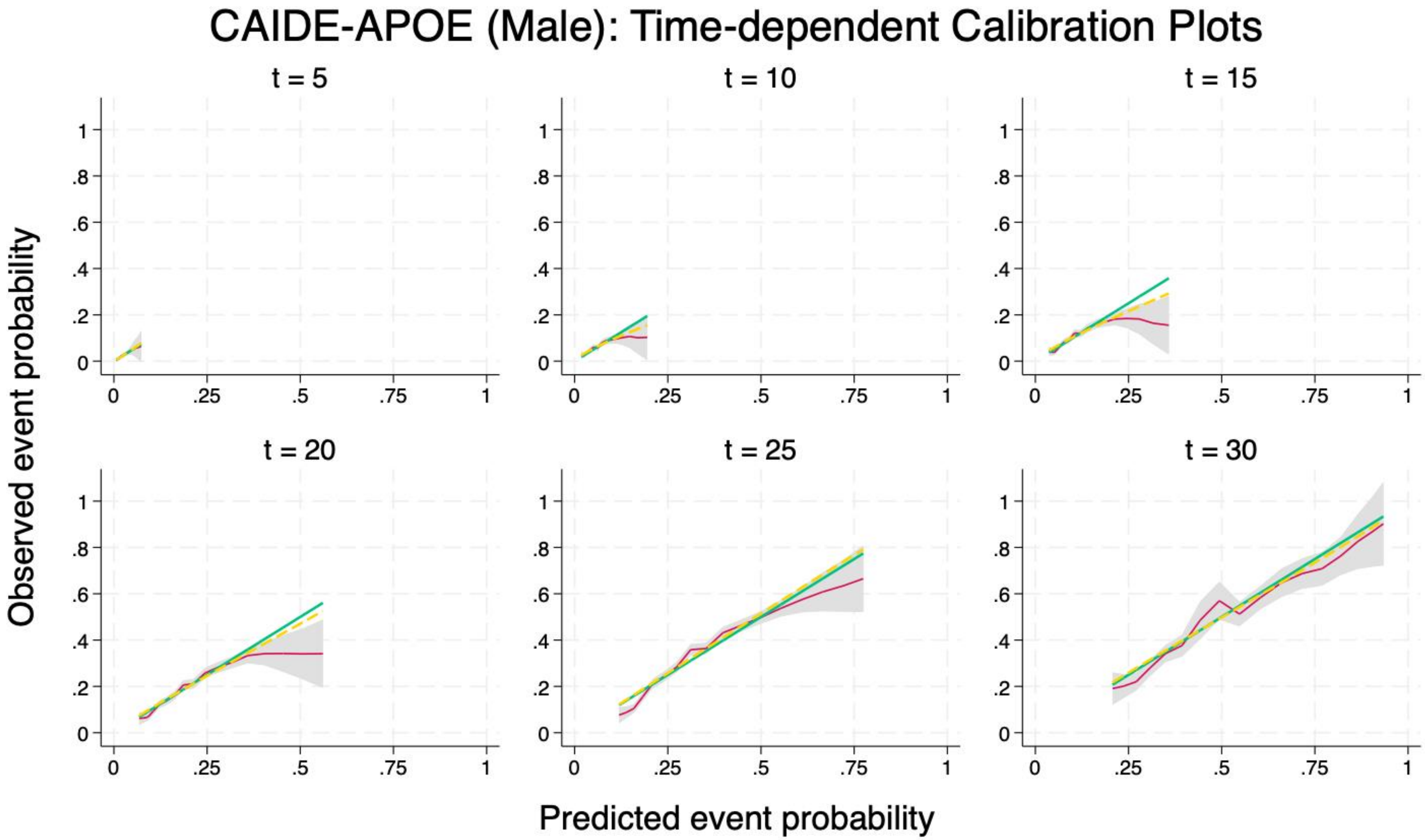

CAIDE-APOE (Male): Time-dependent Calibration Plots
t = 5
t = 10
t = 15
t = 20
t = 25
t = 30
Observed event probability
Predicted event probability
0
.2
.4
.6
.8
1
.25
.5
.75

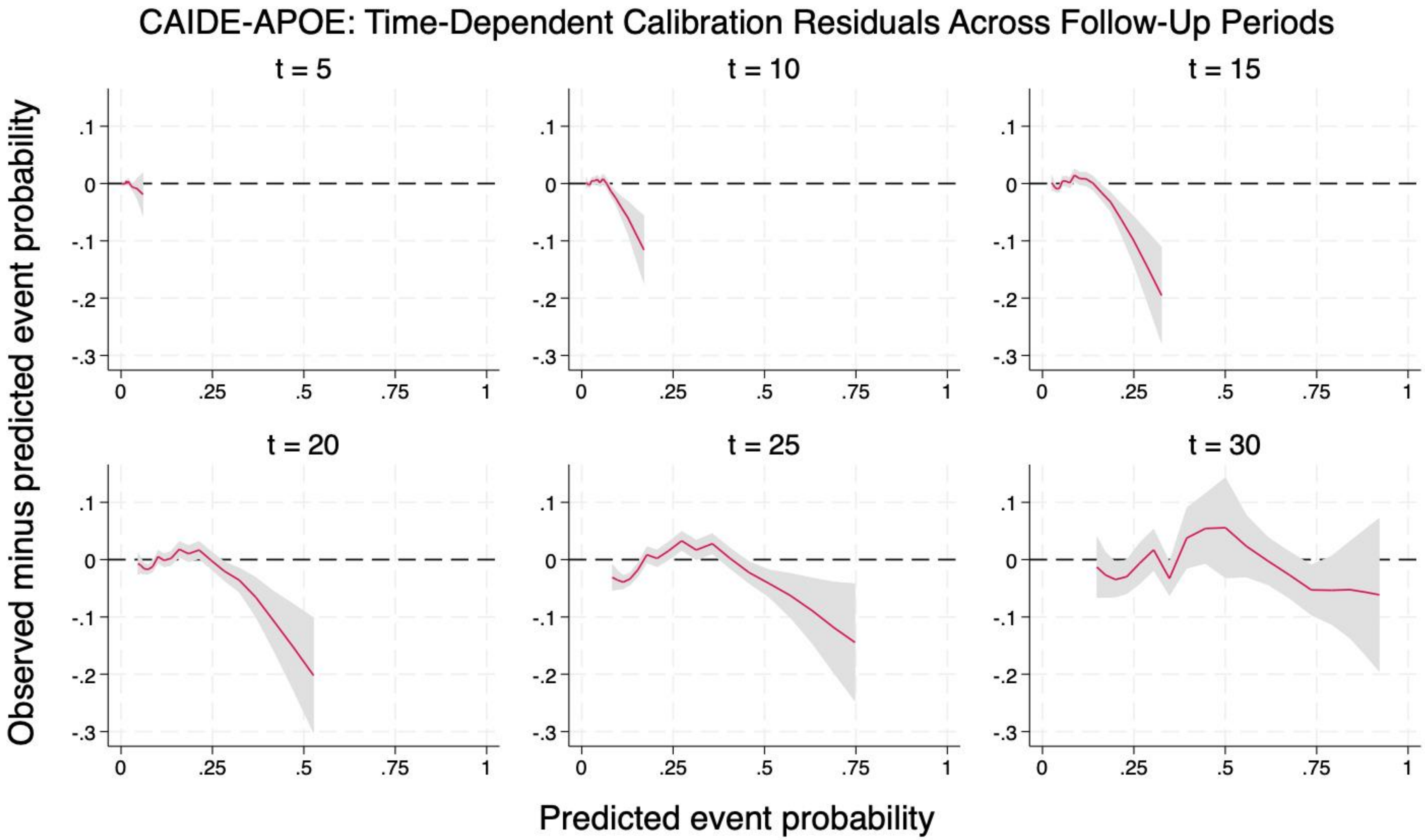
CAIDE-APOE: Time-Dependent Calibration Residuals Across Follow-Up Periods
t = 5
t = 10
t = 15
t = 20
t = 25
t = 30
Observed minus predicted event probability
Predicted event probability
.1
0
-.1
-.2
-.3
0
.25
.5
.75
1

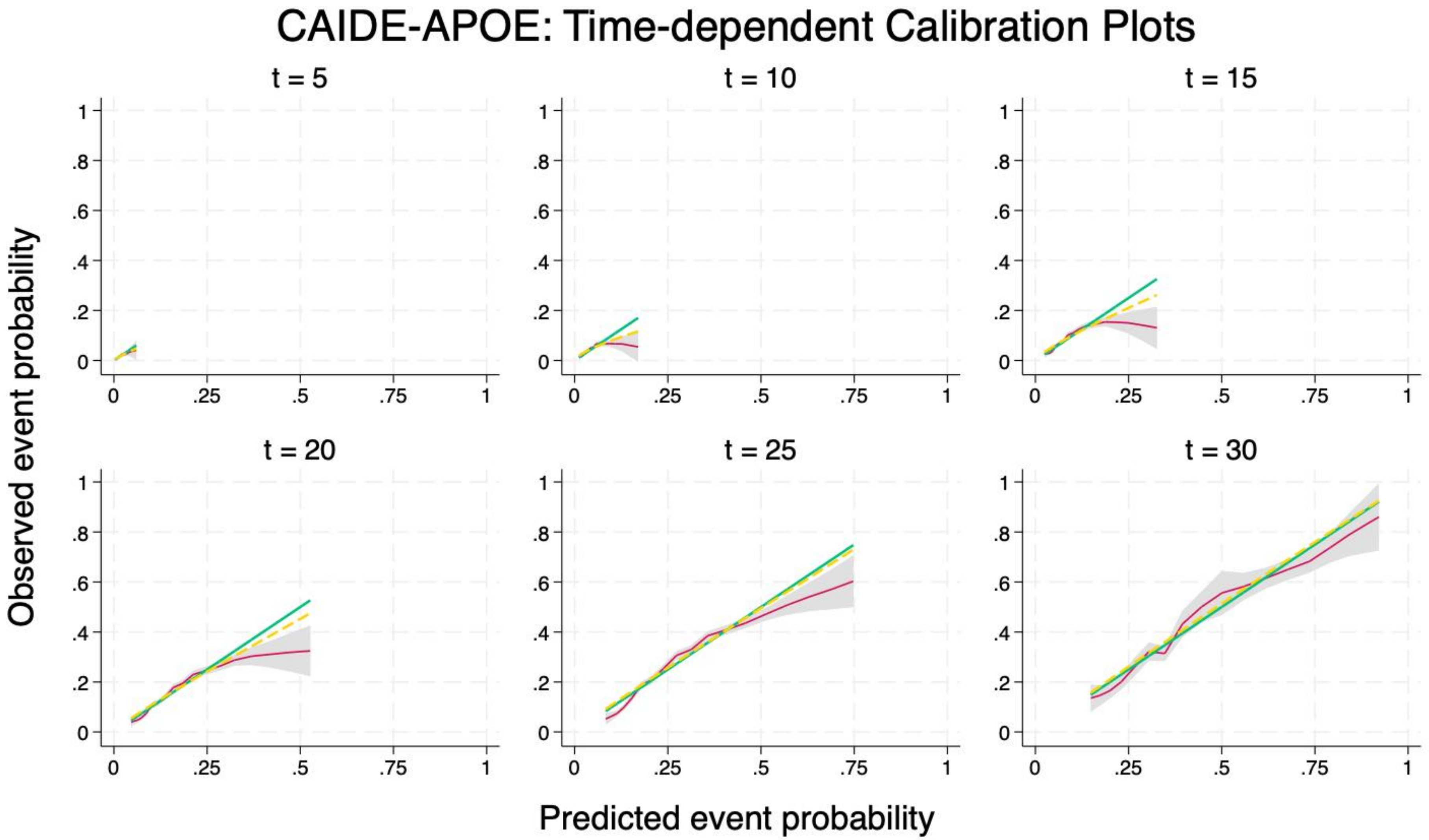
CAIDE-APOE: Time-dependent Calibration Plots
t = 5
t = 10
t = 15
t = 20
t = 25
t = 30
Observed event probability
Predicted event probability
0
.2
.4
.6
.8
1
0
.25
.5
.75
1

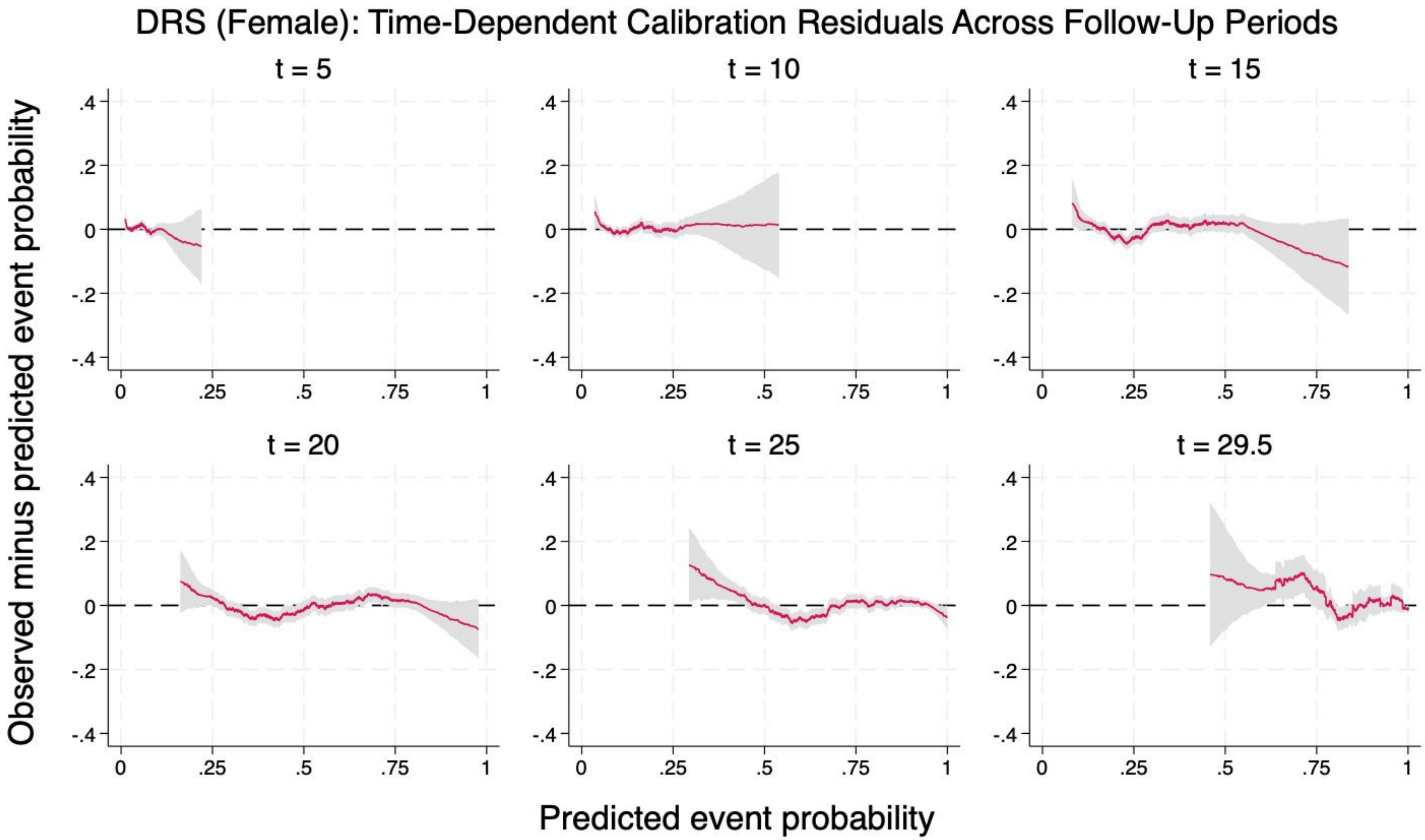

DRS (Female): Time-Dependent Calibration Residuals Across Follow-Up Periods
t = 5
t = 10
t = 15
t = 20
t = 25
t = 29.5
.4
.2
0
-.2
-.4
0
.25
.5
.75
1
Observed minus predicted event probability
Predicted event probability

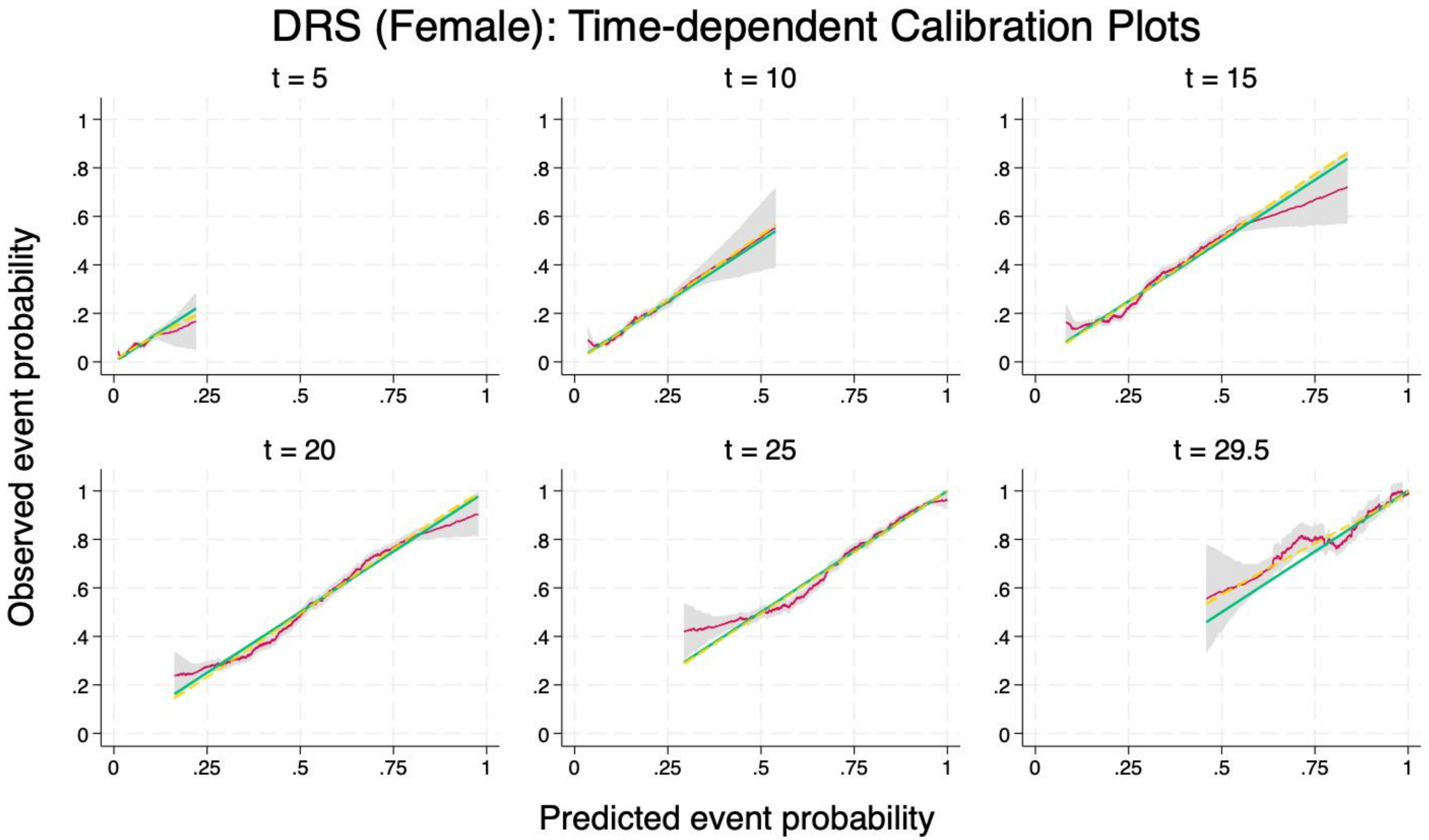
DRS (Female): Time-dependent Calibration Plots
t = 5
t = 10
t = 15
t = 20
t = 25
t = 29.5
Observed event probability
Predicted event probability
0
.2
.4
.6
.8
1
0
.25
.5
.75
1

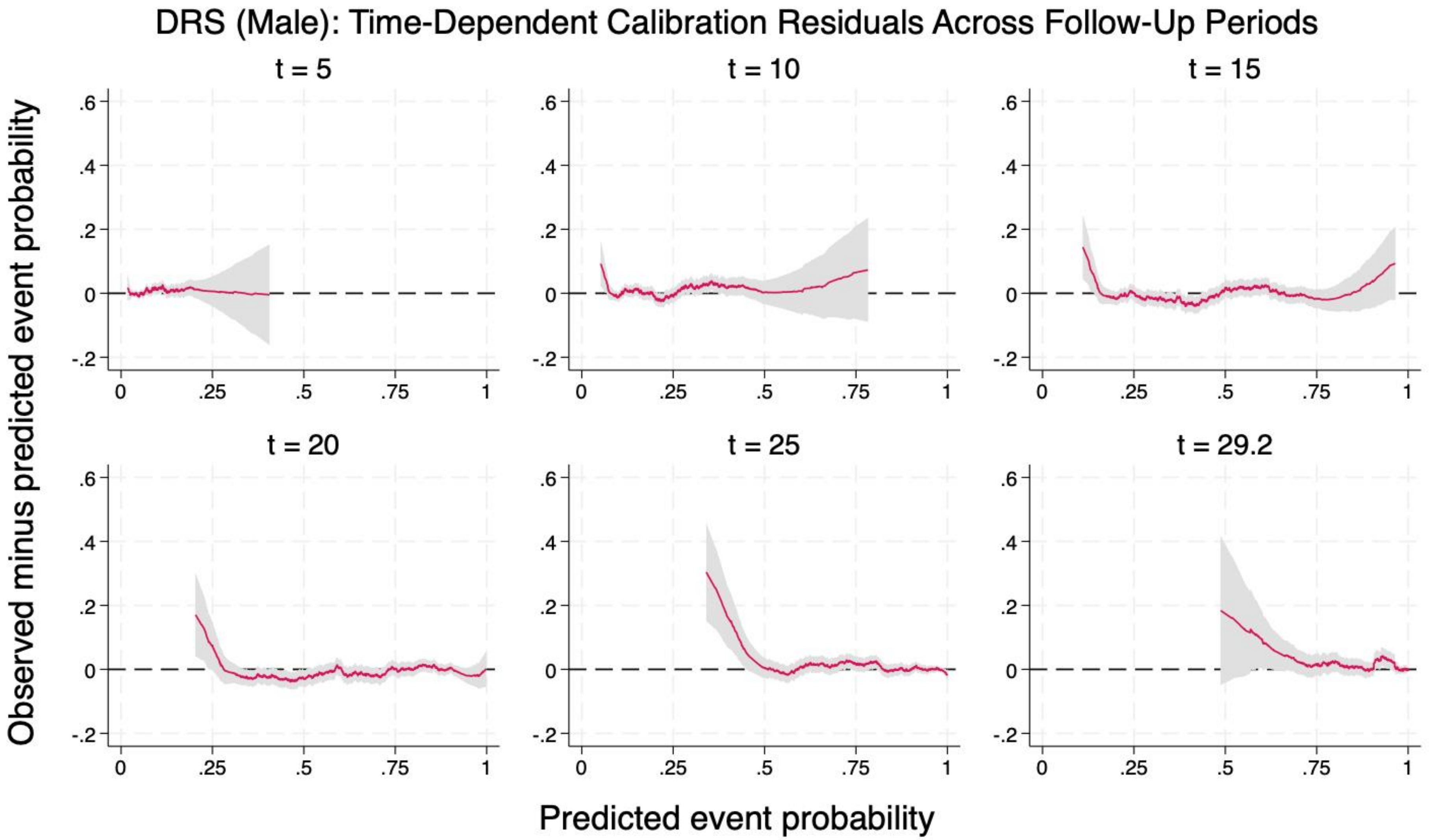
DRS (Male): Time-Dependent Calibration Residuals Across Follow-Up Periods
t = 5
t = 10
t = 15
t = 20
t = 25
t = 29.2
.6
.4
.2
0
-.2
0
.25
.5
.75
1
Observed minus predicted event probability
Predicted event probability

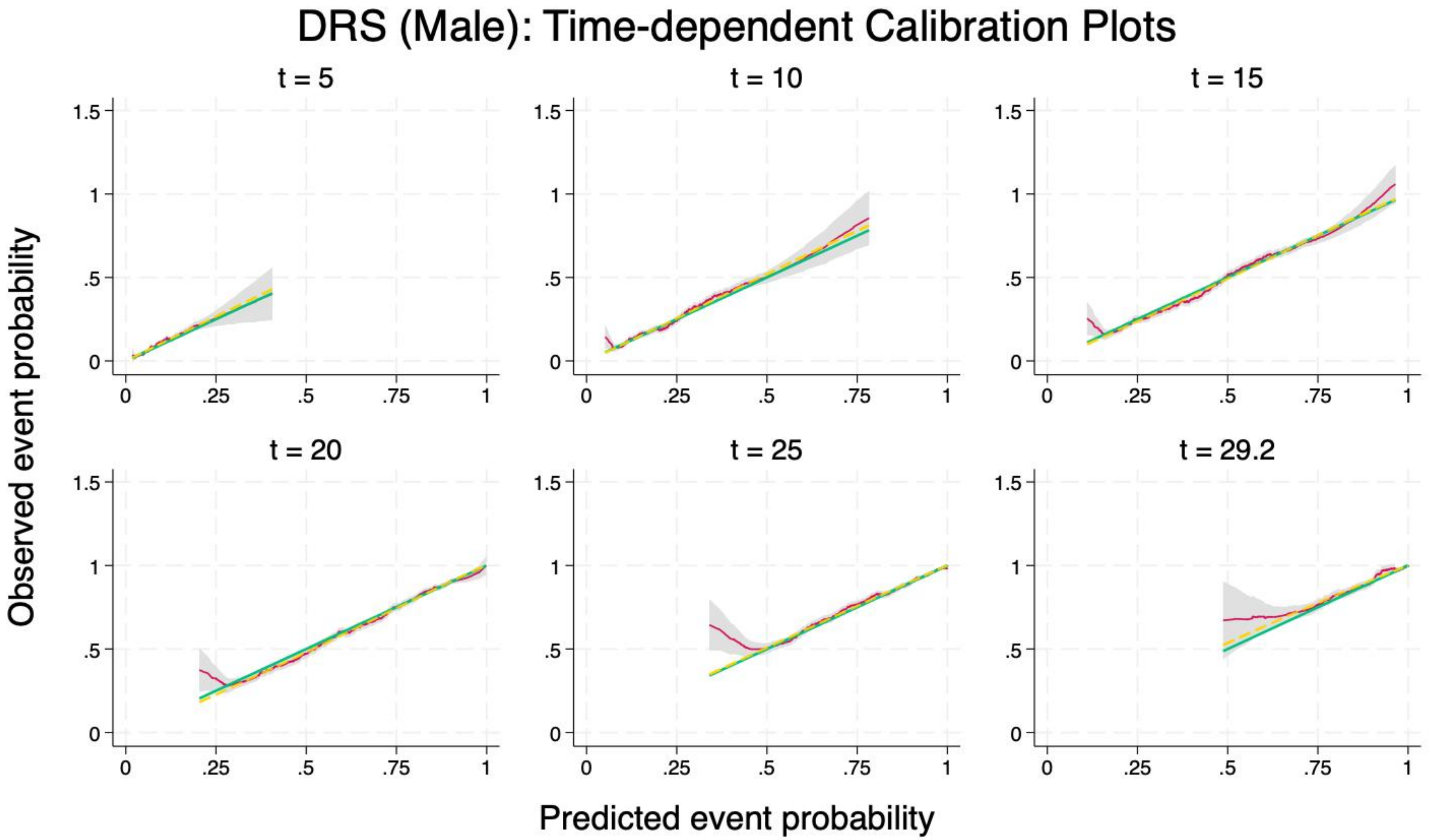
DRS (Male): Time-dependent Calibration Plots
t = 5
t = 10
t = 15
t = 20
t = 25
t = 29.2
Observed event probability
Predicted event probability
0
.25
.5
.75
1
1.5

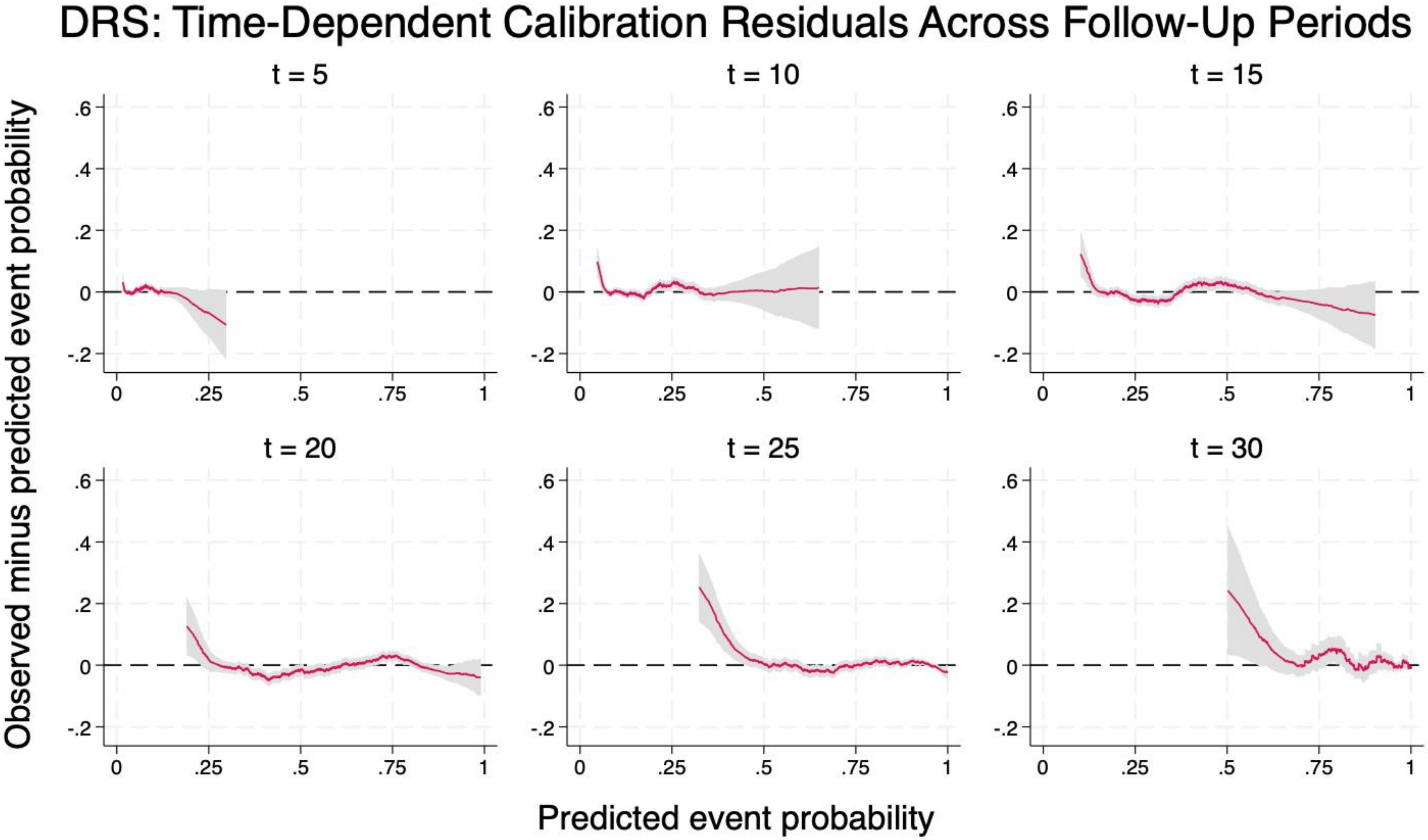
DRS: Time-Dependent Calibration Residuals Across Follow-Up Periods
t = 5
t = 10
t = 15
t = 20
t = 25
t = 30
Observed minus predicted event probability
Predicted event probability
.6
.4
.2
0
-.2
0
.25
.5
.75
1

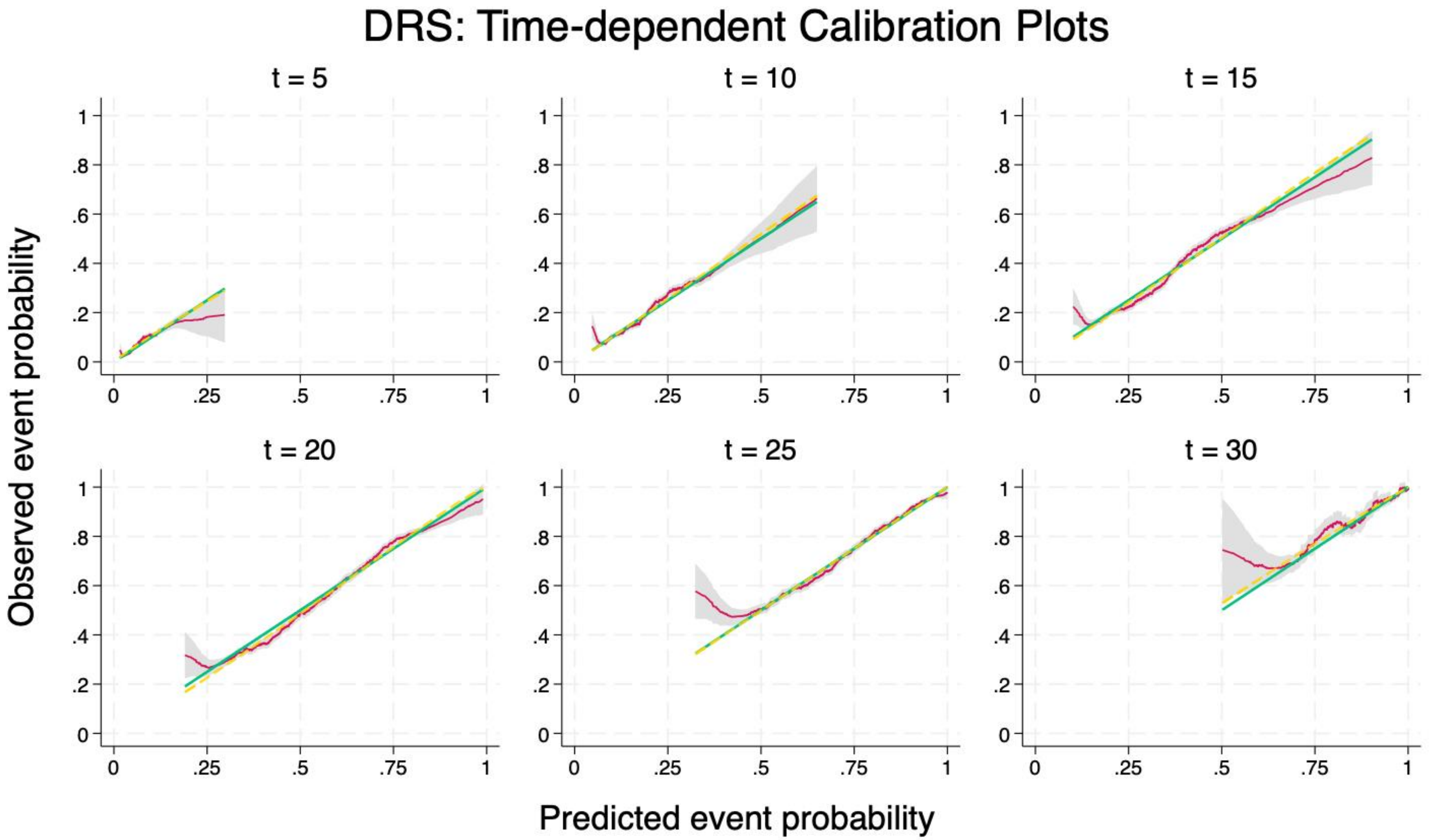
DRS: Time-dependent Calibration Plots
t = 5
t = 10
t = 15
t = 20
t = 25
t = 30
Observed event probability
Predicted event probability
0
.2
.4
.6
.8
1
0
.25
.5
.75
1

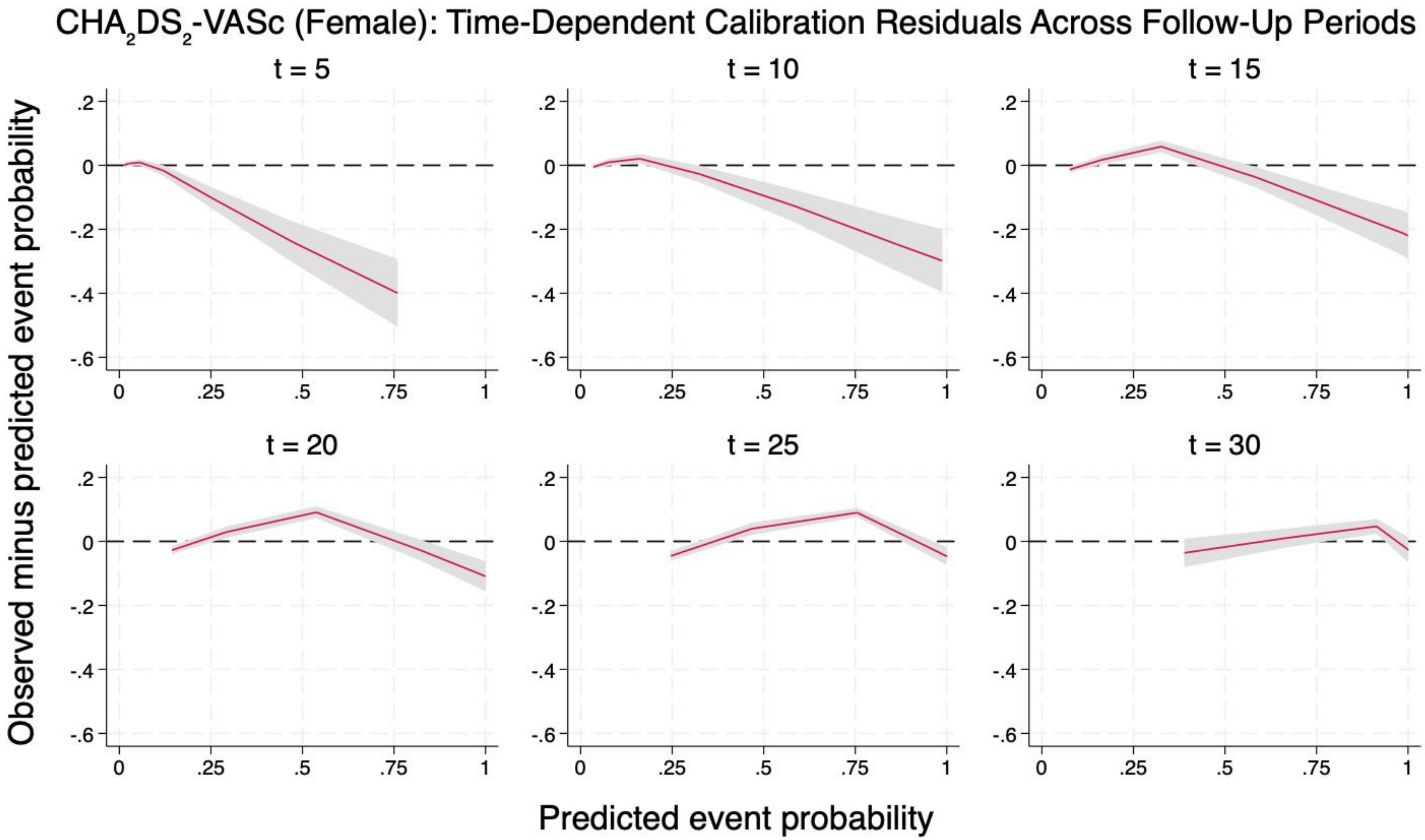
CHA2DS2-VASc (Female): Time-Dependent Calibration Residuals Across Follow-Up Periods
t = 5
t = 10
t = 15
t = 20
t = 25
t = 30
Observed minus predicted event probability
Predicted event probability
.2
0
-.2
-.4
-.6
0
.25
.5
.75
1

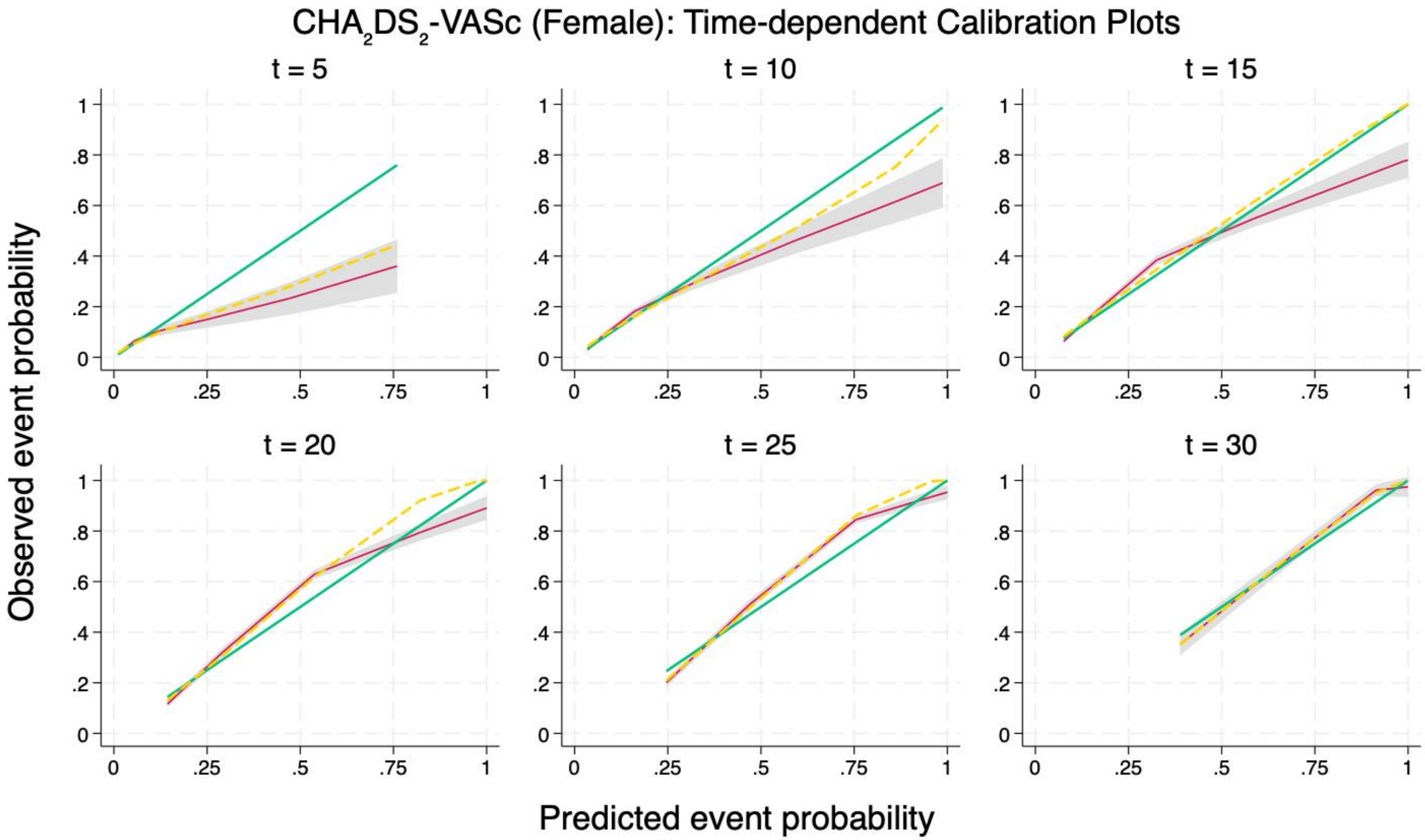
CHA2DS2-VASc (Female): Time-dependent Calibration Plots
t = 5
t = 10
t = 15
t = 20
t = 25
t = 30
Observed event probability
Predicted event probability
0
.2
.4
.6
.8
1
0
.25
.5
.75
1

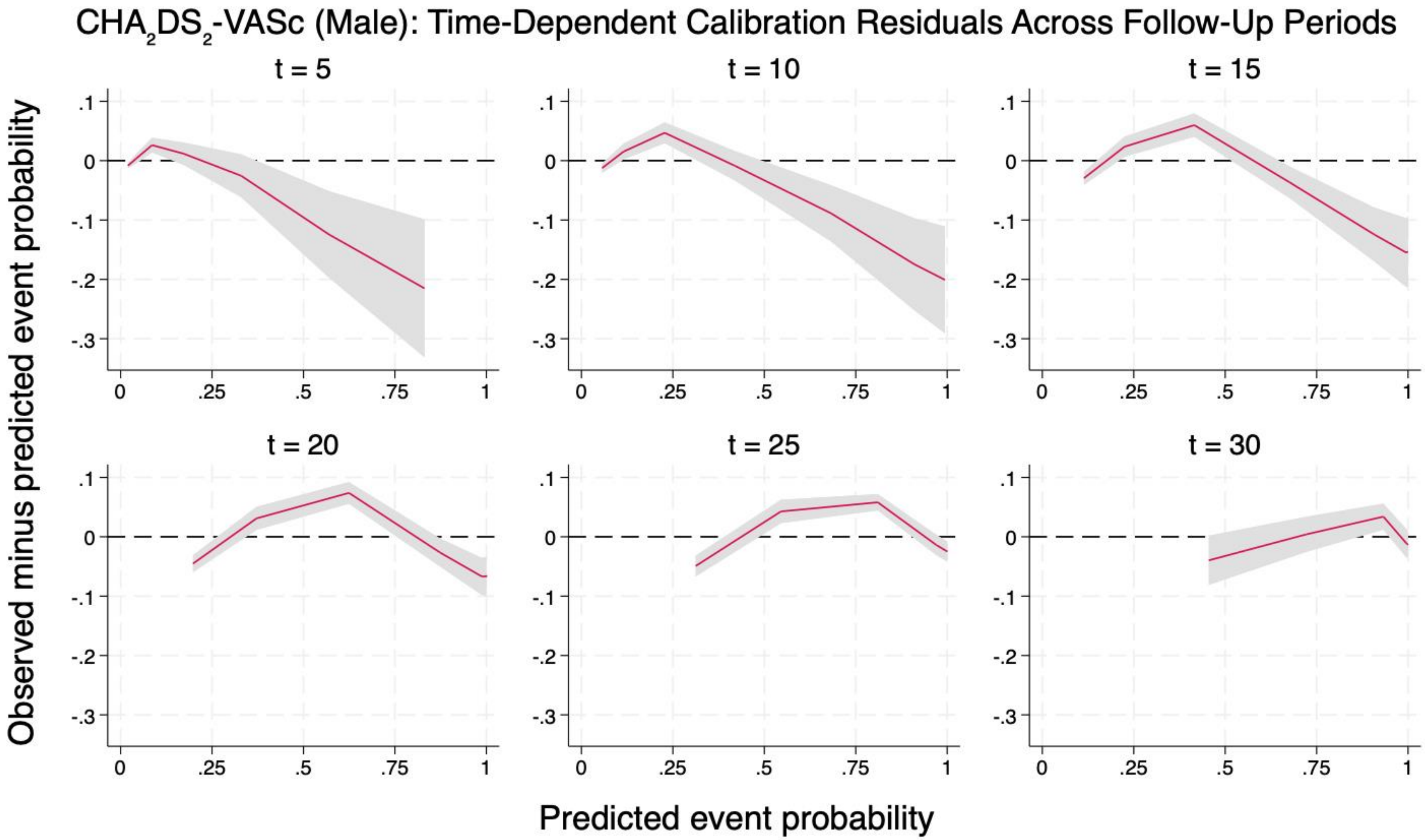

CHA$_2$DS$_2$-VASc (Male): Time-Dependent Calibration Residuals Across Follow-Up Periods
t = 5
t = 10
t = 15
t = 20
t = 25
t = 30
Observed minus predicted event probability
Predicted event probability
.1
0
-.1
-.2
-.3
0
.25
.5
.75
1

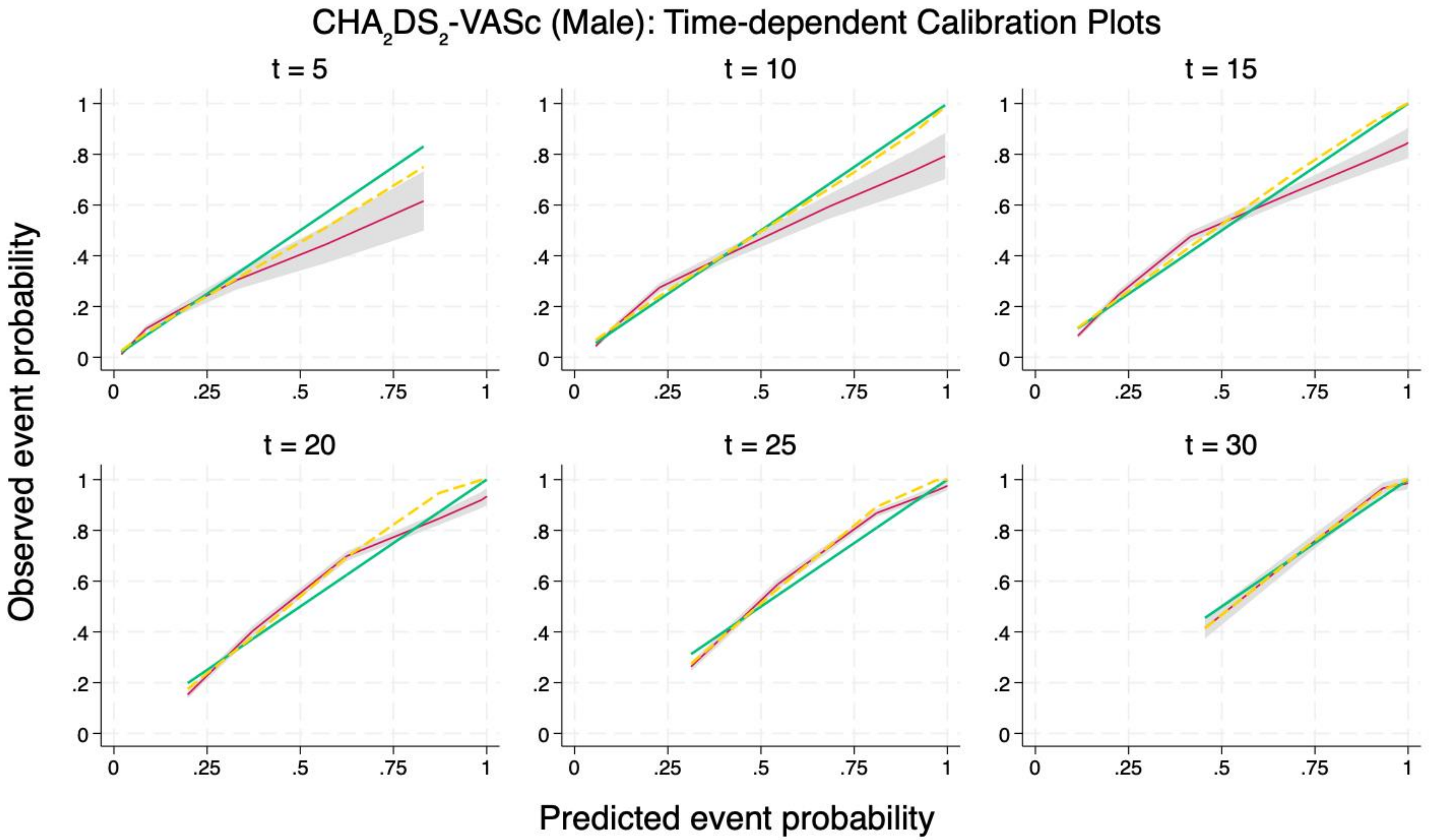

CHA$_2$DS$_2$-VASc (Male): Time-dependent Calibration Plots
t = 5
t = 10
t = 15
t = 20
t = 25
t = 30
Observed event probability
Predicted event probability
0
.2
.4
.6
.8
1
0
.25
.5
.75
1

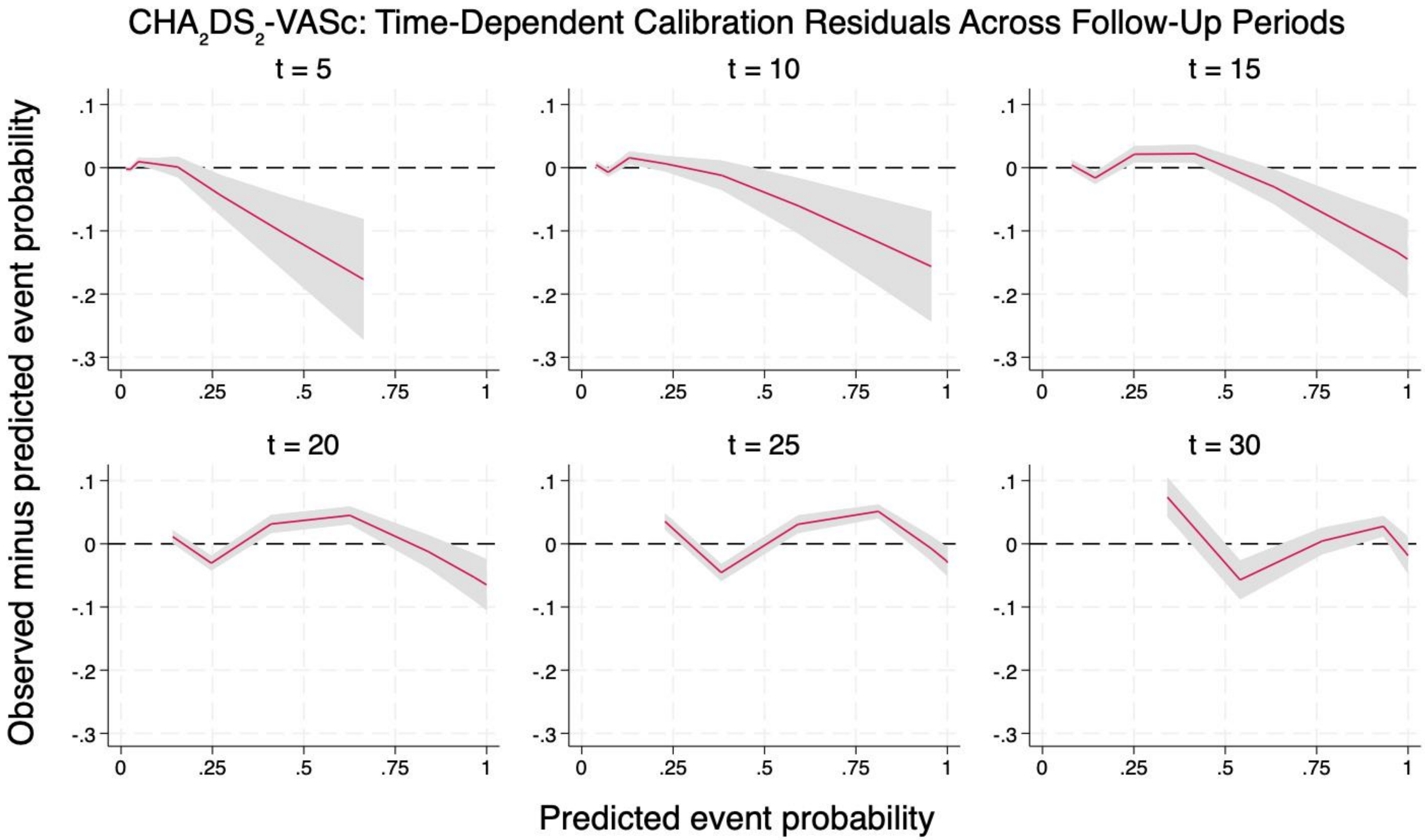

CHA2DS2-VASc: Time-Dependent Calibration Residuals Across Follow-Up Periods
t = 5
t = 10
t = 15
t = 20
t = 25
t = 30
Observed minus predicted event probability
Predicted event probability
.1
0
-.1
-.2
-.3
0
.25
.5
.75
1

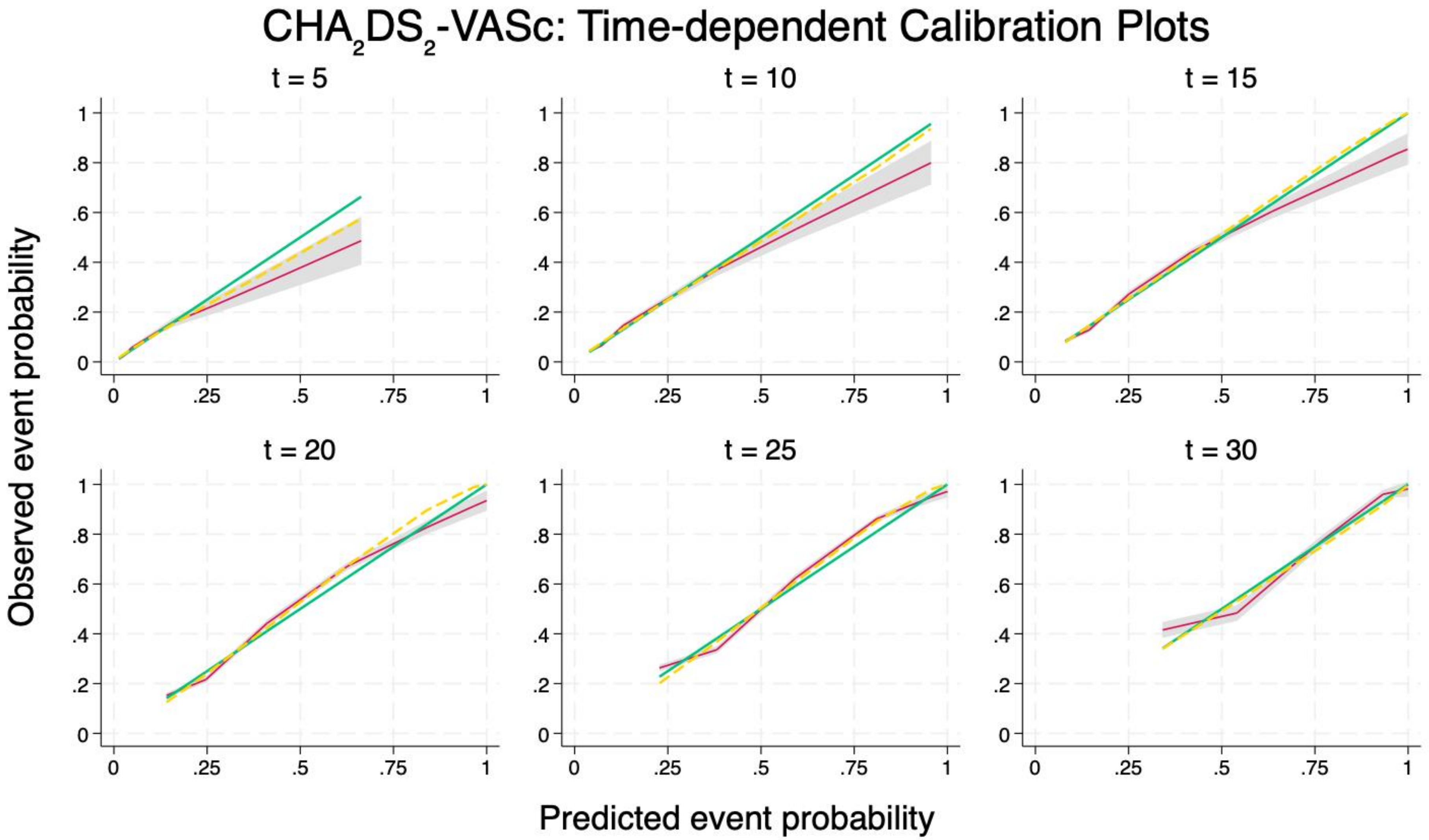
CHA2DS2-VASc: Time-dependent Calibration Plots
t = 5
t = 10
t = 15
t = 20
t = 25
t = 30
Observed event probability
Predicted event probability
0
.2
.4
.6
.8
1
0
.25
.5
.75
1

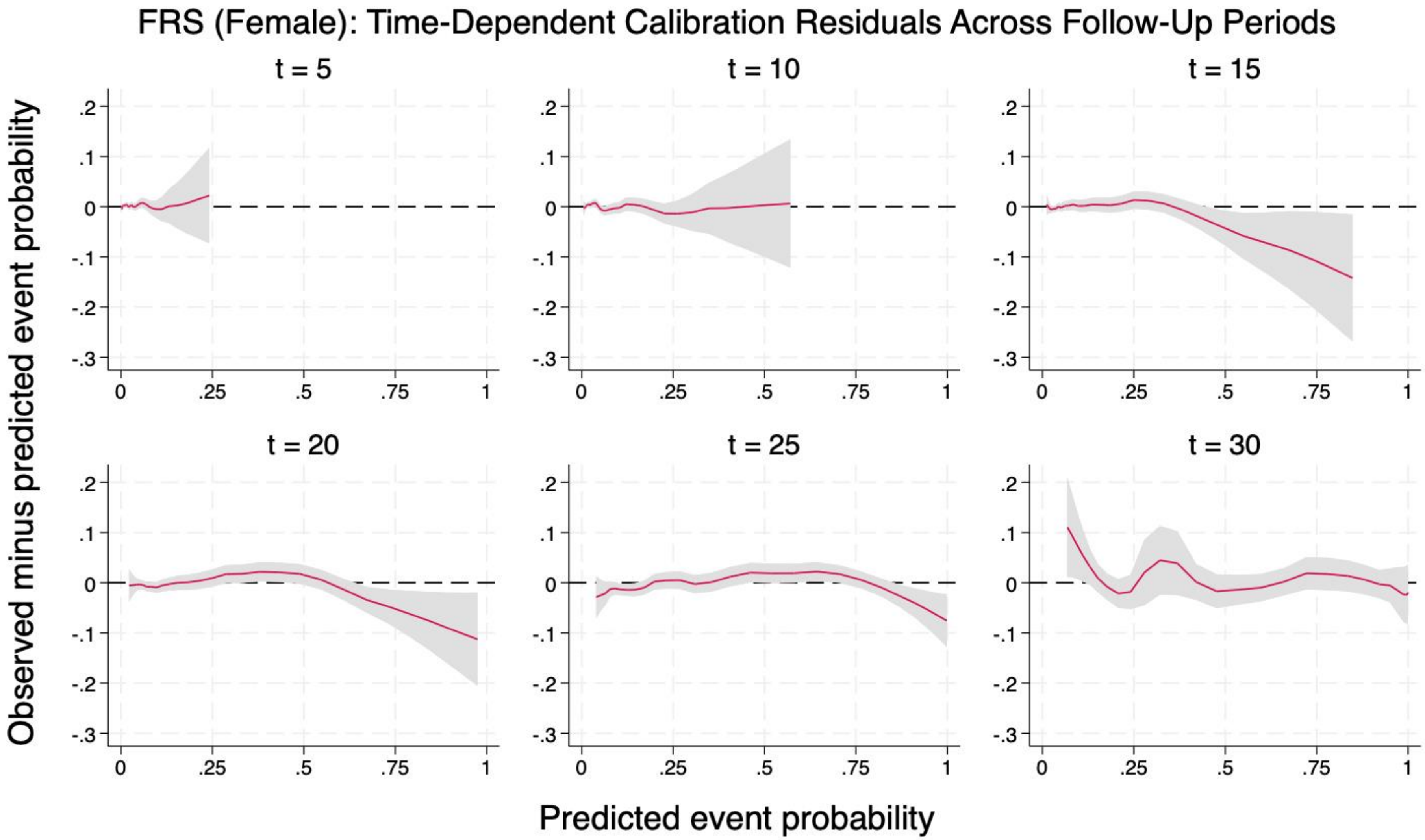

FRS (Female): Time-Dependent Calibration Residuals Across Follow-Up Periods
t = 5
t = 10
t = 15
t = 20
t = 25
t = 30
Observed minus predicted event probability
Predicted event probability
.2
.1
0
-.1
-.2
-.3
0
.25
.5
.75
1

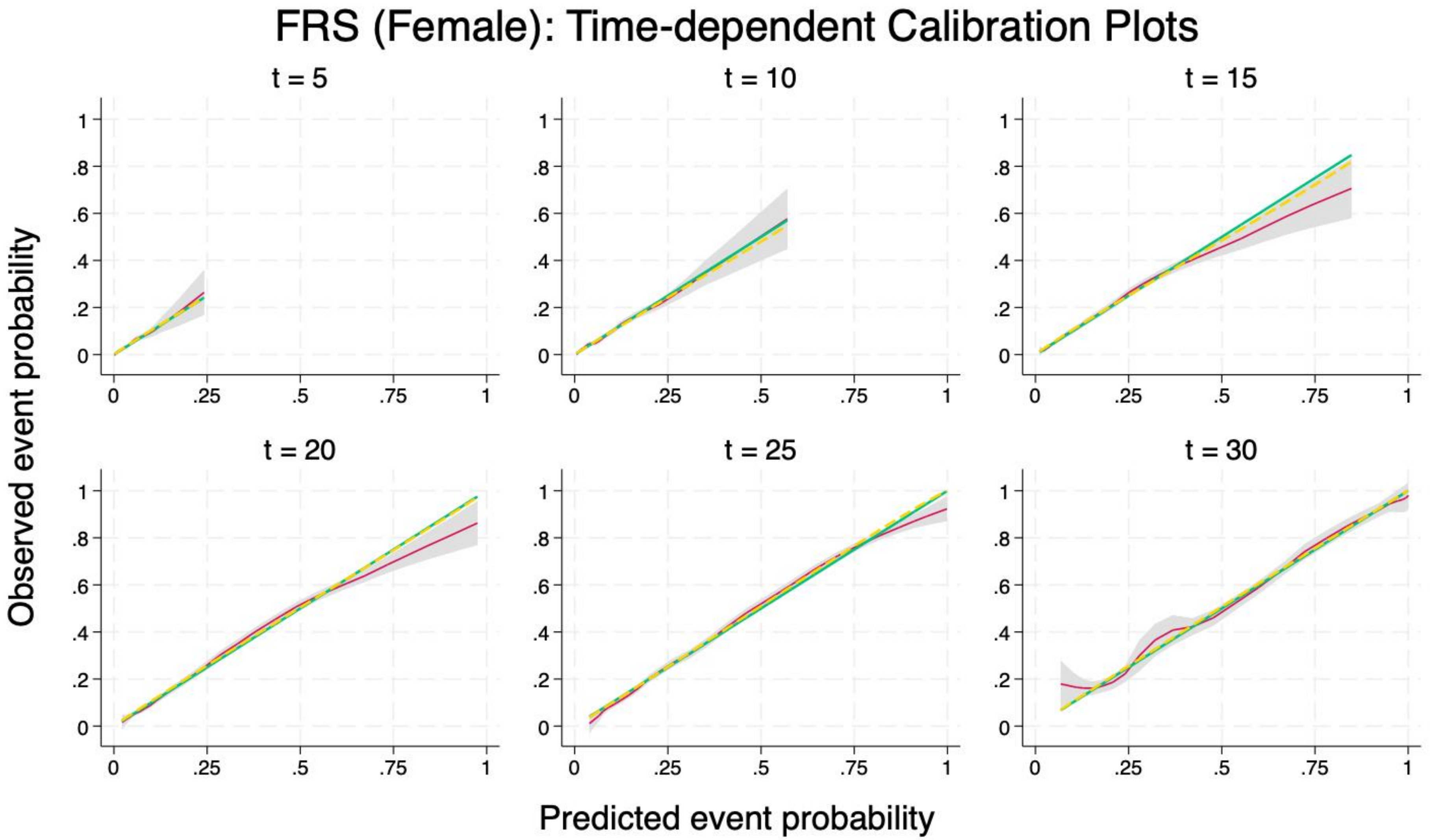
FRS (Female): Time-dependent Calibration Plots
t = 5
t = 10
t = 15
t = 20
t = 25
t = 30
Observed event probability
Predicted event probability
0
.2
.4
.6
.8
1
0
.25
.5
.75
1

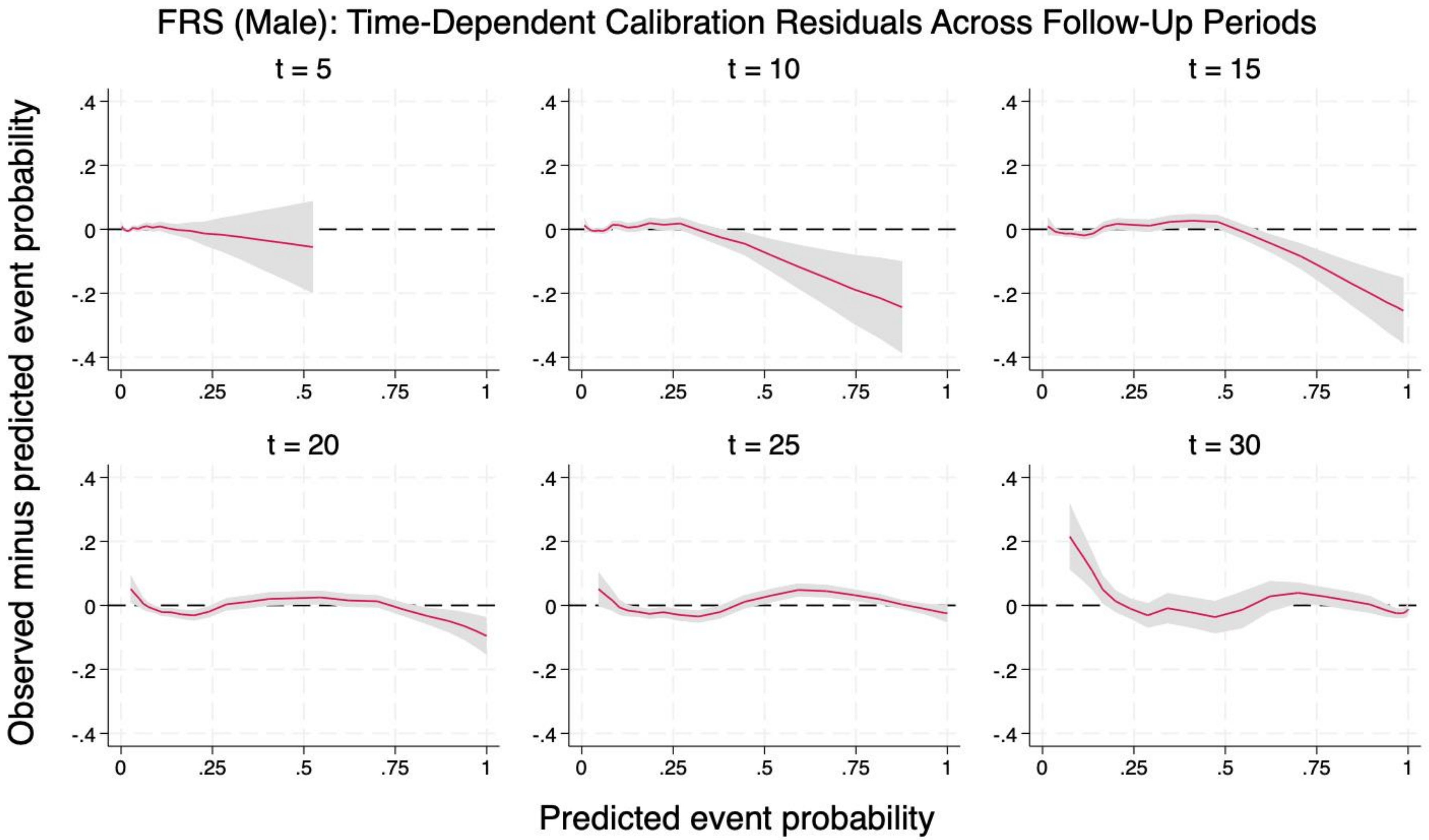
FRS (Male): Time-Dependent Calibration Residuals Across Follow-Up Periods
t = 5
t = 10
t = 15
t = 20
t = 25
t = 30
Observed minus predicted event probability
Predicted event probability
.4
.2
0
-.2
-.4
0
.25
.5
.75
1

FRS (Male): Time-dependent Calibration Plots

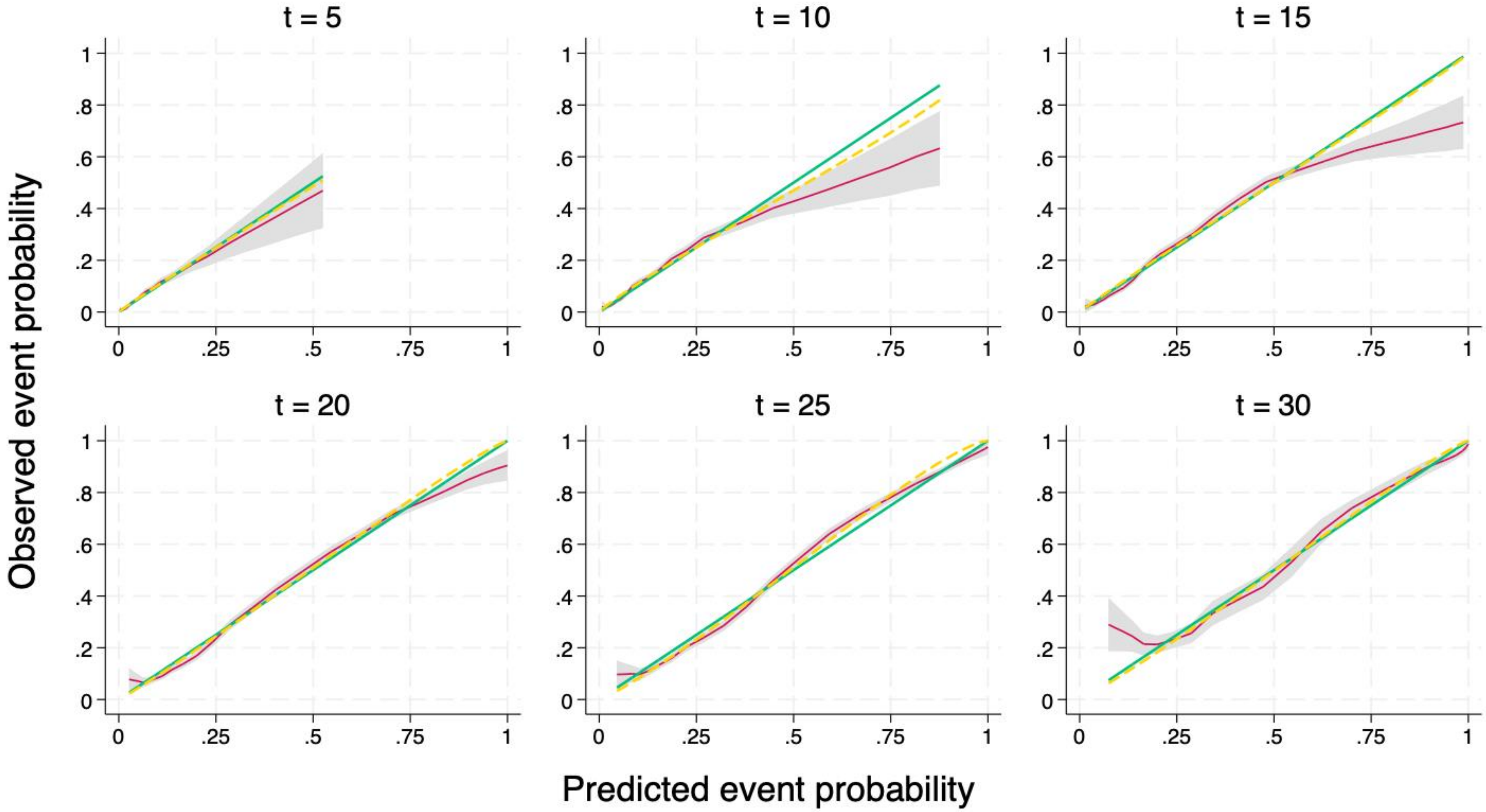

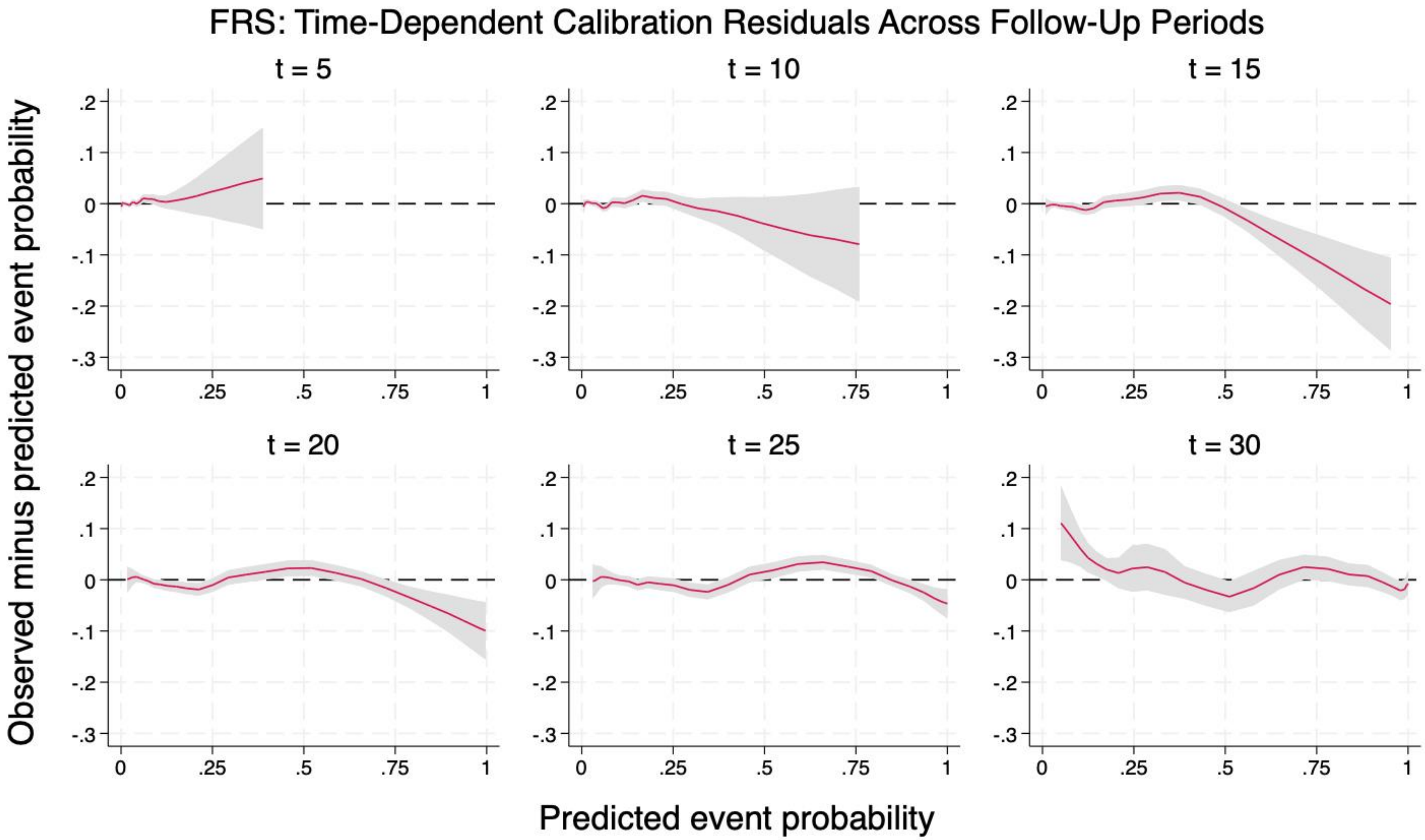

FRS: Time-Dependent Calibration Residuals Across Follow-Up Periods
t = 5
t = 10
t = 15
t = 20
t = 25
t = 30
.2
.1
0
-.1
-.2
-.3
0
.25
.5
.75
1
Observed minus predicted event probability
Predicted event probability

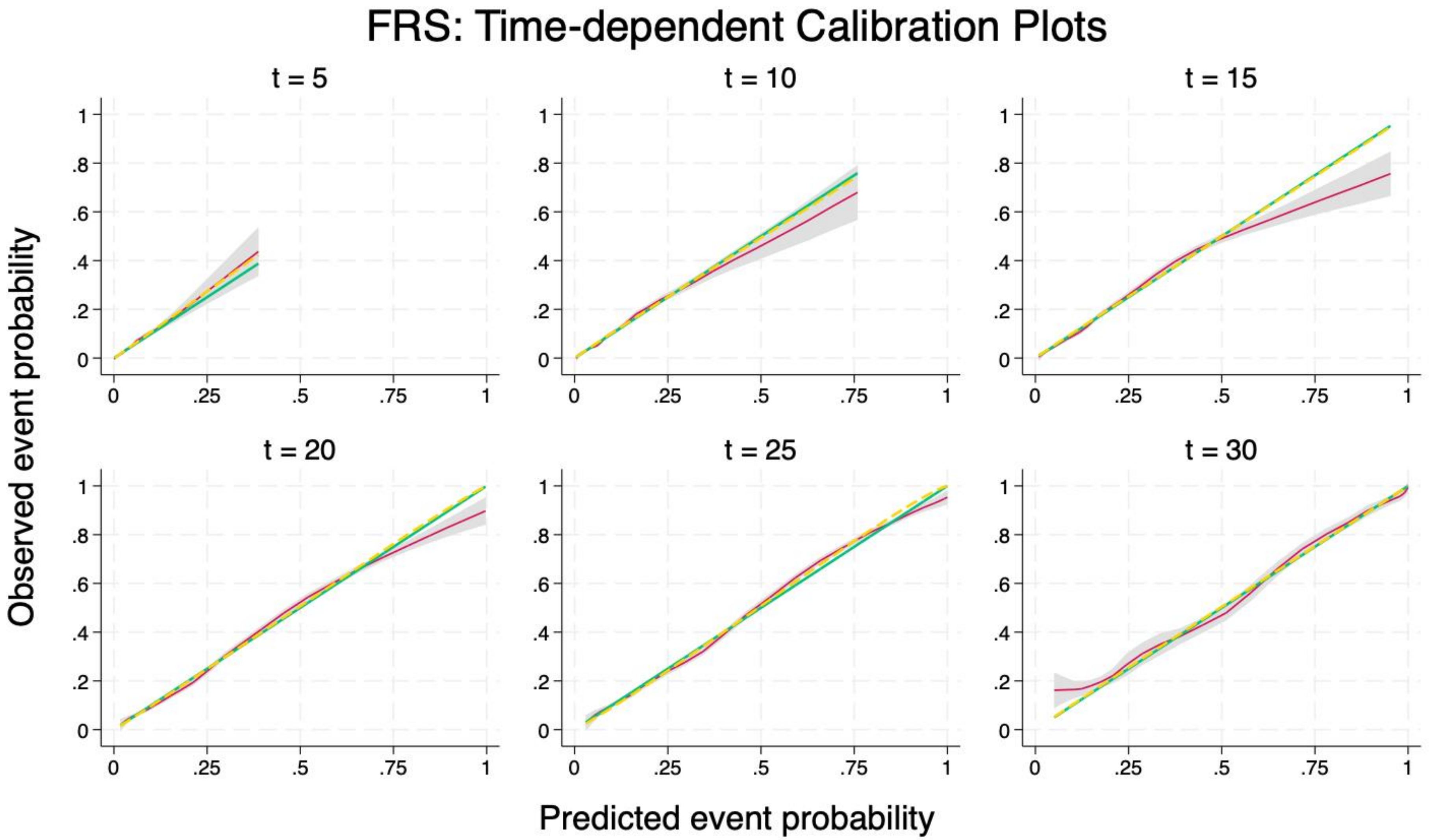

FRS: Time-dependent Calibration Plots
t = 5
t = 10
t = 15
t = 20
t = 25
t = 30
Observed event probability
Predicted event probability
0
.2
.4
.6
.8
1
0
.25
.5
.75
1

References

[1] Collins GS, Moons KGM, Dhiman P, Riley RD, Beam AL, Van Calster B, et al. TRIPOD+AI statement: updated guidance for reporting clinical prediction models that use regression or machine learning methods. BMJ. 2024;385:e078378.
[2] von Elm E, Altman DG, Egger M, Pocock SJ, Gøtzsche PC, Vandenbroucke JP. The Strengthening the Reporting of Observational Studies in Epidemiology (STROBE) statement: guidelines for reporting observational studies. The Lancet. 2007;370:1453-7.
[3] Anstey KJ, Cherbuin N, Herath PM. Development of a new method for assessing global risk of Alzheimer's disease for use in population health approaches to prevention. Prevention Science. 2013;14:411-21.
[4] Barnes DE, Beiser AS, Lee A, Langa KM, Koyama A, Preis SR, et al. Development and validation of a brief dementia screening indicator for primary care. Alzheimer's & Dementia. 2014;10:656-65.e1.
[5] Kivipelto M, Ngandu T, Laatikainen T, Winblad B, Soininen H, Tuomilehto J. Risk score for the prediction of dementia risk in 20 years among middle aged people: a longitudinal, population-based study. The Lancet Neurology. 2006;5:735-41.
[6] Huque MH, Kootar S, Kiely KM, Anderson CS, van Boxtel M, Brodaty H, et al. A single risk assessment for the most common diseases of ageing, developed and validated on 10 cohort studies. BMC Medicine. 2024;22:501.
[7] Walters K, Hardoon S, Petersen I, Iliffe S, Omar RZ, Nazareth I, et al. Predicting dementia risk in primary care: development and validation of the Dementia Risk Score using routinely collected data. BMC Med. 2016;14:6.
[8] Schiepers OJG, Köhler S, Deckers K, Irving K, O'Donnell CA, van den Akker M, et al. Lifestyle for Brain Health (LIBRA): a new model for dementia prevention. International Journal of Geriatric Psychiatry. 2018;33:167-75.
[9] Anatürk M, Patel R, Ebmeier KP, Georgiopoulos G, Newby D, Topiwala A, et al. Development and validation of a dementia risk score in the UK Biobank and Whitehall II cohorts. BMJ Mental Health. 2023;26:e300719.
[10] Lip GY, Nieuwlaat R, Pisters R, Lane DA, Crijns HJ. Refining clinical risk stratification for predicting stroke and thromboembolism in atrial fibrillation using a novel risk factor-based approach: the euro heart survey on atrial fibrillation. Chest. 2010;137:263-72.
[11] D'Agostino RB, Vasan RS, Pencina MJ, Wolf PA, Cobain M, Massaro JM, et al. General Cardiovascular Risk Profile for Use in Primary Care. Circulation. 2008;117:743-53.
